# Prediction of mutation-induced protein stability changes based on the geometric representations learned by a self-supervised method

**DOI:** 10.1186/s12859-024-05876-6

**Published:** 2024-08-28

**Authors:** Shan Shan Li, Zhao Ming Liu, Jiao Li, Yi Bo Ma, Ze Yuan Dong, Jun Wei Hou, Fu Jie Shen, Wei Bu Wang, Qi Ming Li, Ji Guo Su

**Affiliations:** 1 High Performance Computing Center, National Vaccine and Serum Institute (NVSI), Beijing, China; 2 National Engineering Center for New Vaccine Research, Beijing, China; 3 The Sixth Laboratory, National Vaccine and Serum Institute (NVSI), Beijing, China

**Keywords:** Protein stability changes, Mutation, Graph attention network, Self-supervised learning, EXtreme Gradient Boosting model

## Abstract

**Background:**

Thermostability is a fundamental property of proteins to maintain their biological functions. Predicting protein stability changes upon mutation is important for our understanding protein structure–function relationship, and is also of great interest in protein engineering and pharmaceutical design.

**Results:**

Here we present mutDDG-SSM, a deep learning-based framework that uses the geometric representations encoded in protein structure to predict the mutation-induced protein stability changes. mutDDG-SSM consists of two parts: a graph attention network-based protein structural feature extractor that is trained with a self-supervised learning scheme using large-scale high-resolution protein structures, and an eXtreme Gradient Boosting model-based stability change predictor with an advantage of alleviating overfitting problem. The performance of mutDDG-SSM was tested on several widely-used independent datasets. Then, myoglobin and p53 were used as case studies to illustrate the effectiveness of the model in predicting protein stability changes upon mutations. Our results show that mutDDG-SSM achieved high performance in estimating the effects of mutations on protein stability. In addition, mutDDG-SSM exhibited good unbiasedness, where the prediction accuracy on the inverse mutations is as well as that on the direct mutations.

**Conclusion:**

Meaningful features can be extracted from our pre-trained model to build downstream tasks and our model may serve as a valuable tool for protein engineering and drug design.

**Supplementary Information:**

The online version contains supplementary material available at 10.1186/s12859-024-05876-6.

## Background

The biological function of a protein is largely determined by its tertiary structure and the associated thermodynamic stability [1], and thus the mutation of residues may affect protein function through changing its structural stability [2]. Previous studies have revealed that many human disorders were attributed, at least partially, to protein stabilization or destabilization caused by missense mutations [3]. Therefore, accurate prediction of protein thermostability changes resulted by residue substitution is crucial for better understanding protein function, and assists in predicting deleterious mutations responsible for human diseases [4]. In addition, improving thermodynamic stability is one of the common requirements in protein engineering for biopharmaceuticals [5]. Protein stability optimization is important for the development, manufacture, storage and clinical utilization of biological products [6]. Consequently, effective prediction of mutation-induced protein stability changes also has significant applications in bioindustry.

The thermodynamic stability of a protein is usually represented as the difference in free energy between the folded and unfolded states (ΔG) [7], which is determined by the inter-residue interactions within the protein structure as well as the interactions between the protein and the aqueous solution [8]. Upon the mutation of a residue, the interactions involving the mutated residue will be altered, which results in the changes in the thermodynamic stability (ΔΔG) of the protein [9]. Experimentally evaluating the effects of mutations on protein stability is expensive and time-consuming [10]. Especially, protein stability optimization usually involves the screening of numerous possible mutations, and thus experimental measurement is a huge task, if not impossible. Computational methods, such as molecular dynamics (MD) simulations [11], empirical potential-based calculations, and machine learning (ML) models, provide a complementary approach to experiments in characterizing protein stability changes [12, 13]. These computational methods are relatively faster and can also be applied to predict the effects of mutations that are difficult or impossible investigated by experiments [14]. MD simulations in combination with the molecular mechanics-generalized Born surface area (MMGBSA) [15] or free energy perturbation (FEP) [16] methods have been largely used in computing the mutation-induced protein stability changes, and the FEP method is believed to be one of the most accurate computational methods to date. However, MD simulations require significant computational resources. Empirical potential-based methods, for example, FoldX [17] and Rossetta [18], are computationally more efficient and usually applied in large-scale mutation screening, but the accuracy of this kind of methods is limited.

With the rapid developments in artificial intelligence, many effective methods with low computational cost based on machine learning (ML) models have been proposed for predicting the impact of mutations on protein thermodynamic stability [19]. In these ML-based methods, a variety of algorithms have been applied, ranging from the classical decision tree, random forests, support vector machine and artificial neural network to the newest deep learning approaches. The information used in these prediction methods includes protein sequence [20], molecular evolution [21], tertiary structure [22], physical and statistical energies [12], or combination of them [12], in which three-dimensional structure and the related features were taken into accounts for most of the leading ML-based models [23]. ML-based methods are powerful in extracting the vital information that determines the changes in protein stability upon point mutation, however, the performance of these methods is relied on large amounts of high-quality experimental data for model training [24]. Unfortunately, the number of cases in the available training dataset, for example, the most commonly used Q3421 [25], is currently still limited, and a large portion of the previously published ML-based methods for predicting mutation-induced stability changes are prone to overfitting [26]. These methods only perform well on the cases similar to the mutations in training sets, but cannot be generalized well to the unseen protein structures. Besides that, the available experimental datasets are unbalanced, which are dominated by destabilizing cases, and therefore most of the ML-based prediction methods are also biased. The performance of these methods on the inverse mutations is not as well as that of the direct mutations in the test dataset [27]. It is highly desirable, but also challenging, to develop a ML model with good generalizability and unbiasedness.

In the present work, inspired by the study of Liu et al. [28], we designed a novel deep learning framework, called mutDDG-SSM, to predict the changes in protein stability upon residue mutations. In our method, the essential features underlying the interactions of a residue with its surrounding neighbors were extracted from a large number of known protein structures by using a graph attention network (GAT) [29] with a self-supervised learning scheme. The task in the self-supervised learning process is to predict the original conformation of a given perturbed protein, in which the side chain of a residue was randomly rotated. By this way, the intrinsic inter-atomic interactions between residues were extracted. Then, the learned representations were applied in an eXtreme Gradient Boosting (XGBoost) [30] model to predict the impacts of residue mutations on protein stability. In our mutDDG-SSM method, the atomic representations were learned from large-scale unlabeled protein structures across divergent classes. These learned representations can be used for different downstream tasks including but not limited to protein stability prediction, protein–protein binding affinity prediction, and so on. The self-supervised learning scheme by large amounts of protein structures can help to avoid the overfitting and unbalance problems commonly faced by previously reported models. The XGBoost model used in the latter training stage also has the advantage of avoiding overfitting compared to other ML models. These schemes enable our method to be better generalizability and unbiasedness. Tests on the widely used independent S^sym^ [31], S350 [32], S611 [33], S276 [34] and S669 [19] datasets demonstrated that our method achieved one of the best unbiased performance for the direct and the corresponding inverse mutations compared to other methods tested. Given that the majority of protein mutations seen in nature are single nucleotide variants (SNVs) [35], which are usually associated in human diseases [36], we also evaluated the performance of mutDDG-SSM by classifying the mutations into SNVs and non-SNVs. Then, myoglobin [37] and p53 [38] were used as case studies to illustrate the effectiveness and unbiasedness of mutDDG-SSM in predicting mutation-induced protein stability changes.

## Methods

### The architecture of mutDDG-SSM

**Fig. 1:**
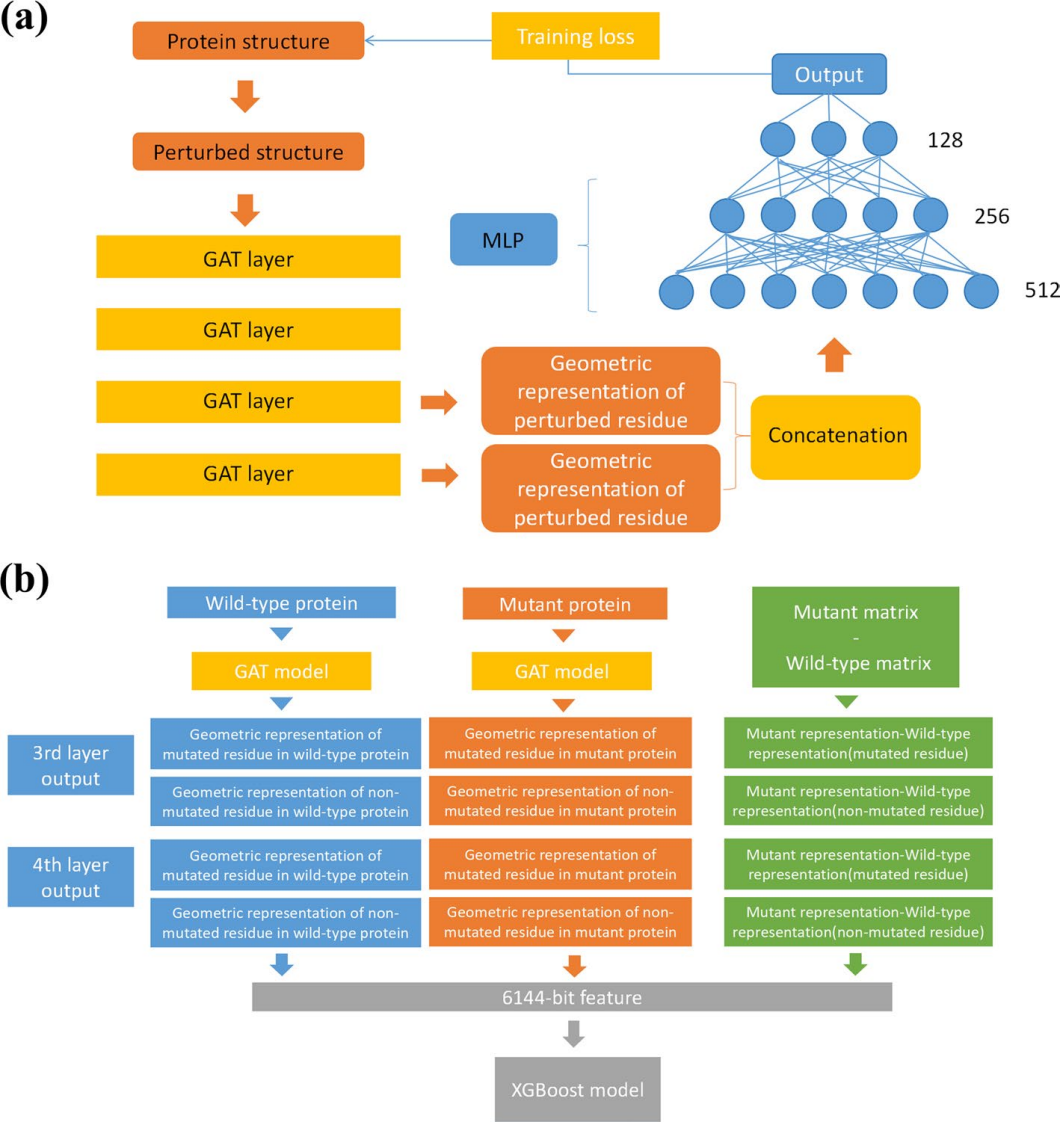
Overall view of the mutDDG-SSM framework. **a** The self-supervised GAT encoder to extract the geometric representations encoded in the protein structure. **b** The XGBoost predictor to evaluate the changes in protein stability upon residue mutation by using the features learned by the GAT encoder

The proposed mutDDG-SSM consists of two parts: the protein structural feature extractor and the mutation-induced protein stability change predictor, as shown in Fig. 1. The GAT model, which is excellent in extracting geometric features, was used as the feature extractor. A self-supervised learning scheme was designed for the training of the GAT model to extract the representation of the intrinsic inter-residue interactions within protein structure. The geometric representations extracted from the pre-trained GAT model were inputted to the second part of mutDDG-SSM to predict the changes of protein stability caused by residue mutation. The XGBoost model was applied as the protein stability change predictor owing to its good performance in avoiding overfitting problem.

### Design of tasks for the self-supervised learning scheme

To learn the intrinsic features encoding the atomic interactions of the residue with its surrounding neighbors in protein structures, a self-supervised learning scheme was designed. A large-scale non-redundant and high-resolution protein structures were used for the self-supervised learning. For a given protein structure, the side chain of a randomly selected residue was perturbed, and the task for the self-supervised training was to predict the original conformation of the perturbed residue within the protein. By this way, the essential geometric features underlying the atomic interactions of the residue with its surroundings in the protein structure were captured.

Specifically, in residue perturbation, the values of the torsion angles of the perturbed residue were sampled according to the distribution and probability provided by the Dunbrack backbone-dependent rotamer library [39]. Dunbrack rotamer library, which is derived from the analysis of large-scale protein native structures, provides the probability of discrete side-chain torsion angles of a residue dependent on the backbone dihedral angle values. Based on the Dunbrack rotamer library, we randomly alter the residue conformation within the protein structure to adopt a new conformation. Based on the perturbed conformation, the task of the self-supervised learning is to predict the original conformation of the residue within the protein. For glycine and alanine, whose side chain only consist of hydrogen or methyl groups, the original conformation of the side chain remains unchanged.

### Representation extraction by the self-supervised learning scheme with a graph attention network model

The GAT model was used for the self-supervised learning to capture the geometric features encoding the inter-residue interactions within the protein structure. GAT model describes protein structures as graphs, in which atoms in the structure were represented by nodes and the interactions between atoms were represented as edges. The attention mechanism of the model enables the features of the nodes to be updated using their neighborhood’s features with different weights. The GAT model has been widely applied to bioinformatics studies, such as protein–protein binding prediction [40], protein–ligand interaction prediction [41], and protein function prediction [42].

Specifically, for a given protein structure with a perturbed residue, a graph was firstly built, in which the atoms in the protein were represented by nodes and the interactions between atoms were simplified by edges [29]. Only the heavy atoms in the structure, including carbon, nitrogen, oxygen, and sulfur, were considered for the graph construction. Besides that, to reduce the computation complexity, only the atoms within a radius of 12.0 Å to the center of the perturbed residue were taken into account to build the graph. In the case of the residue having only a portion of its atoms falling within the 12.0 Å range, all the atoms belonging to the residue were retained in the graph. If the distance between two nodes is less than a threshold (3.0 Å was adopted), an edge was assumed to exist between these them. The nodes in the graph were attached with attributes that describe the properties of the atoms. In this study, for each node, 36-dimensional attributes were considered, which include:

1. The atom type, i.e., carbon, nitrogen, oxygen, or sulfur, represented by one-hot encoding;
2. The type of the amino acid, to which the atom belongs, represented by 20-dimensional one-hot encoding;
3. The type of DSSP secondary structure [43] involving the atom, i.e., alpha-helix, isolated beta-bridge residue, strand, 3_10_-helix, pi-helix, turn, bend or none, encoded by one-hot codes;
4. A one-hot encoding of the atom that was perturbed or not;
5. A binary attribute representing whether the solvent accessible surface area (SASA) [44] of the atom is greater than 0 or not (denoted by 1 or 0, respectively);
6. A binary feature indicating whether the atom is a Cα atom or not, represented by 1 or 0, respectively.

The node figures and edges constructed from the protein structure upon residue perturbation were fed into the GAT model. GAT updates the node features using a self-attention mechanism. The normalized attention coefficients were calculated according to the formula described in the reference [29]. Then, based on the normalized attention coefficients, the node features were updated by weighted summation of the features from its neighboring nodes. In this study, a multi-head attention was used in the self-attentional layer, in which the feature representation output was the concatenation of the output from eight independent self-attention operations. To better extract the representations encoding the inter-residue interactions within the protein structure, four eight-head attention layers were stacked, and the output representations from the third and fourth layers were concatenated as the final geometric representations.

Based on the geometric representations extracted by the GAT model, a three-layer perceptron network was employed to predict the original conformation of the perturbed residue within the protein. Considering that directly predicting the absolute value of the atomic positions increases the difficulty, our model aimed to predict the deviation of the atoms between the perturbed and original positions, expressed by

1 $$\Delta {d}_{i}=MLP\left({\overrightarrow{g}}_{i}\right)$$

where $\Delta {d}_{i}$ is the predicted deviation of the perturbed atom $i$ from its native position, ${\overrightarrow{g}}_{i}$ is the geometric representations of atom $i$, and $MLP$ stands for the multi-layer perceptron network. The real value for the deviation of the perturbed atom $i$, denoted as ${\Delta d}_{i}^{real}$, was computed by the root mean square deviation (RMSD) of the atomic coordinates between the perturbed and original conformations. Then, the loss function of the GAT model for the self-supervised learning was defined as the mean square error between the predicted and real deviations of the perturbed atoms, given by

2 $$L=\frac{1}{{N}_{p}}\sum_{i\in {N}_{p}}{\left(\Delta {d}_{i}- {\Delta d}_{i}^{real}\right)}^{2}$$

where ${N}_{p}$ is the number of atoms in the perturbed residue. The GAT model was implemented in the framework of PyTorch [45] and PyTorch Geometric [46].

In summary, based on the self-supervised learning scheme with the GAT model, the geometric representations encoding the inter-residue interactions were extracted from the large-scale non-redundant protein structures.

### Prediction of mutation-induced protein stability changes by eXtreme Gradient Boosting model

Based on the geometric representations extracted by the self-supervised learning scheme, the changes in protein stability upon residue mutation were predicted using a XGBoost model. XGBoost model has good performance in avoiding overfitting compared to other ML models.

The geometric representations of both the original protein and its mutant were first generated by using the trained self-supervised GAT model. As given by Eq. (3), the geometric representations of each atom were taken from the third and fourth attention layers of the GAT model. The representations of the atoms in the mutated residue and those of the other atoms within a radius of 12.0 Å to the center of the mutated residue were represented by.

3 $$\left\{ {\vec{h}_{i}^{^{\prime}\left( L \right)} ,i \in A_{mo} } \right\}, \left\{ {\vec{h}_{j}^{^{\prime}\left( L \right)} ,j \in A_{no} } \right\}, \left\{ {\vec{h}_{k}^{^{\prime}\left( L \right)} ,k \in A_{mm} } \right\}, \left\{ {\vec{h}_{l}^{^{\prime}\left( L \right)} ,l \in A_{nm} } \right\}$$

where $L = 3{ }\;or{ }\;4$ that stands for the representations derived from the third and fourth layers of the GAT model, respectively; ${A}_{mo}$, ${A}_{no}$, ${A}_{mm}$, and ${A}_{nm}$ denote the atoms belonging to the mutated residues in the original protein, the non-mutated residues within a 12.0 Å radius in the original protein, the mutated residues in the mutant protein, and the non-mutated residues within a 12.0 Å radius in the mutant protein, respectively. Then, both the maximum and mean values over the atoms in the mutated residue were computed to represent the geometric features of the mutated residue. The maximum and mean values of the non-mutated atoms were calculated to represent the geometric features of the environment around the mutated residue. The differences in the maximum and mean values between mutated and non-mutated atoms were also computed to represent the distinct geometric features of the mutated residue in comparison of its environment. All these geometric representations both for the original protein and its mutant were concatenated together, and standardized by removing the mean and scaling to unit variance, which can be expressed as

4 $$\begin{aligned} \vec{F}_{mo}^{\left( L \right)} = & \left\{ {\vec{h}_{i}^{^{\prime}\left( L \right)} ,i \in A_{mo} } \right\}_{max} \parallel \left\{ {\vec{h}_{i}^{^{\prime}\left( L \right)} ,i \in A_{mo} } \right\}_{mean} \\ \vec{F}_{no}^{\left( L \right)} = & \left\{ {\vec{h}_{j}^{^{\prime}\left( L \right)} ,j \in A_{no} } \right\}_{max} \parallel \left\{ {\vec{h}_{j}^{^{\prime}\left( L \right)} ,j \in A_{no} } \right\}_{mean} \\ \vec{F}_{mo - no}^{\left( L \right)} = & \vec{F}_{mo}^{\left( L \right)} - \vec{F}_{no}^{\left( L \right)} \\ \vec{F}_{mm}^{\left( L \right)} = & \left\{ {\vec{h}_{k}^{^{\prime}\left( L \right)} ,k \in A_{mm} } \right\}_{max} \parallel \left\{ {\vec{h}_{k}^{^{\prime}\left( L \right)} ,k \in A_{mm} } \right\}_{mean} \\ \vec{F}_{nm}^{\left( L \right)} = & \left\{ {\vec{h}_{l}^{^{\prime}\left( L \right)} ,l \in A_{nm} } \right\}_{max} \parallel \left\{ {\vec{h}_{l}^{^{\prime}\left( L \right)} ,l \in A_{nm} } \right\}_{mean} \\ \vec{F}_{mm - nm}^{\left( L \right)} = & \vec{F}_{mm}^{\left( L \right)} - \vec{F}_{nm}^{\left( L \right)} \\ \vec{F}^{\left( L \right)} = & std\left( {\vec{F}_{mo}^{\left( L \right)} \parallel \vec{F}_{no}^{\left( L \right)} \parallel \vec{F}_{mo - no}^{\left( L \right)} \parallel \vec{F}_{mm}^{\left( L \right)} \parallel \vec{F}_{nm}^{\left( L \right)} \parallel \vec{F}_{mm - nm}^{\left( L \right)} } \right) \\ \end{aligned}$$

where $L = 3{ }\;or{ }\;4$ that stands for the representations derived from the third and fourth layers of the GAT model, respectively; $std\left(\cdots \right)$ means the standardization operation for each feature over the whole training dataset; The subscripts ‘max’ and ‘mean’ denote the max-pooling and mean-pooling operations over the atoms in the corresponding atom sets, respectively; $\parallel$ represents the concatenation of the geometric representations. Then, these geometric representations from the third and fourth layers of GAT model were concatenated together and fed into the XGBoost model, and the change of protein stability $\Delta \Delta G$ caused by residue mutations was outputted, i.e.,

5 $$\Delta \Delta G=XGBoost\left({\overrightarrow{F}}^{\left(3\right)}\parallel {\overrightarrow{F}}^{\left(4\right)}\right)$$

In our study, the XGBoost model was trained and tested, respectively, by using independent protein datasets with available experimental mutation-caused stability change data.

### The training techniques of mutDDG-SSM

The architecture of the proposed mutDDG-SSM framework is composed of two separate components, i.e., the self-supervised GAT encoder to extract the geometric representations of the protein structure and the XGBoost predictor to predict the changes in protein stability upon residue mutation by using the features learned by the GAT encoder. These two parts were trained separately by using different datasets. The GAT model was trained on a large-scale unlabeled high-resolution protein structure dataset via a self-supervised learning scheme. The XGBoost model was trained on the labeled dataset with available mutation-induced stability change values obtained from experiments.

The GAT model was trained by using batch gradient descent approach with the Adam optimizer, The batch size was 128 and the learning rate was set to 0.001. For the training of the XGBoost model, the training set was divided into ten subsets, and then ten separate models were optimized. Each model was trained on nine subsets and the remaining one subset was utilized as the validation set. The average value of the outputs of these optimized ten models was taken as the final output. The hyperparameters of XGBoost model were chosen from $\text{n}\_\text{estimators}\in \left\{10000, 20000, 30000\right\}$, $\text{max}\_\text{depth}\in \left\{5, 6, 7\right\}$, $\text{subsample}\in \left\{0.6, 0.7, 0.8\right\}$, $\text{colsample}\_\text{bytree}\in \left\{0.55, 0.56, 0.57\right\}$ and $\text{learning}\_\text{rate}\in \left\{0.02, 0.05, 0.1\right\}$ by grid search procedure. The best hyperparameters were determined to yield the highest performance of the model.

### Dataset preparation

To train the geometric feature extractor, i.e. the GAT model, via the self-supervised learning approach, a large-scale training dataset was constructed from the Protein Data Bank (PDB) by using the PISCES [47] server. PISCES provided a tool to screen non-redundant and high-resolution protein structures from PDB based on sequence identity and structural quality. Considering that the quality of protein structures in the training dataset influences the performance of the model [48], only the protein structures meeting the following criteria were selected and included in the dataset:

1. The structure was obtained by X-ray crystallography with an R-value less than 0.25;
2. The resolution of the structure was below 2.0 Å;
3. The protein length was within the range of 40–500 amino acids and devoid of any break or missing of residues;
4. Sequence identity among the proteins in the dataset was below 25%.

A total of 5893 protein structures were collected. After adding missing atoms by using PDBFixer (https://github.com/openmm/pdbfixer) [49], these protein structures were included in the dataset for the training and validation of the GAT model. From the collected dataset, 5238 protein structures were randomly partitioned into training set, which were used to train the protein geometric feature extractor, namely the GAT model, using the self-supervised learning scheme as discussed above. The remaining 655 protein structures from the dataset were taken as the validation set. The PDB accession code, along with the organism and structural class, of the protein structures in the training and validation set were listed in Supplementary Table 1. During the training and validation of the GAT model, residue perturbation was performed 2000 times for each protein structure, and therefore 10,476,000 and 1,310,000 data were used, in fact, for the training and validation of the GAT model, respectively.

The mutation-induced $\Delta \Delta G$ predictor, namely the XGBoost model, was trained using the widely adopted Q3421 dataset. The prediction performance of the model was tested on several commonly used datasets, including S^sym^, S350, S611, S276 and S669, and two protein cases, i.e., myoglobin and p53, to explore the accuracy and generalizability of our model.

Q3421 contains 3421 single-point mutations with experimentally measured $\Delta \Delta G$ values from 150 proteins. This dataset contains 14 proteins that are also included in the S^sym^ test dataset. Therefore, these 14 proteins along with the related mutation data were removed from Q3421, and then a dataset consisting of 3213 mutations, called Q3213, was obtained. Furthermore, to balance the stabilizing and destabilizing mutations in the dataset, the inverse mutation assigned with opposite $\Delta \Delta G$ value was created for each direct mutation in the dataset. The structure of the protein with inverse mutations was constructed using Rossetta [50]. By this way, the Q3213 dataset was augmented to a balanced dataset containing 6426 mutation data.

For the test datasets, S^sym^ is composed of 684 mutation data, including 342 direct mutations with available experimental $\Delta \Delta G$ values as well as the corresponding inverse mutations. S350 consists of 350 experimental mutations from 67 different proteins. S611 is an extension of S350, which includes 611 direct and inverse mutations. S276 contains 276 experimental $\Delta \Delta G$ values from 37 different proteins, and S669 consists of 669 experimental data from 94 proteins. These datasets were used to test the performance of the model trained by Q3213. After removing the shared mutations in the training set Q3213, the number of mutation data points in S350 was decreased to 203, named as S203, and that of S611 was reduced to 347, called S347, with 203 direct and 144 inverse mutations. For S276 and S669, the number of experimental values was reduced to 254 and 615 (called S254 and S615 respectively) after removing the same mutations shared with Q3213. Then, S254 and S615 were augmented to include the corresponding inverse mutations of the experimental data. The PDB accession code and mutation information of the protein structures in S203, S347, S254 and S615 were listed in Supplementary Tables 2, 3, 4 and 5, respectively. Myoglobin and p53 that were used as case studies in our study contain 134 and 42 experimental mutations, respectively. The mutations in myoglobin and p53 were listed in Supplementary Tables 6 and 7. In our study, the corresponding inverse mutations were also constructed and included in the datasets for these two proteins.

### Performance measures

In this study, the performance of mutDDG-SSM model was evaluated and compared with other previously developed methods using the Pearson correlation coefficient (PCC), root mean square error (RMSE), and mean absolute error (MAE) between the predicted $\Delta \Delta G$ values and the experimental data. The accuracy (ACC), sensitivity (SEN), specificity (SPE) and Matthews correlation coefficient (MCC) were applied to evaluated the performance of the model in classifying the stabilizing and destabilizing mutations. In addition, to evaluate the biasedness of the model in predicting the ΔΔG values of direct and inverse mutations, ${r}^{d-i}$ and $<\delta >$ were calculated by

6 $${r}^{d-i}=PCC({\Delta \Delta G}_{direct}, {\Delta \Delta G}_{inverse})$$

7 $$<\delta >= \frac{1}{n}{\sum }_{i=1}^{n}({\Delta \Delta G}_{direct}+{\Delta \Delta G}_{inverse})$$

here ${\Delta \Delta G}_{direct}$ and $\Delta \Delta {G}_{reverse}$ represent the predicted values for the direct and inverse mutations, respectively. n is the number of data points.

## Results

### Performance in predicting mutation-induced protein stability changes

**Fig. 2:**
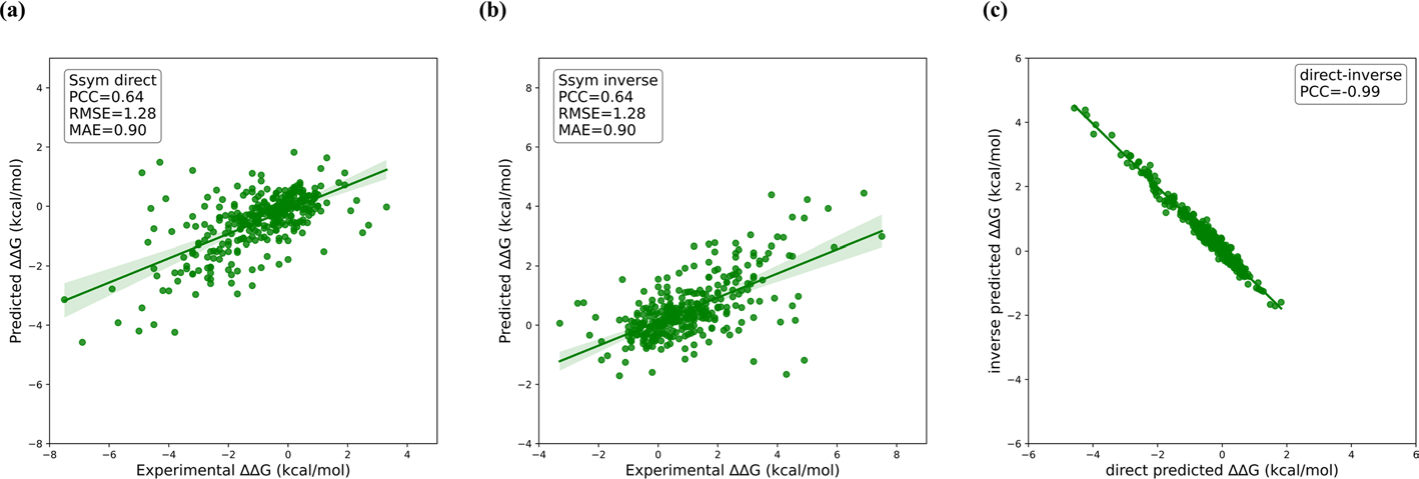
The performance of mutDDG-SSM in predicting $\Delta \Delta G$ on the S^sym^ dataset. **a** The performance of mutDDG-SSM on direct mutations. **b** The performance of mutDDG-SSM on inverse mutations. **c** Prediction bias of mutDDG-SSM

The prediction performance of our mutDDG-SSM model was compared with other 14 previously published models on the S^sym^ test set. As shown in Table 1 and Fig. 2, mutDDG-SSM obtained $PCC=0.64$, $RMSE = 1.28 \;{\text{kcal}}/{\text{mol}}$ and $MAE = 0.90\;{\text{kcal}}/{\text{mol}}$ in the direct mutations, and $PCC = 0.64$, $RMSE = 1.28 \;{\text{kcal}}/{\text{mol}}$ and $MAE = 0.90\;{\text{kcal}}/{\text{mol}}$ in the inverse mutations. For the overall test set containing both direct and inverse mutations, mutDDG-SSM showed $PCC = 0.73$, $RMSE = 1.28 \;{\text{kcal}}/{\text{mol}}$, $MAE = 0.90\;{\text{kcal}}/{\text{mol}}$, respectively. Compared to the previously reported methods, our model achieved better performance. For direct mutations, the PCC value of our model is lower than that of SAAFEC-SEQ [51] and cartesian_ddg (Cartddg) [52], while for inverse mutations, our model outperforms all other models listed in Table 1. The overall prediction accuracy of our model is the best, which is not only significantly superior than the empirical potential-based methods, such as Cartddg, FoldX and SDM [53], but also better than other machine learning (ML)-based models, as shown in Table 1. The $PCC$ value obtained by our model is higher than all the other previously developed models listed in Table 1, and the $RMSE$ and $MAE$ values are lower than all of previous models.

**Table 1:** Comparison of the $\Delta \Delta G$ prediction performance between different methods on the S^sym^ dataset

Method	Overall			Direct			Inverse			Prediction bias	
	PCC	RMSE	MAE	PCC	RMSE	MAE	PCC	RMSE	MAE	*r*^*d−i*^	< δ >
mutDDG-SSM	**0.73**	**1.28**	**0.90**	0.64	1.28	0.90	**0.64**	**1.28**	**0.90**	− **0.99**	**0.00**
KORPM [54]	0.69	1.35	0.97	0.56	1.30	0.94	0.49	1.40	1.00	− 0.88	− 0.06
Cartddg	0.63	3.44	2.64	0.66	3.32	2.66	0.45	3.56	2.63	− 0.41	− 1.56
FoldX	0.54	1.88	1.31	0.64	1.49	1.08	0.38	2.20	1.54	− 0.27	− 0.63
Evo [55]	0.55	1.57	1.13	0.58	1.36	1.00	0.32	1.75	1.26	− 0.58	− 0.18
PoPMuSiC [32]	0.52	1.60	1.17	0.48	1.58	1.16	0.47	1.62	1.18	− 0.77	− 0.03
DDGun3D [56]	0.63	1.45	1.06	0.55	1.43	1.05	0.53	1.47	1.07	− **0.99**	− 0.02
ThermoNet [57]	0.55	1.55	1.11	0.47	1.55	1.11	0.47	1.56	1.12	− 0.96	− 0.01
ACDC-NN [58]	0.69	1.40	1.02	0.61	1.37	1.01	0.59	1.43	1.04	− 0.98	− 0.03
MAESTRO [59]	0.43	1.79	1.29	0.57	1.31	0.91	0.27	2.16	1.66	− 0.33	− 0.62
Dynamut [60]	0.50	1.61	1.15	0.56	1.46	1.04	0.35	1.75	1.26	− 0.57	− 0.13
mCSM [38]	0.40	1.93	1.42	0.61	1.23	0.91	0.14	2.43	1.93	− 0.26	− 0.91
SDM	0.32	1.99	1.51	0.50	1.57	1.22	0.17	2.34	1.80	− 0.43	− 0.55
DUET [61]	0.43	1.84	1.32	0.63	1.22	0.87	0.17	2.30	1.76	− 0.30	− 0.74
SAAFEC-SEQ	0.26	2.08	1.42	**0.73**	**1.05**	**0.73**	− 0.43	2.75	2.11	0.67	− 0.97

The best predicted results among all tested methods on the S^sym^ dataset are shown in bold for each criteria. The units of RMSE, MAE and δ are kcal/mol

Our model also exhibited unbiased performance with high anti-symmetric properties. Table 1 and Fig. 2 show that the prediction accuracy of our model on the inverse mutations is as good as that of the direct mutations. In order to measure the prediction bias, the PCC value ${r}^{d-i}$ between the predictions for direct mutations and those for inverse mutations, along with the $\langle \delta \rangle$ value, was calculated according to Eqs. (6) and (7) described in the Method section. The closer the values of ${r}^{d-i}$ and $\langle \delta \rangle$ are to − 1 and 0, the better the unbiasedness of the model. Table 1 and Fig. 2 show ${r}^{d-i}=-0.99$ and $\langle \delta \rangle =0.00$ for mutDDG-SSM, indicating that our model is unbiased in predicting the $\Delta \Delta G$ values. Table 1 also display that both the ${r}^{d-i}$ and $\langle \delta \rangle$ values of our model are better than all the other previous methods, which demonstrates that the unbiasedness of our model outperforms other methods.

Considering that the naturally-occurring disease-related mutations in proteins are usually SNVs, we then assess the performance of mutDDG-SSM on the S^sym^ dataset by classifying mutations into SNVs and non-SNVs. S^sym^ dataset consists of 214 SNVs and 128 non-SNVs, respectively. The calculation results show the performance of mutDDG-SSM is better than all other models on non-SNVs, as displayed in Table 2. The prediction accuracy of mutDDG-SSM on SNVs is obviously inferior to that of non-SNVs, but it is also superior than most of the other models listed in Table 2. Pandey et al. also revealed that all the models tested in their study work better on non-SNVs than SNVs [35], which is consistent with our results.

**Table 2:** Comparison of the ∆∆G prediction performance between different methods on the S^sym^ dataset by classifying mutations into SNVs and non-SNVs

Method	SNVs			non-SNVs		
	PCC	RMSE	MAE	PCC	RMSE	MAE
mutDDG-SSM	0.55	1.27	**0.90**	**0.84**	**1.32**	**0.95**
KORPM	0.60	**1.22**	0.92	0.77	1.54	1.05
Cartddg	**0.62**	3.08	2.37	0.64	3.97	3.11
FoldX	0.49	1.65	1.20	0.48	1.67	1.18
Evo	0.41	1.43	1.05	0.65	1.78	1.26
PoPMuSiC	0.39	1.40	1.04	0.63	1.89	1.39
DDGun3D	0.41	1.38	0.99	0.43	1.39	0.97
ThermoNet	0.43	1.37	0.99	0.63	1.81	1.32
ACDC-NN	0.51	1.32	0.95	0.51	**1.32**	**0.95**
MAESTRO	0.27	1.61	1.17	0.27	1.61	1.17
Dynamut	0.30	1.48	1.06	0.30	1.48	1.06
mCSM	0.26	1.73	1.30	0.26	1.73	1.30
SDM	0.26	1.72	1.32	0.26	1.72	1.32
DUET	0.32	1.63	1.18	0.32	1.63	1.18
SAAFEC-SEQ	0.21	1.80	1.26	0.31	2.49	1.68

The best predicted results among all tested methods on the S^sym^ dataset by classifying mutations into SNVs and non-SNVs are shown in bold for each criteria. The units of RMSE and MAE are kcal/mol

### Performance in classifying stabilizing and destabilizing mutations

We also explored the performance of mutDDG-SSM in distinguishing the stabilizing and destabilizing mutations in the S^sym^ test set. The mutations with a negative ΔΔG value were defined as stabilizing ones, and those with a positive value were destabilizing ones. The ACC, SEN, SPE, and MCC of our mutDDG-SSM model were computed, and compared with other previously developed methods. The calculation results are displayed in Table 3. mutDDG-SSM is one of the best models in classifying the stabilizing and destabilizing mutations on the overall S^sym^ test set, with $ACC=0.77$ and $MCC=0.54$. Our method not only outperforms the empirical potential-based methods, such as Cartddg, FoldX and SDM, but also is better than most of other previously reported ML-based models, as shown in Table 3. We also compared the performances of the models on SNVs and non-SNVs. Most of the models including mutDDG-SSM perform better on non-SVNs than SVNs, and mutDDG-SSM outperforms most of the methods both on SVNs and non-SVNs.

**Table 3:** Comparison of the performance between different methods in distinguishing the stabilizing and destabilizing mutations on the S^sym^ dataset, as well as on the SNVs and non-SNVs subsets of S^sym^

Method	Overall				SNVs				non-SNVs			
	ACC	SEN	SPE	MCC	ACC	SEN	SPE	MCC	ACC	SEN	SPE	MCC
mutDDG-SSM	0.77	0.77	0.77	0.54	**0.75**	**0.76**	0.74	**0.50**	0.82	0.81	0.83	0.65
KORPM	**0.78**	**0.78**	0.78	**0.56**	**0.75**	0.75	0.75	**0.50**	**0.84**	**0.83**	0.84	**0.67**
Cartddg	0.73	0.58	0.87	0.48	0.73	0.58	0.87	0.47	0.74	0.59	0.88	0.49
FoldX	0.66	0.55	0.77	0.33	0.64	0.55	0.74	0.29	0.69	0.56	0.82	0.39
Evo	0.65	0.61	0.69	0.31	0.63	0.59	0.67	0.25	0.70	0.65	0.74	0.40
PoPMuSiC	0.69	0.68	0.71	0.38	0.65	0.63	0.67	0.31	0.75	0.75	0.76	0.51
DDGun3D	0.67	0.67	0.67	0.34	0.66	0.65	0.67	0.32	0.70	0.70	0.69	0.39
ThermoNet	0.69	0.67	0.71	0.38	0.68	0.66	0.71	0.36	0.70	0.69	0.71	0.40
ACDC-NN	0.69	0.7	0.69	0.39	0.64	0.64	0.64	0.29	0.78	0.79	0.77	0.56
MAESTRO	0.6	0.24	0.93	0.24	0.57	0.19	0.94	0.18	0.64	0.33	0.93	0.33
Dynamut	0.68	0.68	0.68	0.35	0.66	0.68	0.64	0.32	0.71	0.67	0.74	0.41
mCSM	0.55	0.13	**0.96**	0.15	0.54	0.10	**0.97**	0.14	0.57	0.19	0.93	0.18
SDM	0.62	0.46	0.76	0.24	0.62	0.47	0.77	0.25	0.60	0.44	0.75	0.20
DUET	0.63	0.35	0.89	0.29	0.62	0.31	0.91	0.28	0.64	0.42	0.86	0.31
SAAFEC-SEQ	0.56	0.16	0.93	0.15	0.56	0.19	0.92	0.15	0.55	0.13	**0.95**	0.13

The best predicted results among all tested methods in distinguishing the stabilizing and destabilizing mutations on the S^sym^ dataset are shown in bold for each criteria

### Testing results on other different datasets

**Fig. 3:**
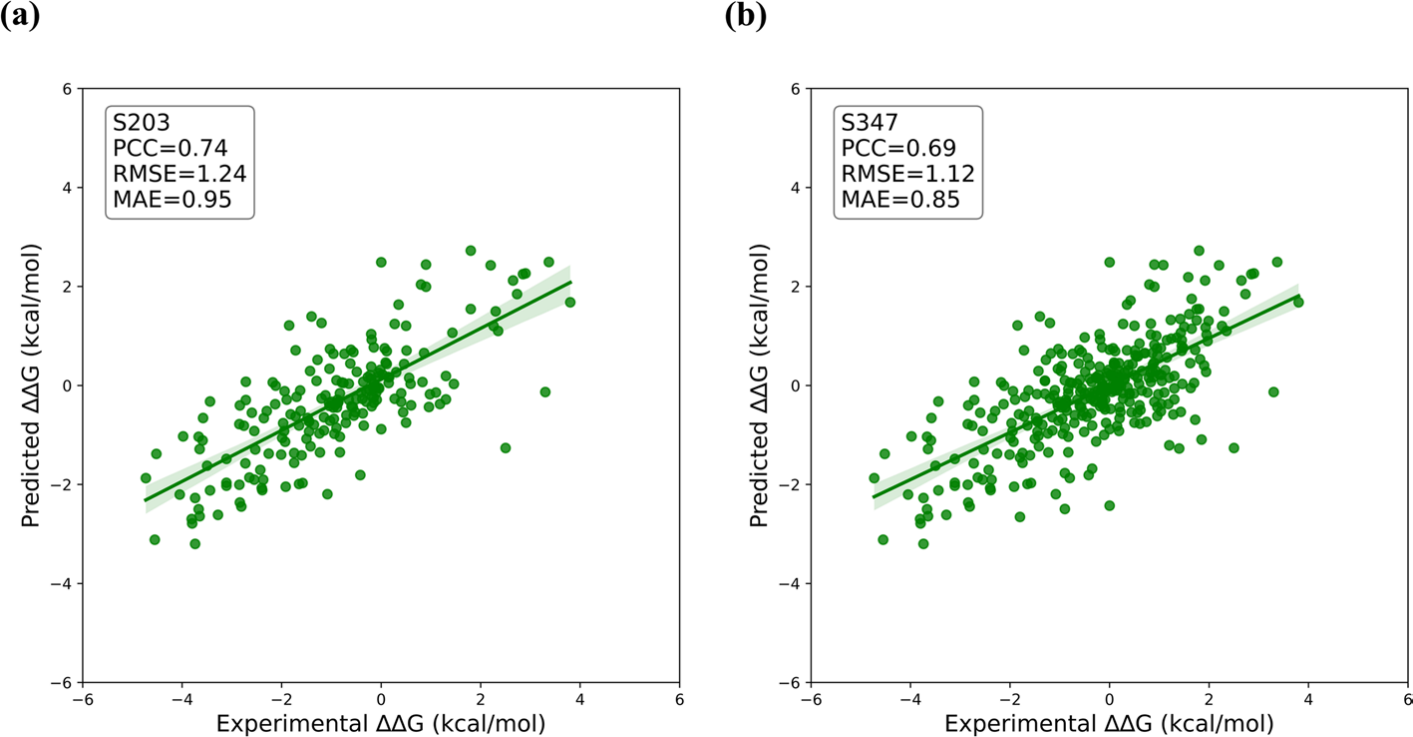
The performance of mutDDG-SSM in predicting $\Delta \Delta G$ on the S203 and S347 datasets. **a** The performance of mutDDG-SSM on S203. **b** The performance of mutDDG-SSM on S347

We also tested mutDDG-SSM using two other independent datasets, i.e., S203 and S347, which are derived from S350 and S611, respectively, after removing the mutations shared with the training dataset Q3213. The PCC, RMSE and MAE values between the mutDDG-SSM predicted and the experimental $\Delta \Delta G$ were calculated, and compared with the results obtained by other previously developed methods. The computed results show that the PCC value of mutDDG-SSM on the S203 dataset is 0.74, as displayed in Table 4 and Fig. 3, which is lower than the value of SAAFEC-SEQ but higher than all the other models listed in Table 4. For the S347 dataset, the PCC values obtained by mutDDG-SSM reached 0.69, as shown in Table 5 and Fig. 3, which is higher than all the statistical potential-based methods (for example SDM and FoldX) and other ML-based methods. We then separated mutations into SNVs and non-SNVs. S203 dataset comprises 119 SNVs and 84 non-SNVs, and S347 consists of 208 SNVs and 139 non-SNVs. For SVNs and non-SVNs, the similar results were obtained, where the performance of mutDDG-SSM is better than all the other models except SAAFEC-SEQ, as shown in Tables 4 and 5.

**Table 4:** Comparison of the $\Delta \Delta G$ prediction performance between different methods on the S203 dataset, as well as on the SNVs and non-SNVs subsets of S203

Method	Direct			SNVs			non-SNVs		
	PCC	RMSE	MAE	PCC	RMSE	MAE	PCC	RMSE	MAE
mutDDG-SSM	0.74	1.24	0.95	0.67	1.26	0.94	0.80	1.21	0.97
Dynamut	0.47	1.77	1.39	0.51	1.68	1.33	0.44	1.88	1.48
mCSM	0.69	1.20	0.93	0.65	1.16	0.87	0.73	1.26	1.02
DUET	0.70	1.18	0.91	0.66	1.15	0.86	0.74	1.22	0.99
SDM	0.55	1.86	1.43	0.54	1.78	1.34	0.56	1.98	1.56
I-Mutant2.0 [62]	0.59	1.33	1.01	0.51	1.31	0.94	0.67	1.36	1.12
MAESTRO	0.66	1.75	1.39	0.61	1.69	1.31	0.72	1.83	1.50
ThermoNet	0.62	1.48	1.15	0.59	1.45	1.13	0.65	1.52	1.18
FoldX	0.50	2.18	1.26	0.54	1.64	1.03	0.49	2.77	1.58
SAAFEC-SEQ	**0.90**	**0.82**	**0.60**	**0.90**	**0.73**	**0.52**	**0.90**	**0.92**	**0.71**

The best predicted results among all tested methods on the S203 dataset are shown in bold for each criteria. The units of RMSE and MAE are kcal/mol

**Table 5:** Comparison of the $\Delta \Delta G$ prediction performance between different methods on the S347 dataset, as well as on the SNVs and non-SNVs subsets of S347

Method	Overall			SNVs			Non-SNVs		
	PCC	RMSE	MAE	PCC	RMSE	MAE	PCC	RMSE	MAE
mutDDG-SSM	**0.69**	**1.12**	**0.85**	**0.65**	**1.12**	**0.83**	**0.74**	**1.13**	**0.89**
Dynamut	0.34	1.52	1.17	0.39	1.41	1.08	0.29	1.67	1.29
mCSM	0.51	1.48	1.17	0.46	1.42	1.13	0.56	1.55	1.23
DUET	0.52	1.46	1.14	0.47	1.40	1.09	0.58	1.54	1.21
SDM	0.43	1.94	1.49	0.41	1.84	1.36	0.45	2.08	1.67
I-Mutant2.0	0.38	1.53	1.20	0.31	1.47	1.13	0.45	1.62	1.30
MAESTRO	0.48	1.66	1.25	0.42	1.43	1.09	0.54	1.59	1.28
ThermoNet	0.56	1.30	1.00	0.54	0.96	1.25	0.58	1.38	1.06
FoldX	0.55	1.87	1.15	0.48	1.61	1.14	0.41	2.58	1.67
SAAFEC-SEQ	0.68	1.24	0.93	**0.65**	1.23	0.91	0.72	1.27	0.98

The best predicted results among all tested methods on the S347 dataset are shown in bold for each criteria. The units of RMSE and MAE are kcal/mol

Comparison between Table 4 with Table 5 shows that the prediction performances for all the methods, except FoldX, on the S347 dataset are inferior to those on the S203 dataset. The result indicates that these methods performed weaker on the inverse mutations than on the direct mutations, and therefore including inverse mutations in the S347 dataset distinctly reduced the prediction accuracy. However, our method mutDDG-SSM performed well both on the S203 and S347 datasets and outperformed FoldX, demonstrating the unbiasedness and accuracy of our method.

Furthermore, the performance of mutDDG-SSM was also tested on the S254 and S615 datasets. The calculation results demonstrate that the PCC values between the predicted and experimental data are 0.46 and 0.49 on these two datasets, respectively. In addition, mutDDG-SSM also exhibited unbiased performance, with the ${r}^{d-i}$ values to be − 0.99 and − 1.00 for S254 and S615, respectively, as shown in Supplementary Fig. 1, 2 and Supplementary Tables 6, 7.

## Case studies

**Fig. 4:**
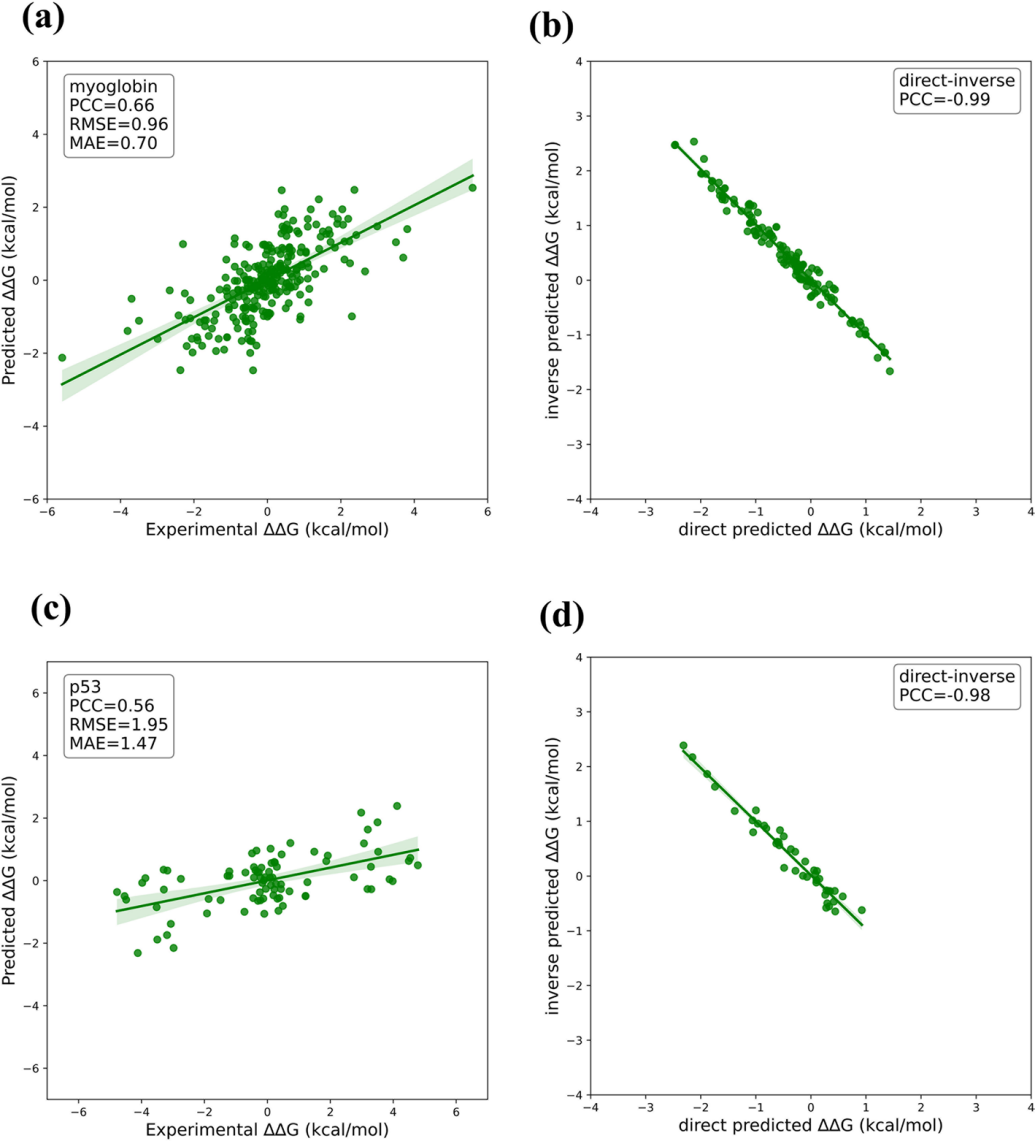
The performance of mutDDG-SSM in predicting ∆∆G on two cases, i.e., myoglobin and p53. **a** The performance of mutDDG-SSM on myoglobin. **b** Prediction bias of mutDDG-SSM on myoglobin. **c** The performance of mutDDG-SSM on p53. **d** Prediction bias of mutDDG-SSM on p53

To further illustrate the prediction performance of mutDDG-SSM, two protein systems, i.e., myoglobin and p53, were investigated as case studies. As shown in Fig. 4, for myoglobin, the PCC between the predicted and experimental $\Delta \Delta G$ values reached 0.66, with $RMSE = 0.96 \;{\text{kcal}}/{\text{mol}}$ and $MAE = 0.70 \;{\text{kcal}}/{\text{mol}}$. As to p53, mutDDG-SSM obtained a PCC value of 0.56, the $RMSE$ and $MAE$ values are $1.95 \;{\text{kcal}}/{\text{mol}}$ and $1.47 \;{\text{kcal}}/{\text{mol}}$, respectively. We also assessed the prediction bias of the mutDDG-SSM predictor on these two protein systems. The ${r}^{d-i}$ values for myoglobin and p53 arrived at − 0.99 and − 0.98, respectively, indicating that mutDDG-SSM is an unbiased predictor with good antisymmetric properties.

It should be noted that due to the unique conformations of alanine and glycine, we refrained from perturbing their side chains in the self-supervised training stage of mutDDG-SSM. However, our model also performed well in predicting protein stability changes for the mutations associated with these two residues. The PCC values for the subsets involving alanine and glycine are closely comparable to that of the entire datasets, as shown in Supplementary Table 8. In the second part of our model, XGBoost was employed as the $\Delta \Delta G$ value predictor. To illustrate the superiority of XGBoost, several other ML models were also tested in our framework. Our results demonstrated that XGBoost achieved the best performance against other ML models, as shown in Supplementary Table 9.

## Discussion

Effectively predicting the changes in protein stability caused by residue mutations is crucial and valuable for the understanding of protein structure–function relationship and the application in protein engineering. Because the available experimental data that can be used for the model training is limited and unbalanced with much more destabilizing mutations than stabilizing ones, a large portion of the existing ML-based models are considered to be overfitting and biased. In the present study, we presented mutDDG-SSM, a ML-based framework to predict the changes of protein stability upon a single-point residue mutation. Tests on several widely-used independent datasets and two protein cases demonstrated that mutDDG-SSM achieved high performance in predicting the mutation-induced ΔΔG values, which outperformed not only the empirical potential-based methods, but also many of other previously reported ML-based models. mutDDG-SSM also obtained one of the best performances in distinguishing stabilizing and destabilizing mutations. In addition, mutDDG-SSM exhibited unbiased prediction performance with high anti-symmetric properties. When separating mutations into SNVs and non-SNVs, the performance of mutDDG-SSM is superior than most of other ML-based models both on SNVs and non-SNVs, although the prediction accuracy for SNVs is lower than that of non-SNVs. The robust and unbiased prediction performance may enable our model to serve as a valuable tool for protein engineering and drug design.

In mutDDG-SSM, a protein structure was simplified as a graph with nodes and edges, in which the exact interactions between protein atoms, e.g. hydrogen bonds, were not explicitly incorporated into the model. However, through a self-supervised training strategy, the interactions of a residue with its surrounding neighbors can be extracted implicitly. mutDDG-SSM also has some limitations. In this study, our model was trained and tested using monomeric proteins, and the pH of protein solution was not explicitly considered. We will further extend the model to multimeric proteins and the proteins with multiple mutations, and incorporate more biological information into the model in the following studies.

### Supplementary Information

Supplementary file1

Supplementary file2

## Data Availability

The data and code can be downloaded from the following website: https://github.com/SJGLAB/mutDDG_SSM.git.
