# Supplementary material for: Prediction of mutation-induced protein stability changes based on the geometric representations learned by a self-supervised method
Source: BMC Bioinformatics. 2024 Aug 28;25:282. doi: 10.1186/s12859-024-05876-6 (PMC11360314; doi:10.1186/s12859-024-05876-6)
Supplement: Supplementary file 2 — Supplementary file2 [file 12859_2024_5876_MOESM2_ESM.docx]

**Supplementary Material**

Supplementary Table 2. The S203 dataset.

| PDB_code | chain | mutation | ΔΔG(kcal/mol) | PDB_code | chain | mutation | ΔΔG(kcal/mol) |
| --- | --- | --- | --- | --- | --- | --- | --- |
| 1AKY | A | Q48E | -0.96 | 1HMK | A | Y103F | -2.13 |
| 1AON | U | I48W | -0.2 | 1HMK | A | L110A | -0.35 |
| 1CEY | A | D12A | 2.5 | 1HMS | A | F4S | -3.67 |
| 1CEY | A | D57A | 3.3 | 1HMS | A | F16S | -3.98 |
| 1CSE | I | V54A | -1.58 | 1HMS | A | T40E | -2.4 |
| 1CUN | A | Q115G | -1.15 | 1HMS | A | F57S | -2.43 |
| 1CUN | A | A126G | -1.65 | 1HMS | A | L66G | -3.67 |
| 1CUN | A | I128A | -1.65 | 1HMS | A | R106T | -2.84 |
| 1CUN | A | I128V | -2.85 | 1IET | A | D60R | 0.14 |
| 1CUN | A | K152A | -0.15 | 1IFC | A | W6Y | -0.87 |
| 1CUN | A | K152G | -1.45 | 1IFC | A | V60C | -0.07 |
| 1CUN | A | A156G | -1.45 | 1IFC | A | V60N | -0.83 |
| 1CUN | A | F157L | -1.85 | 1IFC | A | L64G | -2.26 |
| 1CUN | A | A173G | -1.95 | 1IFC | A | G65A | -0.94 |
| 1CUN | A | A191G | -1.35 | 1IFC | A | F68A | -0.42 |
| 1CUN | A | M193A | -2.65 | 1IFC | A | F93A | -2.37 |
| 1CUN | A | L196A | -4.55 | 1IHB | A | F82Q | -0.37 |
| 1CUN | A | S201A | 0.15 | 1JIW | I | D10A | -0.7 |
| 1CUN | A | S201G | -0.95 | 1JIW | I | W15F | -2.3 |
| 1CUN | A | L203A | -4.05 | 1K9Q | A | L30Y | 0.27 |
| 1CUN | A | A212G | -1.45 | 1LNI | A | T16V | 0.3 |
| 1CUN | A | L214A | -3.65 | 1LNI | A | Y30F | 0.4 |
| 1E65 | A | I7S | -3.44 | 1LNI | A | V43T | -0.5 |
| 1E65 | A | I20T | -2.39 | 1LNI | A | Y55F | -0.6 |
| 1E65 | A | V31T | -1.08 | 1LNI | A | T56V | -1.9 |
| 1E65 | A | L50V | -0.36 | 1LNI | A | D79F | 2.73 |
| 1E65 | A | V60G | -3.11 | 1LNI | A | D79I | 2.85 |
| 1E65 | A | A82G | -3.11 | 1LNI | A | D79K | 2.35 |
| 1E65 | A | V95T | 0.96 | 1LNI | A | D79L | 2.65 |
| 1E65 | A | H117G | -2.18 | 1LNI | A | D79N | 1.46 |
| 1EY0 | A | L7V | -1.15 | 1LNI | A | D79Y | 2.9 |
| 1EY0 | A | K9F | -1.03 | 1LNI | A | Y80F | -1.5 |
| 1EY0 | A | T13C | -1.2 | 1LNI | A | H85Q | 0 |
| 1EY0 | A | L14V | -1.63 | 1LNI | A | Q94K | 0.56 |
| 1EY0 | A | I15M | -0.15 | 1MGR | A | Y54F | -2.6 |
| 1EY0 | A | D19F | -1.28 | 1MGR | A | Y84F | -1 |
| 1EY0 | A | D21K | 1.1 | 1MJC | A | S52W | -0.2 |
| 1EY0 | A | T22I | -0.61 | 1P2P | A | H48K | -2.12 |
| 1EY0 | A | V23I | 0.03 | 1QLP | A | A31L | 0.9 |
| 1EY0 | A | V23L | -0.02 | 1QLP | A | V55I | -0.2 |
| 1EY0 | A | K24F | -0.4 | 1QLP | A | Y160W | 1.18 |
| 1EY0 | A | Y27C | -2.72 | 1QLP | A | A183I | 1.8 |
| 1EY0 | A | G29V | -3.11 | 1QLP | A | A183V | 3.8 |
| 1EY0 | A | T33C | -1.04 | 1QLP | A | W238F | 0.98 |
| 1EY0 | A | L36V | -3.58 | 1QLP | A | A248F | 1.8 |
| 1EY0 | A | L37I | -1.82 | 1QLP | A | A248I | 2.2 |
| 1EY0 | A | V39I | 0.11 | 1QLP | A | A248L | 0.35 |
| 1EY0 | A | V39L | -0.9 | 1QLP | A | A248V | 2.3 |
| 1EY0 | A | T41I | 0.86 | 1QLP | A | A284I | 0 |
| 1EY0 | A | T44C | -0.04 | 1QLP | A | A284V | 0.8 |
| 1EY0 | A | V51L | -0.1 | 1QLP | A | V321I | 0.6 |
| 1EY0 | A | Y54F | -0.38 | 1QLP | A | V364L | -0.3 |
| 1EY0 | A | G55V | -1.48 | 1RG8 | A | C16S | -2.81 |
| 1EY0 | A | K63Q | -0.89 | 1RG8 | A | L44F | 0.59 |
| 1EY0 | A | M65F | -1.62 | 1RG8 | A | N106G | 0.16 |
| 1EY0 | A | M65I | -1.43 | 1RG8 | A | V109I | -0.05 |
| 1EY0 | A | V66I | -0.76 | 1RN1 | C | V16T | -3.65 |
| 1EY0 | A | K70E | -0.3 | 1RN1 | C | G23A | -1.2 |
| 1EY0 | A | I72L | -0.23 | 1RN1 | C | D49A | 0.5 |
| 1EY0 | A | V74L | -1.12 | 1RN1 | C | V78S | -4.73 |
| 1EY0 | A | D77K | -3.28 | 1RN1 | C | V78T | -3.59 |
| 1EY0 | A | K78Q | -0.15 | 1SHF | A | I111A | -2.84 |
| 1EY0 | A | T82C | -0.19 | 1SHF | A | I111L | -0.71 |
| 1EY0 | A | T82I | 0.51 | 1SHF | A | V138M | -0.52 |
| 1EY0 | A | K84Q | -0.15 | 1TTQ | A | F22L | -1.05 |
| 1EY0 | A | G86F | -1.99 | 1TTQ | A | F22V | -3.44 |
| 1EY0 | A | L89I | -1.04 | 1YYJ | A | L3A | -1.6 |
| 1EY0 | A | I92M | -1.75 | 1YYJ | A | A20G | -1.97 |
| 1EY0 | A | G96F | -2.55 | 1YYJ | A | F61A | -4.52 |
| 1EY0 | A | G96V | -3.74 | 1YYJ | A | F65A | -1.92 |
| 1EY0 | A | V104I | 0.27 | 1YYJ | A | Y105A | -2.36 |
| 1EY0 | A | R105C | -2.55 | 1ZG4 | A | W290F | -0.83 |
| 1EY0 | A | L108V | -3.81 | 2A01 | A | L141R | -0.65 |
| 1EY0 | A | V111I | -0.74 | 2A36 | A | T22A | 0.4 |
| 1EY0 | A | V111L | -0.88 | 2A36 | A | T22F | 1.3 |
| 1EY0 | A | V114I | -0.15 | 2A36 | A | T22L | 0.5 |
| 1EY0 | A | H124E | 0.46 | 2A36 | A | T22N | 1.3 |
| 1EY0 | A | L125I | -0.96 | 2DRI | A | V50E | -3.5 |
| 1EY0 | A | L137V | -1.42 | 2NVH | A | R4Q | -0.57 |
| 1EY0 | A | I139L | -0.09 | 2NVH | A | N7D | 0.09 |
| 1FNA | A | I34V | -0.11 | 2NVH | A | C8S | -3.74 |
| 1FNA | A | V50A | -2.85 | 2RN2 | A | H62A | 0.44 |
| 1G4I | A | F22I | 1.43 | 2TRX | A | D26I | 3.37 |
| 1G4I | A | F22Y | 0.83 | 2TRX | A | T66L | -1.03 |
| 1G4I | A | H48A | -1.93 | 3GLY | A | G139A | -1.4 |
| 1G4I | A | H48Q | -0.49 | 3GLY | A | G383A | 0.11 |
| 1G4I | A | F106A | -1.23 | 3SIL | A | A53L | 0.9 |
| 1HFZ | A | H32Y | 0.07 | 5DFR | A | I2V | -0.55 |
| 1HFZ | A | I59W | -0.93 | 5DFR | A | W30M | -1.94 |
| 1HFZ | A | H107W | -1.72 | 5DFR | A | V40H | -2.76 |
| 1HFZ | A | H107Y | -0.19 | 5DFR | A | N59T | -0.05 |
| 1HFZ | A | L110E | -0.19 | 5DFR | A | N59W | -0.79 |
| 1HFZ | A | K114E | -0.65 | 5DFR | A | G67S | -0.27 |
| 1HMK | A | V8A | -0.83 | 5DFR | A | G67T | -0.62 |
| 1HMK | A | L12A | -2.73 | 5DFR | A | G95A | -0.9 |
| 1HMK | A | V27A | -1.24 | 5DFR | A | G121C | -0.22 |
| 1HMK | A | T29V | 2.26 | 5DFR | A | G121H | -0.56 |
| 1HMK | A | I55V | -2.72 | 5DFR | A | I155T | -2.53 |
| 1HMK | A | W60A | -2.01 | 5PTI | A | A16T | -1.7 |
| 1HMK | A | I89V | -0.86 | 5PTI | A | A16V | -1.3 |
| 1HMK | A | I95V | -1.72 | 5PTI | A | Y35D | -3.8 |
| 1HMK | A | L96A | -1.75 |  |  |  |  |

Supplementary Table 3. The S347 dataset.

| PDB_code | chain | mutation | mutation_type | ΔΔG(kcal/mol) |
| --- | --- | --- | --- | --- |
| 1AKY | A | Q48E | Direct mutation | -0.96 |
| 1AON | U | I48W | Direct mutation | -0.2 |
| 1CEY | A | D12A | Direct mutation | 2.5 |
| 1CEY | A | D57A | Direct mutation | 3.3 |
| 1CSE | I | V54A | Direct mutation | -1.58 |
| 1CUN | A | Q115G | Direct mutation | -1.15 |
| 1CUN | A | A126G | Direct mutation | -1.65 |
| 1CUN | A | I128A | Direct mutation | -1.65 |
| 1CUN | A | I128V | Direct mutation | -2.85 |
| 1CUN | A | K152A | Direct mutation | -0.15 |
| 1CUN | A | K152G | Direct mutation | -1.45 |
| 1CUN | A | A156G | Direct mutation | -1.45 |
| 1CUN | A | F157L | Direct mutation | -1.85 |
| 1CUN | A | A173G | Direct mutation | -1.95 |
| 1CUN | A | A191G | Direct mutation | -1.35 |
| 1CUN | A | M193A | Direct mutation | -2.65 |
| 1CUN | A | L196A | Direct mutation | -4.55 |
| 1CUN | A | S201A | Direct mutation | 0.15 |
| 1CUN | A | S201G | Direct mutation | -0.95 |
| 1CUN | A | L203A | Direct mutation | -4.05 |
| 1CUN | A | A212G | Direct mutation | -1.45 |
| 1CUN | A | L214A | Direct mutation | -3.65 |
| 1E65 | A | I7S | Direct mutation | -3.44 |
| 1E65 | A | I20T | Direct mutation | -2.39 |
| 1E65 | A | V31T | Direct mutation | -1.08 |
| 1E65 | A | L50V | Direct mutation | -0.36 |
| 1E65 | A | V60G | Direct mutation | -3.11 |
| 1E65 | A | A82G | Direct mutation | -3.11 |
| 1E65 | A | V95T | Direct mutation | 0.96 |
| 1E65 | A | H117G | Direct mutation | -2.18 |
| 1EY0 | A | L7V | Direct mutation | -1.15 |
| 1EY0 | A | K9F | Direct mutation | -1.03 |
| 1EY0 | A | T13C | Direct mutation | -1.2 |
| 1EY0 | A | L14V | Direct mutation | -1.63 |
| 1EY0 | A | I15M | Direct mutation | -0.15 |
| 1EY0 | A | D19F | Direct mutation | -1.28 |
| 1EY0 | A | D21K | Direct mutation | 1.1 |
| 1EY0 | A | T22I | Direct mutation | -0.61 |
| 1EY0 | A | V23I | Direct mutation | 0.03 |
| 1EY0 | A | V23L | Direct mutation | -0.02 |
| 1EY0 | A | K24F | Direct mutation | -0.4 |
| 1EY0 | A | Y27C | Direct mutation | -2.72 |
| 1EY0 | A | G29V | Direct mutation | -3.11 |
| 1EY0 | A | T33C | Direct mutation | -1.04 |
| 1EY0 | A | L36V | Direct mutation | -3.58 |
| 1EY0 | A | L37I | Direct mutation | -1.82 |
| 1EY0 | A | V39I | Direct mutation | 0.11 |
| 1EY0 | A | V39L | Direct mutation | -0.9 |
| 1EY0 | A | T41I | Direct mutation | 0.86 |
| 1EY0 | A | T44C | Direct mutation | -0.04 |
| 1EY0 | A | V51L | Direct mutation | -0.1 |
| 1EY0 | A | Y54F | Direct mutation | -0.38 |
| 1EY0 | A | G55V | Direct mutation | -1.48 |
| 1EY0 | A | K63Q | Direct mutation | -0.89 |
| 1EY0 | A | M65F | Direct mutation | -1.62 |
| 1EY0 | A | M65I | Direct mutation | -1.43 |
| 1EY0 | A | V66I | Direct mutation | -0.76 |
| 1EY0 | A | K70E | Direct mutation | -0.3 |
| 1EY0 | A | I72L | Direct mutation | -0.23 |
| 1EY0 | A | V74L | Direct mutation | -1.12 |
| 1EY0 | A | D77K | Direct mutation | -3.28 |
| 1EY0 | A | K78Q | Direct mutation | -0.15 |
| 1EY0 | A | T82C | Direct mutation | -0.19 |
| 1EY0 | A | T82I | Direct mutation | 0.51 |
| 1EY0 | A | K84Q | Direct mutation | -0.15 |
| 1EY0 | A | G86F | Direct mutation | -1.99 |
| 1EY0 | A | L89I | Direct mutation | -1.04 |
| 1EY0 | A | I92M | Direct mutation | -1.75 |
| 1EY0 | A | G96F | Direct mutation | -2.55 |
| 1EY0 | A | G96V | Direct mutation | -3.74 |
| 1EY0 | A | V104I | Direct mutation | 0.27 |
| 1EY0 | A | R105C | Direct mutation | -2.55 |
| 1EY0 | A | L108V | Direct mutation | -3.81 |
| 1EY0 | A | V111I | Direct mutation | -0.74 |
| 1EY0 | A | V111L | Direct mutation | -0.88 |
| 1EY0 | A | V114I | Direct mutation | -0.15 |
| 1EY0 | A | H124E | Direct mutation | 0.46 |
| 1EY0 | A | L125I | Direct mutation | -0.96 |
| 1EY0 | A | L137V | Direct mutation | -1.42 |
| 1EY0 | A | I139L | Direct mutation | -0.09 |
| 1FNA | A | I34V | Direct mutation | -0.11 |
| 1FNA | A | V50A | Direct mutation | -2.85 |
| 1G4I | A | F22I | Direct mutation | 1.43 |
| 1G4I | A | F22Y | Direct mutation | 0.83 |
| 1G4I | A | H48A | Direct mutation | -1.93 |
| 1G4I | A | H48Q | Direct mutation | -0.49 |
| 1G4I | A | F106A | Direct mutation | -1.23 |
| 1HFZ | A | H32Y | Direct mutation | 0.07 |
| 1HFZ | A | I59W | Direct mutation | -0.93 |
| 1HFZ | A | H107W | Direct mutation | -1.72 |
| 1HFZ | A | H107Y | Direct mutation | -0.19 |
| 1HFZ | A | L110E | Direct mutation | -0.19 |
| 1HFZ | A | K114E | Direct mutation | -0.65 |
| 1HMK | A | V8A | Direct mutation | -0.83 |
| 1HMK | A | L12A | Direct mutation | -2.73 |
| 1HMK | A | V27A | Direct mutation | -1.24 |
| 1HMK | A | T29V | Direct mutation | 2.26 |
| 1HMK | A | I55V | Direct mutation | -2.72 |
| 1HMK | A | W60A | Direct mutation | -2.01 |
| 1HMK | A | I89V | Direct mutation | -0.86 |
| 1HMK | A | I95V | Direct mutation | -1.72 |
| 1HMK | A | L96A | Direct mutation | -1.75 |
| 1HMK | A | Y103F | Direct mutation | -2.13 |
| 1HMK | A | L110A | Direct mutation | -0.35 |
| 1HMS | A | F4S | Direct mutation | -3.67 |
| 1HMS | A | F16S | Direct mutation | -3.98 |
| 1HMS | A | T40E | Direct mutation | -2.4 |
| 1HMS | A | F57S | Direct mutation | -2.43 |
| 1HMS | A | L66G | Direct mutation | -3.67 |
| 1HMS | A | R106T | Direct mutation | -2.84 |
| 1IET | A | D60R | Direct mutation | 0.14 |
| 1IFC | A | W6Y | Direct mutation | -0.87 |
| 1IFC | A | V60C | Direct mutation | -0.07 |
| 1IFC | A | V60N | Direct mutation | -0.83 |
| 1IFC | A | L64G | Direct mutation | -2.26 |
| 1IFC | A | G65A | Direct mutation | -0.94 |
| 1IFC | A | F68A | Direct mutation | -0.42 |
| 1IFC | A | F93A | Direct mutation | -2.37 |
| 1IHB | A | F82Q | Direct mutation | -0.37 |
| 1JIW | I | D10A | Direct mutation | -0.7 |
| 1JIW | I | W15F | Direct mutation | -2.3 |
| 1K9Q | A | L30Y | Direct mutation | 0.27 |
| 1LNI | A | T16V | Direct mutation | 0.3 |
| 1LNI | A | Y30F | Direct mutation | 0.4 |
| 1LNI | A | V43T | Direct mutation | -0.5 |
| 1LNI | A | Y55F | Direct mutation | -0.6 |
| 1LNI | A | T56V | Direct mutation | -1.9 |
| 1LNI | A | D79F | Direct mutation | 2.73 |
| 1LNI | A | D79I | Direct mutation | 2.85 |
| 1LNI | A | D79K | Direct mutation | 2.35 |
| 1LNI | A | D79L | Direct mutation | 2.65 |
| 1LNI | A | D79N | Direct mutation | 1.46 |
| 1LNI | A | D79Y | Direct mutation | 2.9 |
| 1LNI | A | Y80F | Direct mutation | -1.5 |
| 1LNI | A | H85Q | Direct mutation | 0 |
| 1LNI | A | Q94K | Direct mutation | 0.56 |
| 1MGR | A | Y54F | Direct mutation | -2.6 |
| 1MGR | A | Y84F | Direct mutation | -1 |
| 1MJC | A | S52W | Direct mutation | -0.2 |
| 1P2P | A | H48K | Direct mutation | -2.12 |
| 1QLP | A | A31L | Direct mutation | 0.9 |
| 1QLP | A | V55I | Direct mutation | -0.2 |
| 1QLP | A | Y160W | Direct mutation | 1.18 |
| 1QLP | A | A183I | Direct mutation | 1.8 |
| 1QLP | A | A183V | Direct mutation | 3.8 |
| 1QLP | A | W238F | Direct mutation | 0.98 |
| 1QLP | A | A248F | Direct mutation | 1.8 |
| 1QLP | A | A248I | Direct mutation | 2.2 |
| 1QLP | A | A248L | Direct mutation | 0.35 |
| 1QLP | A | A248V | Direct mutation | 2.3 |
| 1QLP | A | A284I | Direct mutation | 0 |
| 1QLP | A | A284V | Direct mutation | 0.8 |
| 1QLP | A | V321I | Direct mutation | 0.6 |
| 1QLP | A | V364L | Direct mutation | -0.3 |
| 1RG8 | A | C16S | Direct mutation | -2.81 |
| 1RG8 | A | L44F | Direct mutation | 0.59 |
| 1RG8 | A | N106G | Direct mutation | 0.16 |
| 1RG8 | A | V109I | Direct mutation | -0.05 |
| 1RN1 | C | V16T | Direct mutation | -3.65 |
| 1RN1 | C | G23A | Direct mutation | -1.2 |
| 1RN1 | C | D49A | Direct mutation | 0.5 |
| 1RN1 | C | V78S | Direct mutation | -4.73 |
| 1RN1 | C | V78T | Direct mutation | -3.59 |
| 1SHF | A | I111A | Direct mutation | -2.84 |
| 1SHF | A | I111L | Direct mutation | -0.71 |
| 1SHF | A | V138M | Direct mutation | -0.52 |
| 1TTQ | A | F22L | Direct mutation | -1.05 |
| 1TTQ | A | F22V | Direct mutation | -3.44 |
| 1YYJ | A | L3A | Direct mutation | -1.6 |
| 1YYJ | A | A20G | Direct mutation | -1.97 |
| 1YYJ | A | F61A | Direct mutation | -4.52 |
| 1YYJ | A | F65A | Direct mutation | -1.92 |
| 1YYJ | A | Y105A | Direct mutation | -2.36 |
| 1ZG4 | A | W290F | Direct mutation | -0.83 |
| 2A01 | A | L141R | Direct mutation | -0.65 |
| 2A36 | A | T22A | Direct mutation | 0.4 |
| 2A36 | A | T22F | Direct mutation | 1.3 |
| 2A36 | A | T22L | Direct mutation | 0.5 |
| 2A36 | A | T22N | Direct mutation | 1.3 |
| 2DRI | A | V50E | Direct mutation | -3.5 |
| 2NVH | A | R4Q | Direct mutation | -0.57 |
| 2NVH | A | N7D | Direct mutation | 0.09 |
| 2NVH | A | C8S | Direct mutation | -3.74 |
| 2RN2 | A | H62A | Direct mutation | 0.44 |
| 2TRX | A | D26I | Direct mutation | 3.37 |
| 2TRX | A | T66L | Direct mutation | -1.03 |
| 3GLY | A | G139A | Direct mutation | -1.4 |
| 3GLY | A | G383A | Direct mutation | 0.11 |
| 3SIL | A | A53L | Direct mutation | 0.9 |
| 5DFR | A | I2V | Direct mutation | -0.55 |
| 5DFR | A | W30M | Direct mutation | -1.94 |
| 5DFR | A | V40H | Direct mutation | -2.76 |
| 5DFR | A | N59T | Direct mutation | -0.05 |
| 5DFR | A | N59W | Direct mutation | -0.79 |
| 5DFR | A | G67S | Direct mutation | -0.27 |
| 5DFR | A | G67T | Direct mutation | -0.62 |
| 5DFR | A | G95A | Direct mutation | -0.9 |
| 5DFR | A | G121C | Direct mutation | -0.22 |
| 5DFR | A | G121H | Direct mutation | -0.56 |
| 5DFR | A | I155T | Direct mutation | -2.53 |
| 5PTI | A | A16T | Direct mutation | -1.7 |
| 5PTI | A | A16V | Direct mutation | -1.3 |
| 5PTI | A | Y35D | Direct mutation | -3.8 |
| 1AKY | A | E48Q | Inverse mutation | 0.96 |
| 1AON | U | W48I | Inverse mutation | 0.2 |
| 1CSE | I | A54V | Inverse mutation | 1.58 |
| 1CUN | A | G115Q | Inverse mutation | 1.15 |
| 1CUN | A | G126A | Inverse mutation | 1.65 |
| 1CUN | A | A128I | Inverse mutation | 1.65 |
| 1CUN | A | A152K | Inverse mutation | 0.15 |
| 1CUN | A | G152K | Inverse mutation | 1.45 |
| 1CUN | A | G156A | Inverse mutation | 1.45 |
| 1CUN | A | L157F | Inverse mutation | 1.85 |
| 1CUN | A | G173A | Inverse mutation | 1.95 |
| 1CUN | A | G191A | Inverse mutation | 1.35 |
| 1CUN | A | A201S | Inverse mutation | -0.15 |
| 1CUN | A | G201S | Inverse mutation | 0.95 |
| 1CUN | A | G212A | Inverse mutation | 1.45 |
| 1E65 | A | T31V | Inverse mutation | 1.08 |
| 1E65 | A | V50L | Inverse mutation | 0.36 |
| 1E65 | A | T95V | Inverse mutation | -0.96 |
| 1EY0 | A | V7L | Inverse mutation | 1.15 |
| 1EY0 | A | F9K | Inverse mutation | 1.03 |
| 1EY0 | A | C13T | Inverse mutation | 1.2 |
| 1EY0 | A | V14L | Inverse mutation | 1.63 |
| 1EY0 | A | M15I | Inverse mutation | 0.15 |
| 1EY0 | A | F19D | Inverse mutation | 1.28 |
| 1EY0 | A | K21D | Inverse mutation | -1.1 |
| 1EY0 | A | I22T | Inverse mutation | 0.61 |
| 1EY0 | A | I23V | Inverse mutation | -0.03 |
| 1EY0 | A | L23V | Inverse mutation | 0.02 |
| 1EY0 | A | F24K | Inverse mutation | 0.4 |
| 1EY0 | A | C33T | Inverse mutation | 1.04 |
| 1EY0 | A | I37L | Inverse mutation | 1.82 |
| 1EY0 | A | I39V | Inverse mutation | -0.11 |
| 1EY0 | A | L39V | Inverse mutation | 0.9 |
| 1EY0 | A | I41T | Inverse mutation | -0.86 |
| 1EY0 | A | C44T | Inverse mutation | 0.04 |
| 1EY0 | A | L51V | Inverse mutation | 0.1 |
| 1EY0 | A | F54Y | Inverse mutation | 0.38 |
| 1EY0 | A | V55G | Inverse mutation | 1.48 |
| 1EY0 | A | Q63K | Inverse mutation | 0.89 |
| 1EY0 | A | F65M | Inverse mutation | 1.62 |
| 1EY0 | A | I65M | Inverse mutation | 1.43 |
| 1EY0 | A | I66V | Inverse mutation | 0.76 |
| 1EY0 | A | E70K | Inverse mutation | 0.3 |
| 1EY0 | A | L72I | Inverse mutation | 0.23 |
| 1EY0 | A | L74V | Inverse mutation | 1.12 |
| 1EY0 | A | Q78K | Inverse mutation | 0.15 |
| 1EY0 | A | C82T | Inverse mutation | 0.19 |
| 1EY0 | A | I82T | Inverse mutation | -0.51 |
| 1EY0 | A | Q84K | Inverse mutation | 0.15 |
| 1EY0 | A | F86G | Inverse mutation | 1.99 |
| 1EY0 | A | I89L | Inverse mutation | 1.04 |
| 1EY0 | A | M92I | Inverse mutation | 1.75 |
| 1EY0 | A | I104V | Inverse mutation | -0.27 |
| 1EY0 | A | I111V | Inverse mutation | 0.74 |
| 1EY0 | A | L111V | Inverse mutation | 0.88 |
| 1EY0 | A | I114V | Inverse mutation | 0.15 |
| 1EY0 | A | E124H | Inverse mutation | -0.46 |
| 1EY0 | A | I125L | Inverse mutation | 0.96 |
| 1EY0 | A | V137L | Inverse mutation | 1.42 |
| 1EY0 | A | L139I | Inverse mutation | 0.09 |
| 1FNA | A | V34I | Inverse mutation | 0.11 |
| 1G4I | A | I22F | Inverse mutation | -1.43 |
| 1G4I | A | Y22F | Inverse mutation | -0.83 |
| 1G4I | A | A48H | Inverse mutation | 1.93 |
| 1G4I | A | Q48H | Inverse mutation | 0.49 |
| 1G4I | A | A106F | Inverse mutation | 1.23 |
| 1HFZ | A | Y32H | Inverse mutation | -0.07 |
| 1HFZ | A | W59I | Inverse mutation | 0.93 |
| 1HFZ | A | W107H | Inverse mutation | 1.72 |
| 1HFZ | A | Y107H | Inverse mutation | 0.19 |
| 1HFZ | A | E110L | Inverse mutation | 0.19 |
| 1HFZ | A | E114K | Inverse mutation | 0.65 |
| 1HMK | A | A8V | Inverse mutation | 0.83 |
| 1HMK | A | A27V | Inverse mutation | 1.24 |
| 1HMK | A | V89I | Inverse mutation | 0.86 |
| 1HMK | A | V95I | Inverse mutation | 1.72 |
| 1HMK | A | A96L | Inverse mutation | 1.75 |
| 1HMK | A | A110L | Inverse mutation | 0.35 |
| 1IET | A | R60D | Inverse mutation | -0.14 |
| 1IFC | A | Y6W | Inverse mutation | 0.87 |
| 1IFC | A | C60V | Inverse mutation | 0.07 |
| 1IFC | A | N60V | Inverse mutation | 0.83 |
| 1IFC | A | A65G | Inverse mutation | 0.94 |
| 1IFC | A | A68F | Inverse mutation | 0.42 |
| 1IHB | A | Q82F | Inverse mutation | 0.37 |
| 1JIW | I | A10D | Inverse mutation | 0.7 |
| 1K9Q | A | Y30L | Inverse mutation | -0.27 |
| 1LNI | A | V16T | Inverse mutation | -0.3 |
| 1LNI | A | F30Y | Inverse mutation | -0.4 |
| 1LNI | A | T43V | Inverse mutation | 0.5 |
| 1LNI | A | F55Y | Inverse mutation | 0.6 |
| 1LNI | A | V56T | Inverse mutation | 1.9 |
| 1LNI | A | N79D | Inverse mutation | -1.46 |
| 1LNI | A | F80Y | Inverse mutation | 1.5 |
| 1LNI | A | Q85H | Inverse mutation | 0 |
| 1LNI | A | K94Q | Inverse mutation | -0.56 |
| 1MGR | A | F84Y | Inverse mutation | 1 |
| 1MJC | A | W52S | Inverse mutation | 0.2 |
| 1QLP | A | L31A | Inverse mutation | -0.9 |
| 1QLP | A | I55V | Inverse mutation | 0.2 |
| 1QLP | A | W160Y | Inverse mutation | -1.18 |
| 1QLP | A | I183A | Inverse mutation | -1.8 |
| 1QLP | A | F238W | Inverse mutation | -0.98 |
| 1QLP | A | F248A | Inverse mutation | -1.8 |
| 1QLP | A | L248A | Inverse mutation | -0.35 |
| 1QLP | A | I284A | Inverse mutation | 0 |
| 1QLP | A | V284A | Inverse mutation | -0.8 |
| 1QLP | A | I321V | Inverse mutation | -0.6 |
| 1QLP | A | L364V | Inverse mutation | 0.3 |
| 1RG8 | A | F44L | Inverse mutation | -0.59 |
| 1RG8 | A | G106N | Inverse mutation | -0.16 |
| 1RG8 | A | I109V | Inverse mutation | 0.05 |
| 1RN1 | C | A23G | Inverse mutation | 1.2 |
| 1RN1 | C | A49D | Inverse mutation | -0.5 |
| 1SHF | A | L111I | Inverse mutation | 0.71 |
| 1SHF | A | M138V | Inverse mutation | 0.52 |
| 1TTQ | A | L22F | Inverse mutation | 1.05 |
| 1YYJ | A | A3L | Inverse mutation | 1.6 |
| 1YYJ | A | G20A | Inverse mutation | 1.97 |
| 1YYJ | A | A65F | Inverse mutation | 1.92 |
| 1ZG4 | A | F290W | Inverse mutation | 0.83 |
| 2A01 | A | R141L | Inverse mutation | 0.65 |
| 2A36 | A | A22T | Inverse mutation | -0.4 |
| 2A36 | A | F22T | Inverse mutation | -1.3 |
| 2A36 | A | L22T | Inverse mutation | -0.5 |
| 2A36 | A | N22T | Inverse mutation | -1.3 |
| 2NVH | A | Q4R | Inverse mutation | 0.57 |
| 2NVH | A | D7N | Inverse mutation | -0.09 |
| 2RN2 | A | A62H | Inverse mutation | -0.44 |
| 2TRX | A | L66T | Inverse mutation | 1.03 |
| 3GLY | A | A139G | Inverse mutation | 1.4 |
| 3GLY | A | A383G | Inverse mutation | -0.11 |
| 3SIL | A | L53A | Inverse mutation | -0.9 |
| 5DFR | A | V2I | Inverse mutation | 0.55 |
| 5DFR | A | M30W | Inverse mutation | 1.94 |
| 5DFR | A | T59N | Inverse mutation | 0.05 |
| 5DFR | A | W59N | Inverse mutation | 0.79 |
| 5DFR | A | S67G | Inverse mutation | 0.27 |
| 5DFR | A | T67G | Inverse mutation | 0.62 |
| 5DFR | A | A95G | Inverse mutation | 0.9 |
| 5DFR | A | C121G | Inverse mutation | 0.22 |
| 5DFR | A | H121G | Inverse mutation | 0.56 |
| 5PTI | A | T16A | Inverse mutation | 1.7 |
| 5PTI | A | V16A | Inverse mutation | 1.3 |

Supplementary Table 4. The S254 dataset with detailed prediction of mutDDG-SSM.

| PDB_ID | Mutation | pH | T | ΔΔG(kcal/mol) | direct_prediction | inverse_prediction |
| --- | --- | --- | --- | --- | --- | --- |
| 1A0F | S_11_A | 6.5 | 56.68 | -1.8 | -0.10260086 | 0.12564223 |
| 1BA3 | H_461_D | 7.8 | 25 | -1.744741874 | 0.1287072 | 0.006501553 |
| 1BA3 | H_489_D | 7.8 | 25 | 0.286806883 | -0.014107269 | 0.002465612 |
| 1BA3 | H_489_K | 7.8 | 25 | -0.286806883 | 0.08010237 | -0.040420957 |
| 1BA3 | H_489_M | 7.8 | 25 | -0.26290631 | 0.41604558 | -0.4338544 |
| 1BNL | D_76_A | 7.4 | 20 | -0.820574163 | -3.5465236 | 3.5102684 |
| 1DXX | K_18_N | 7 | 25 | -3.7 | -0.19660398 | 0.23790248 |
| 1E0L | W_30_A | 7 | 25 | -0.972734226 | -0.7352881 | 0.57823163 |
| 1E0L | W_30_F | 7 | 25 | 0.523386711 | 0.19047017 | -0.20884211 |
| 1E0L | Y_11_R | 7 | 25 | 0.633814532 | -0.30038697 | 0.15408674 |
| 1E0L | Y_19_L | 7 | 25 | -1.281500956 | -0.68424636 | 0.66274184 |
| 1E0W | W_266_A | 6 | 68 | -2.583732057 | -1.2003157 | 1.0524305 |
| 1E0W | W_266_F | 6 | 68 | -1.818181818 | -0.55071944 | 0.46415448 |
| 1E0W | W_266_H | 6 | 68 | -3.995215311 | -1.1502753 | 1.0394655 |
| 1E0W | W_274_A | 6 | 68 | 2.272727273 | -0.7383303 | 0.54210746 |
| 1E0W | W_274_F | 6 | 68 | 0.43062201 | 0.2793769 | -0.18780409 |
| 1E0W | W_274_H | 6 | 68 | 0.14354067 | -0.296333 | 0.6741587 |
| 1E0W | W_85_A | 6 | 68 | 1.076555024 | -1.4428391 | 1.4727885 |
| 1E0W | W_85_F | 6 | 68 | -2.200956938 | -0.5531527 | 0.3064912 |
| 1E0W | W_85_H | 6 | 68 | -0.526315789 | -0.6895604 | 0.64520323 |
| 1E0W | Y_172_A | 6 | 68 | -2.775119617 | -0.9770731 | 1.0122651 |
| 1E0W | Y_172_F | 6 | 68 | -0.502392344 | 0.074007094 | -0.089747705 |
| 1E0W | Y_172_S | 6 | 68 | 0.861244019 | -0.17544895 | 0.6439336 |
| 1F8I | F_345_A | 8 | 25 | -0.41 | -1.7086405 | 2.0500975 |
| 1FC1 | D_399_A | 7.4 | 20 | -0.7 | -1.2649289 | 1.207266 |
| 1FC1 | F_405_A | 7.4 | 20 | -2.5 | -2.7063494 | 3.007057 |
| 1FC1 | K_370_A | 7.4 | 20 | -1.1 | -0.7829942 | 0.62559706 |
| 1FC1 | K_392_A | 7.4 | 20 | -0.4 | -0.92152727 | 0.7442812 |
| 1FC1 | K_409_A | 7.4 | 20 | -2.4 | -0.9265418 | 0.77491564 |
| 1FC1 | L_351_A | 7.4 | 20 | -1.3 | -1.6554625 | 1.4345632 |
| 1FC1 | L_368_A | 7.4 | 20 | -2.2 | -3.125524 | 3.3463116 |
| 1FC1 | L_398_A | 7.4 | 20 | -0.1 | -1.194486 | 1.3855095 |
| 1FC1 | P_395_A | 7.4 | 20 | -3.2 | -0.8480586 | 0.8884678 |
| 1FC1 | Q_347_A | 7.4 | 20 | -1.1 | -0.5516856 | 0.6801839 |
| 1FC1 | T_350_A | 7.4 | 20 | -0.1 | -0.24885876 | 0.1363105 |
| 1FC1 | T_366_A | 7.4 | 20 | -2.3 | -0.82472086 | 1.0663713 |
| 1FC1 | T_394_A | 7.4 | 20 | -0.6 | -0.38001448 | 0.44983554 |
| 1FC1 | V_397_A | 7.4 | 20 | -0.6 | -1.5316004 | 1.5988009 |
| 1FC1 | Y_349_A | 7.4 | 20 | -0.7 | -2.2564504 | 2.0997453 |
| 1FC1 | Y_407_A | 7.4 | 20 | -2.1 | -2.3714273 | 2.3269014 |
| 1FT8 | I_264_V | 7 | 25 | -0.64 | -0.25170898 | 0.5017993 |
| 1FT8 | L_212_V | 7 | 25 | -1.38 | -0.68912274 | 0.77556074 |
| 1FT8 | L_238_I | 7 | 25 | -0.35 | -0.12047281 | 0.3951003 |
| 1FT8 | L_267_I | 7 | 25 | -0.11 | -0.24554738 | -0.07849983 |
| 1FT8 | L_293_I | 7 | 25 | -0.8 | -0.32699746 | 0.08187969 |
| 1FT8 | L_293_V | 7 | 25 | -1.41 | -0.6581968 | 0.7915714 |
| 1FT8 | L_315_A | 7 | 25 | -1.65 | -1.9189237 | 2.0565002 |
| 1FT8 | L_317_I | 7 | 25 | -0.85 | -0.12838537 | -0.10510733 |
| 1FT8 | L_348_I | 7 | 25 | -0.53 | -0.2527004 | 0.14581856 |
| 1FT8 | L_349_I | 7 | 25 | 0.05 | -0.44170842 | 0.2753412 |
| 1FT8 | M_216_L | 7 | 25 | 0.27 | -0.015824469 | -0.006717847 |
| 1FT8 | S_339_A | 7 | 25 | 0.32 | -0.027957559 | 0.092933945 |
| 1FT8 | V_287_A | 7 | 25 | -0.16 | -0.030516397 | 0.28427112 |
| 1G3P_Q129H_102-205 | A_147_K | 7 | 15 | -0.382409178 | 0.48335773 | -0.59283394 |
| 1G3P_Q129H_102-205 | D_160_A | 7 | 15 | -0.26290631 | -1.2003171 | 1.0779738 |
| 1G3P_Q129H_102-205 | D_160_C | 7 | 15 | -0.095602294 | -0.59189993 | 0.5808875 |
| 1G3P_Q129H_102-205 | D_160_E | 7 | 15 | -0.358508604 | 0.40325427 | -0.2838065 |
| 1G3P_Q129H_102-205 | D_160_F | 7 | 15 | -0.358508604 | 0.9564824 | -1.1293602 |
| 1G3P_Q129H_102-205 | D_160_G | 7 | 15 | -0.669216061 | 0.068183266 | -0.088943325 |
| 1G3P_Q129H_102-205 | D_160_H | 7 | 15 | 0.047801147 | 0.5025756 | -0.5237867 |
| 1G3P_Q129H_102-205 | D_160_I | 7 | 15 | -0.932122371 | 0.28277653 | -0.45881343 |
| 1G3P_Q129H_102-205 | D_160_K | 7 | 15 | -0.334608031 | 0.36097127 | -0.38698775 |
| 1G3P_Q129H_102-205 | D_160_L | 7 | 15 | -0.549713193 | 1.2358522 | -1.1206434 |
| 1G3P_Q129H_102-205 | D_160_M | 7 | 15 | -0.501912046 | 0.6114108 | -0.5837512 |
| 1G3P_Q129H_102-205 | D_160_N | 7 | 15 | 0.071701721 | 0.1857887 | -0.25318694 |
| 1G3P_Q129H_102-205 | D_160_P | 7 | 15 | -0.645315488 | -0.78344333 | 0.97230804 |
| 1G3P_Q129H_102-205 | D_160_Q | 7 | 15 | -0.478011472 | 0.22375254 | -0.18462397 |
| 1G3P_Q129H_102-205 | D_160_R | 7 | 15 | -0.669216061 | 0.98078954 | -0.9770606 |
| 1G3P_Q129H_102-205 | D_160_S | 7 | 15 | -0.286806883 | -0.34660655 | 0.49204856 |
| 1G3P_Q129H_102-205 | D_160_T | 7 | 15 | -0.740917782 | -0.8087033 | 0.42340732 |
| 1G3P_Q129H_102-205 | D_160_V | 7 | 15 | -1.12332696 | -0.34385687 | 0.3815847 |
| 1G3P_Q129H_102-205 | D_160_W | 7 | 15 | -0.167304015 | 1.3812345 | -1.344304 |
| 1G3P_Q129H_102-205 | D_160_Y | 7 | 15 | -0.071701721 | 1.3770382 | -1.3629149 |
| 1G3P_Q129H_102-205 | F_136_V | 7 | 15 | -3.824091778 | -0.6112693 | 0.9182757 |
| 1G3P_Q129H_102-205 | N_138_G | 7 | 15 | 0.932122371 | 0.28369468 | -0.22586589 |
| 1G3P_Q129H_102-205 | N_139_K | 7 | 15 | -0.59751434 | 0.25208524 | -0.34301674 |
| 1G3P_Q129H_102-205 | N_139_V | 7 | 15 | -2.748565966 | 0.8356677 | -0.97492075 |
| 1G3P_Q129H_102-205 | Q_137_N | 7 | 15 | -1.959847036 | 0.61472034 | -0.37527028 |
| 1G3P_Q129H_102-205 | Q_145_N | 7 | 15 | 0.621414914 | 0.08798431 | -0.22178546 |
| 1G3P_Q129H_102-205 | R_144_V | 7 | 15 | 0.908221797 | 0.13227908 | 0.16840401 |
| 1GLU | C_500_A | 6.5 | 25 | -1.08 | 0.13715672 | -0.37026367 |
| 1GUA | A_118_G | 7 | 25 | -1.963033779 | -0.22787575 | 0.41227192 |
| 1GUA | A_118_L | 7 | 25 | -1.951083493 | 0.19610646 | -0.11235932 |
| 1GUA | A_85_G | 7 | 25 | -2.148661568 | -0.3707879 | 0.37276334 |
| 1GUA | A_97_G | 7 | 25 | -0.862810707 | -2.259635 | 2.2057958 |
| 1GUA | C_81_A | 7 | 25 | 0.282823454 | -2.1448789 | 2.0579956 |
| 1GUA | C_81_I | 7 | 25 | -0.912205226 | 0.6674272 | -0.50059605 |
| 1GUA | C_95_A | 7 | 25 | -0.079668579 | -0.02358127 | 0.07863678 |
| 1GUA | C_96_A | 7 | 25 | -1.759082218 | -1.7905171 | 1.7263092 |
| 1GUA | C_96_L | 7 | 25 | -0.043021033 | 9.37E-06 | -0.16849206 |
| 1GUA | C_96_M | 7 | 25 | -0.336998088 | 0.37687173 | -0.32896355 |
| 1GUA | D_117_A | 7 | 25 | -0.705863607 | -0.75839233 | 0.91754305 |
| 1GUA | D_129_A | 7 | 25 | -0.85484385 | -0.47401205 | 0.5353827 |
| 1GUA | D_80_A | 7 | 25 | -0.493148502 | -2.3901906 | 2.4594276 |
| 1GUA | E_104_A | 7 | 25 | 0.02071383 | -0.14074889 | 0.2901948 |
| 1GUA | E_124_A | 7 | 25 | -0.101179095 | -0.9012529 | 0.87338865 |
| 1GUA | E_125_A | 7 | 25 | -0.591140854 | -0.23257606 | 0.3569404 |
| 1GUA | I_58_A | 7 | 25 | -3.274378585 | -1.12341 | 0.8394655 |
| 1GUA | I_58_F | 7 | 25 | -1.298597833 | 1.0208647 | -0.79725796 |
| 1GUA | I_58_L | 7 | 25 | -0.771191842 | 1.0597813 | -1.0539777 |
| 1GUA | K_109_A | 7 | 25 | -0.327437859 | -1.4601674 | 1.465864 |
| 1GUA | L_112_A | 7 | 25 | -2.593212237 | -2.5352216 | 2.6311173 |
| 1GUA | L_121_A | 7 | 25 | -2.534257489 | -0.5121171 | 0.6042767 |
| 1GUA | L_126_A | 7 | 25 | -3.405035054 | -1.2439506 | 1.0459521 |
| 1GUA | L_62_A | 7 | 25 | -3.460006373 | -0.4317329 | 0.4132505 |
| 1GUA | L_78_A | 7 | 25 | -3.802581262 | -1.9962628 | 2.0286603 |
| 1GUA | L_82_A | 7 | 25 | -3.529318037 | -2.7077787 | 2.9469829 |
| 1GUA | L_82_I | 7.5 | 8 | -1.6 | 0.2552426 | -0.46683693 |
| 1GUA | L_82_V | 7.5 | 8 | -1.7 | -0.2921802 | 0.416511 |
| 1GUA | L_86_A | 7 | 25 | -3.225780752 | 0.22883372 | -0.27152294 |
| 1GUA | L_91_A | 7 | 25 | -1.575047801 | -0.20477667 | 0.363365 |
| 1GUA | M_76_A | 7 | 25 | -0.960803059 | -0.0945063 | 0.38571003 |
| 1GUA | N_56_M | 7 | 25 | 0.270873168 | -0.22897854 | 0.19706644 |
| 1GUA | N_64_A | 7 | 25 | -1.140057361 | -0.56378067 | 0.6112173 |
| 1GUA | P_63_A | 7 | 25 | -1.708094328 | -0.15822479 | 0.33147115 |
| 1GUA | P_93_A | 7 | 25 | -0.082058636 | -2.858435 | 2.6697319 |
| 1GUA | Q_66_A | 7 | 25 | -0.503505417 | -0.061965715 | 0.036582343 |
| 1GUA | R_100_A | 7 | 25 | -0.693116635 | -1.4055548 | 1.3676096 |
| 1GUA | R_59_A | 7 | 25 | 0.178457616 | 0.21531777 | -0.37810713 |
| 1GUA | R_89_L | 7 | 25 | 1.07791587 | 1.4512616 | -1.417526 |
| 1GUA | S_77_A | 7 | 25 | -1.572657744 | 0.78860855 | -0.8973821 |
| 1GUA | S_77_T | 7 | 25 | 0.580783939 | 1.2965344 | -1.2532431 |
| 1GUA | T_68_A | 7 | 25 | -0.109145953 | -1.0454175 | 1.0645773 |
| 1GUA | V_128_A | 7 | 25 | -3.368387508 | -0.40783066 | 0.37156814 |
| 1GUA | V_60_A | 7 | 25 | -2.251434034 | 0.15478711 | -0.25505754 |
| 1GUA | V_69_A | 7 | 25 | -1.116156788 | -0.06071509 | 0.031065172 |
| 1GUA | V_70_A | 7 | 25 | -1.770235819 | -1.4091821 | 1.4954836 |
| 1GUA | V_72_A | 7 | 25 | -1.052421925 | -2.174368 | 2.2096577 |
| 1GUA | V_72_I | 7 | 25 | -0.863607393 | -0.010139625 | -0.25011134 |
| 1GUA | V_98_A | 7 | 25 | -1.980560867 | -0.3664573 | 0.4199447 |
| 1HCQ | C_67_A | 6.5 | 25 | -2.09 | -0.48605815 | 0.3369148 |
| 1HCQ | C_67_S | 6.5 | 25 | -1.99 | -0.93446195 | 1.1053185 |
| 1IV7 | A_119_C | 8 | 25 | -0.7 | 0.5688872 | -0.60524184 |
| 1IV7 | A_173_C | 8 | 25 | 0.5 | 1.9228426 | -2.1648555 |
| 1IV7 | C_141_A | 8 | 25 | 0.5 | -1.9112873 | 1.8251435 |
| 1IV7 | F_189_C | 8 | 25 | -2.5 | -0.8666035 | 0.94756955 |
| 1IV7 | L_132_A | 8 | 25 | -2 | -1.4480426 | 1.712952 |
| 1IV7 | L_162_C | 8 | 25 | -1.9 | 0.41599765 | -0.61206424 |
| 1J8I | K_25_A | 6 | 25 | 0.1 | -0.99207675 | 1.1303976 |
| 1J8I | K_66_A | 6 | 25 | 0.1 | -0.12171775 | 0.18178475 |
| 1J8I | R_23_A | 6 | 25 | 0.8 | -1.046021 | 0.9829561 |
| 1J8I | R_35_A | 6 | 25 | -0.1 | -0.7888058 | 0.62972856 |
| 1J8I | R_43_A | 6 | 25 | 0.7 | -0.65164775 | 0.7030519 |
| 1J8I | R_9_A | 6 | 25 | -0.1 | -0.4738795 | 0.21270056 |
| 1JL9 | A_25_C | 8.4 | 22 | -1.44 | 0.7961228 | -1.0114748 |
| 1JLV | E_64_D | 6.5 | 42 | -1.161567878 | 0.4491702 | -0.654183 |
| 1JLV | E_64_N | 6.5 | 42 | -2.304015296 | 0.502579 | -0.5226208 |
| 1JLV | E_64_Q | 6.5 | 42 | -2.301625239 | -0.30678788 | 0.056865055 |
| 1L6H | F_39_A | 7 | 25 | -1.696940727 | -1.4705204 | 1.4316818 |
| 1L6H | Y_45_A | 7 | 25 | -0.549713193 | -2.147119 | 1.893404 |
| 1L6H | Y_48_A | 7 | 25 | -1.481835564 | -1.1830108 | 1.2347754 |
| 1LVM | E_106_G | 8 | 25 | 1.4 | -0.029027227 | -0.080992594 |
| 1LVM | K_45_F | 8 | 25 | 0.43 | 0.21502905 | 0.16682632 |
| 1LVM | L_56_V | 8 | 25 | 0 | 0.31487983 | -0.2774437 |
| 1LVM | Q_58_F | 8 | 25 | 0.6 | 2.122477 | -2.1678443 |
| 1LVM | S_135_G | 8 | 25 | 0 | 0.6760959 | -0.55973685 |
| 1NMV | W_11_L | 7 | 25 | 1 | -0.2851644 | 0.31926435 |
| 1NMV | W_34_L | 7 | 25 | -1.19 | 0.28339377 | -0.46228036 |
| 1NMV | W_73_L | 7 | 25 | -0.478 | -0.17456451 | 0.06782739 |
| 1O6X | A_31_G | 7 | 25 | -0.85 | -0.6259022 | 0.7830435 |
| 1O6X | A_50_G | 7 | 25 | -1.55 | -1.216806 | 1.1181192 |
| 1O6X | D_38_A | 7 | 25 | 0.18 | -0.6324401 | 0.8286703 |
| 1O6X | E_14_A | 7 | 25 | -0.35 | -0.6905314 | 0.9176772 |
| 1O6X | E_20_G | 7 | 25 | -1.21 | -1.1217849 | 0.92761743 |
| 1O6X | F_39_L | 7 | 25 | -1.99 | -0.19968867 | -0.18126328 |
| 1O6X | F_65_A | 7 | 25 | -1.29 | -2.3858676 | 2.0391042 |
| 1O6X | H_51_A | 7 | 25 | -0.705 | -1.5003042 | 1.6888599 |
| 1O6X | I_15_V | 7 | 25 | -0.465 | -0.32960984 | 0.5090105 |
| 1O6X | I_23_V | 7 | 25 | 0.16 | -0.38118094 | 0.490335 |
| 1O6X | I_71_V | 7 | 25 | -1.405 | -0.47172183 | 0.6030859 |
| 1O6X | I_75_A | 7 | 25 | -1.605 | -2.3620563 | 2.421748 |
| 1O6X | K_41_A | 7 | 25 | -2.14 | -0.10009612 | 0.011841176 |
| 1O6X | L_26_V | 7 | 25 | -1.135 | 0.16574347 | -0.13793162 |
| 1O6X | N_58_A | 7 | 25 | -0.24 | -0.122022346 | -0.062093884 |
| 1O6X | Q_60_G | 7 | 25 | -0.445 | -0.9948801 | 1.1758378 |
| 1O6X | V_12_A | 7 | 25 | -1.45 | -1.5313208 | 1.4526689 |
| 1O6X | V_52_A | 7 | 25 | -1.09 | -2.1657538 | 2.0034413 |
| 1O6X | V_64_G | 7 | 25 | -0.8 | -0.3736279 | 0.56465596 |
| 1O6X | Y_73_L | 7 | 25 | -2.94 | -0.80819166 | 0.92456836 |
| 1PRG | E_460_K | 8 | 10 | -0.17 | -0.71966594 | 0.9292835 |
| 1PRG | F_360_L | 8 | 10 | -0.4 | 0.1885424 | -0.06541937 |
| 1PRG | P_467_L | 8 | 10 | 0.11 | 2.289009 | -2.3691869 |
| 1PRG | Q_286_P | 8 | 10 | -0.3 | -0.9648515 | 1.1688299 |
| 1PRG | R_288_H | 8 | 10 | 0.06 | -0.58891135 | 0.8252846 |
| 1PRG | R_357_A | 8 | 10 | 0.19 | -0.5368555 | 0.23329028 |
| 1PRG | V_290_M | 8 | 10 | 0.03 | -0.12104088 | 0.16372009 |
| 2ARF | H_1069_Q | 7 | 20 | -0.717017208 | 0.20106766 | -0.1073354 |
| 2CLR | H_13_F | 7.4 | 25 | 0.119502868 | 0.6195243 | -0.5478176 |
| 2CLR | H_31_F | 7.4 | 25 | 2.605162524 | 0.50380206 | -0.51244444 |
| 2CLR | H_51_F | 7.4 | 25 | -1.912045889 | -0.2511614 | 0.39599866 |
| 2CLR | H_84_A | 7.4 | 25 | -2.629063098 | -1.4474599 | 1.5476866 |
| 2H3F | E_12_A | 7 | 25 | 1.07 | -0.4723371 | 0.29659647 |
| 2JOF | D_9_E | 7 | 27 | -0.291586998 | 0.5148866 | -0.4602552 |
| 2JOF | P_17_A | 7 | 27 | -0.645315488 | -0.5711031 | 0.5603089 |
| 2JOF | P_18_A | 7 | 27 | -0.239005736 | -0.68212295 | 0.75716555 |
| 2JOF | P_19_A | 7 | 27 | -2.820267686 | -0.989419 | 0.6190305 |
| 2JOF | R_16_K | 7 | 27 | -0.56166348 | -0.38081264 | 0.18108426 |
| 2JOF | S_14_A | 7 | 27 | -1.434034417 | -0.20011513 | 0.28048202 |
| 2JUC | C_57_D | 5.7 | 25 | -2.55 | 0.003455255 | -0.082332425 |
| 2JUC | C_57_S | 5.7 | 25 | -0.71 | -0.15365288 | 0.11475776 |
| 2JUC | R_18_A | 5.7 | 25 | -1.75 | -1.0187389 | 1.0315262 |
| 2N7Z | P_88_A | 8 | 25 | -0.6 | -1.0214583 | 0.9440525 |
| 2NTE | I_738_V | 7.5 | 10 | -2 | -0.5988455 | 0.62374103 |
| 2NTE | R_658_C | 7.5 | 10 | -2.3 | -0.8374054 | 1.0932137 |
| 2PR5 | A_33_Y | 7.9 | 25 | 0.5 | 0.9096653 | -0.8114521 |
| 2PR5 | A_81_M | 7.9 | 25 | -0.4 | 0.6386907 | -0.56040144 |
| 2PR5 | D_109_E | 7.9 | 25 | 0.3 | -0.31270045 | 0.06547244 |
| 2PR5 | H_22_K | 7.9 | 25 | 0.3 | 0.17118913 | -0.001789773 |
| 2PR5 | H_22_W | 7.9 | 25 | 1.2 | 0.24040803 | -0.2287421 |
| 2PR5 | M_111_F | 7.9 | 25 | 2.9 | 0.17586137 | -0.32512403 |
| 2PR5 | N_107_F | 7.9 | 25 | 0.9 | 1.3720362 | -1.3656142 |
| 2PR5 | N_107_Y | 7.9 | 25 | 0.8 | 2.3908427 | -2.4800582 |
| 2PR5 | N_124_F | 7.9 | 25 | 1.1 | 0.9692041 | -0.8219821 |
| 2PR5 | N_124_Y | 7.9 | 25 | 2.6 | 1.3095181 | -1.3179322 |
| 2PR5 | T_30_M | 7.9 | 25 | 0.5 | 0.100695275 | -0.055268906 |
| 2PR5 | T_50_M | 7.9 | 25 | -0.5 | 1.6966741 | -1.4431028 |
| 2PR5 | T_54_Y | 7.9 | 25 | 0.2 | 0.24512935 | -0.11575706 |
| 2PR5 | V_120_I | 7.9 | 25 | 1.4 | 0.4489382 | -0.5150231 |
| 2PR5 | V_25_I | 7.9 | 25 | 0.4 | 0.4934315 | -0.43289834 |
| 2PR5 | V_88_L | 7.9 | 25 | -0.2 | -0.54908675 | 0.5093939 |
| 2PR5 | V_90_I | 7.9 | 25 | -0.7 | 0.7736013 | -0.70372295 |
| 2Q98 | H_138_A | 7.8 | 25 | 0.58 | 1.1598723 | -1.0737718 |
| 2Q98 | H_173_A | 7.8 | 25 | -0.35 | -0.06330773 | 0.025428373 |
| 2Q98 | H_180_A | 7.8 | 25 | -0.04 | 0.5246377 | -0.58847326 |
| 2Q98 | H_195_A | 7.8 | 25 | 0.12 | -0.006645233 | -0.001066859 |
| 2Q98 | H_27_A | 7.8 | 25 | 1.29 | 0.6210639 | -0.6370748 |
| 2Q98 | H_30_A | 7.8 | 25 | 2.05 | 2.143183 | -2.151317 |
| 2Q98 | H_46_A | 7.8 | 25 | 1.55 | 0.655571 | -0.6416143 |
| 2Q98 | H_59_A | 7.8 | 25 | -0.02 | 0.86413175 | -0.8931759 |
| 2Q98 | H_97_A | 7.8 | 25 | 0.33 | 0.5775007 | -0.552887 |
| 3C2I | D_156_E | 7.5 | 25 | -0.875 | 0.21582527 | -0.16958138 |
| 3C2I | F_155_S | 7.5 | 25 | -0.43 | -1.0912443 | 1.20846 |
| 3C2I | L_100_V | 7.5 | 25 | -1.105 | -1.0774609 | 1.100924 |
| 3C2I | P_152_R | 7.5 | 25 | -1.46 | 0.5638884 | -0.63290274 |
| 3C2I | R_106_Q | 7.5 | 25 | -0.12 | -0.92766917 | 0.9724676 |
| 3C2I | R_106_W | 7.5 | 25 | -0.04 | 0.3116917 | -0.43346134 |
| 3C2I | R_111_G | 7.5 | 25 | 0.425 | -0.62176055 | 0.70003915 |
| 3C2I | R_133_C | 7.5 | 25 | -0.365 | 0.4128005 | -0.475699 |
| 3C2I | R_133_H | 7.5 | 25 | -0.235 | -0.13804013 | 0.029429447 |
| 3C2I | S_134_C | 7.5 | 25 | -1.045 | 0.29901427 | 0.055308916 |
| 3C2I | T_158_A | 7.5 | 25 | -0.47 | -0.54417527 | 0.5590942 |
| 3C2I | T_158_M | 7.5 | 25 | -0.25 | 0.018035293 | 0.03022251 |
| 3D2C | M_134_E | 7.2 | 25 | 0.66 | -0.3178649 | 0.4636186 |
| 3D2C | M_137_P | 7.2 | 25 | 0.98 | -0.8995821 | 0.7047554 |
| 3D2C | S_163_P | 7.2 | 25 | -0.25 | 0.37815607 | -0.6369484 |
| 3FFN | D_187_N | 7 | 25 | -0.9 | -0.11278163 | 0.1326513 |
| 4BUQ | Y_137_A | 7.4 | 25 | 0.89 | -0.78166705 | 1.0564653 |
| 4BUQ | Y_48_A | 7.4 | 25 | -1.435 | -0.97889984 | 1.2792263 |
| 4HE7 | A_19_D | 7.6 | 25 | -0.24 | 0.60761315 | -0.6097611 |
| 4HE7 | A_19_G | 7.6 | 25 | 0.06 | -0.028275913 | -0.09697001 |
| 4HE7 | A_19_K | 7.6 | 25 | -0.46 | 0.2494761 | -0.29428405 |
| 5VP3 | R_39_K | 8 | 25 | 0.413479924 | -0.2045968 | 0.1544393 |
| 5VP3 | S_128_G | 8 | 25 | -0.377629063 | -0.7301634 | 0.7315546 |
| 5VP3 | V_183_T | 8 | 25 | 0.353728489 | -0.19731095 | 0.13648522 |

Supplementary Table 5. The S615 dataset with detailed prediction of mutDDG-SSM.

| pdb_code | mutation | PDB_Mut | TEMP | pH | ΔΔG(kcal/mol) | direct_prediction | inverse_prediction |
| --- | --- | --- | --- | --- | --- | --- | --- |
| 1A0F | SA11A | S11A | 329.83 | 6.5 | -1.8 | -0.306815253 | 0.308203883 |
| 1A7V | AA104H | A104H | 298.15 | 6.5 | -2.69 | 0.528526258 | -0.519003072 |
| 1A7V | AA66H | A66H | 298.15 | 6.5 | -1.98 | 0.706080324 | -0.727032684 |
| 1A7V | AA91H | A91H | 298.15 | 6.5 | -1.7 | 0.609567169 | -0.601534716 |
| 1A7V | DA3H | D3H | 298.15 | 6.5 | -1.36 | 0.059144258 | -0.052466217 |
| 1A7V | DA58H | D58H | 298.15 | 6.5 | -2.05 | 0.157742983 | -0.157994175 |
| 1A7V | EA73H | E73H | 298.15 | 6.5 | -3.01 | -0.123827279 | 0.127591198 |
| 1A7V | KA13H | K13H | 298.15 | 6.5 | -0.6 | -0.149585122 | 0.153470817 |
| 1A7V | KA20H | K20H | 298.15 | 6.5 | -2.88 | -0.113490437 | 0.114529896 |
| 1A7V | KA31H | K31H | 298.15 | 6.5 | -1.36 | -0.254766294 | 0.26005837 |
| 1A7V | KA39H | K39H | 298.15 | 6.5 | -1.86 | -0.092116807 | 0.090383826 |
| 1A7V | KA49H | K49H | 298.15 | 6.5 | -2.02 | -0.226314863 | 0.225579952 |
| 1A7V | KA84H | K84H | 298.15 | 6.5 | -2.88 | -0.189280243 | 0.191832749 |
| 1A7V | KA97H | K97H | 298.15 | 6.5 | -1.97 | -0.119404162 | 0.121080253 |
| 1BA3 | HA461D | H461D | 298.15 | 7.8 | -1.745 | -0.190096636 | 0.195322507 |
| 1BA3 | HA489D | H489D | 298.15 | 7.8 | 0.287 | -0.061464953 | 0.068097131 |
| 1BA3 | HA489K | H489K | 298.15 | 7.8 | -0.287 | 0.022961923 | -0.018916369 |
| 1BA3 | HA489M | H489M | 298.15 | 7.8 | -0.263 | 0.351076641 | -0.34184241 |
| 1BFM | MA35W | M35W | 298.15 | 7.2 | -0.7 | 0.272900724 | -0.27462025 |
| 1BFM | MA35Y | M35Y | 298.15 | 7.2 | -0.4 | 0.298229704 | -0.309027545 |
| 1D5G | HA71Y | H71Y | 298.15 | 6.8 | -0.04 | 0.050149091 | -0.054581508 |
| 1D5G | IA20F | I20F | 298.15 | 6.8 | 0.91 | 0.232480761 | -0.222141186 |
| 1DIV | EA100A | E100A | 298.15 | 8 | -0.98 | -0.501660568 | 0.502040149 |
| 1DIV | EA100G | E100G | 298.15 | 8 | -1.52 | -0.836915281 | 0.845314897 |
| 1DIV | IA115A | I115A | 298.15 | 8 | -3.52 | -2.095228245 | 2.095132905 |
| 1DIV | IA121A | I121A | 298.15 | 8 | -2.88 | -2.455693491 | 2.442922237 |
| 1DIV | IA79A | I79A | 298.15 | 8 | -3.49 | -2.44409149 | 2.420671143 |
| 1DIV | IA93A | I93A | 298.15 | 8 | -3.19 | -2.156625167 | 2.157148419 |
| 1DIV | IA98A | I98A | 298.15 | 8 | -4.28 | -1.903457989 | 1.900706554 |
| 1DIV | KA96A | K96A | 298.15 | 8 | 0.61 | -0.560666747 | 0.558647472 |
| 1DIV | KA96G | K96G | 298.15 | 8 | -0.2 | -1.114257328 | 1.116054937 |
| 1DIV | LA102A | L102A | 298.15 | 8 | -2.37 | -1.755131103 | 1.73877987 |
| 1DIV | LA108A | L108A | 298.15 | 8 | -2.33 | -1.695040969 | 1.698790614 |
| 1DIV | LA110A | L110A | 298.15 | 8 | -3.21 | -1.82453143 | 1.823184062 |
| 1DIV | LA117A | L117A | 298.15 | 8 | -0.82 | -1.997980018 | 1.976710393 |
| 1DIV | LA133A | L133A | 298.15 | 8 | -2.94 | -1.894017432 | 1.884392632 |
| 1DIV | LA141A | L141A | 298.15 | 8 | -3.56 | -2.377727577 | 2.358583356 |
| 1DIV | LA72A | L72A | 298.15 | 8 | -3.77 | -2.167579555 | 2.139290156 |
| 1DIV | VA129A | V129A | 298.15 | 8 | -1.81 | -1.601988042 | 1.603335461 |
| 1DIV | VA131A | V131A | 298.15 | 8 | -1.93 | -1.503801496 | 1.503167951 |
| 1DIV | VA137A | V137A | 298.15 | 8 | -2.41 | -1.550898747 | 1.534768681 |
| 1DIV | VA143A | V143A | 298.15 | 8 | -3.25 | -1.494132217 | 1.495692116 |
| 1DIV | VA145A | V145A | 298.15 | 8 | -1.92 | -1.351385165 | 1.337361444 |
| 1DXX | KA18N | K18N | 298.15 | 7 | -3.7 | -0.164018033 | 0.167486879 |
| 1EKG | LA198C | L198C | 298.15 | 7 | -1 | -0.30510671 | 0.303591806 |
| 1EKG | LA200C | L200C | 298.15 | 7 | -2 | -0.320251322 | 0.321124748 |
| 1EKG | LA203C | L203C | 298.15 | 7 | 1 | -0.240091422 | 0.232008061 |
| 1EKG | SA181F | S181F | 293.15 | 8 | -2.6 | 0.835474227 | -0.85328054 |
| 1F8I | FA345A | F345A | 298.15 | 8 | -0.455 | -2.06831412 | 2.071764505 |
| 1FH5 | SH18N | S18N | 298.15 | 7 | 0.6 | -0.000559944 | -0.002166703 |
| 1FT8 | IA264V | I264V | 298.15 | 7 | -0.64 | -0.252936261 | 0.260328669 |
| 1FT8 | LA212V | L212V | 298.15 | 7 | -1.38 | -0.191562533 | 0.180912438 |
| 1FT8 | LA238I | L238I | 298.15 | 7 | -0.35 | 0.201440347 | -0.199198499 |
| 1FT8 | LA267I | L267I | 298.15 | 7 | -0.11 | 0.167781284 | -0.163590221 |
| 1FT8 | LA293I | L293I | 298.15 | 7 | -0.8 | 0.254718519 | -0.246821636 |
| 1FT8 | LA293V | L293V | 298.15 | 7 | -1.41 | -0.267654046 | 0.350166373 |
| 1FT8 | LA315A | L315A | 298.15 | 7 | -1.65 | -1.103545848 | 1.082381357 |
| 1FT8 | LA317I | L317I | 298.15 | 7 | -0.85 | 0.1514396 | -0.150155776 |
| 1FT8 | LA348I | L348I | 298.15 | 7 | -0.53 | 0.250962623 | -0.249197714 |
| 1FT8 | LA349I | L349I | 298.15 | 7 | 0.05 | 0.217805661 | -0.218931358 |
| 1FT8 | MA216L | M216L | 298.15 | 7 | 0.27 | -0.173815628 | 0.16785265 |
| 1FT8 | SA339A | S339A | 298.15 | 7 | 0.32 | -0.270895186 | 0.259659018 |
| 1FT8 | VA287A | V287A | 298.15 | 7 | -0.16 | -0.698162968 | 0.69697376 |
| 1G3P | AA147K | A147K | 313.15 | 7 | -0.287 | 0.660065165 | -0.666198367 |
| 1G3P | DA160A | D160A | 288.15 | 7 | -0.263 | -0.611442887 | 0.62032603 |
| 1G3P | DA160C | D160C | 288.15 | 7 | -0.096 | 0.161379126 | -0.162535315 |
| 1G3P | DA160E | D160E | 288.15 | 7 | -0.359 | 0.199335536 | -0.191097229 |
| 1G3P | DA160F | D160F | 288.15 | 7 | -0.359 | 0.425644423 | -0.439813501 |
| 1G3P | DA160G | D160G | 288.15 | 7 | -0.669 | -0.616745323 | 0.608917078 |
| 1G3P | DA160H | D160H | 288.15 | 7 | 0.048 | 0.033791044 | -0.024924023 |
| 1G3P | DA160I | D160I | 288.15 | 7 | -0.932 | 0.427821413 | -0.418277967 |
| 1G3P | DA160K | D160K | 288.15 | 7 | -0.335 | 0.208650175 | -0.198790047 |
| 1G3P | DA160L | D160L | 288.15 | 7 | -0.55 | 0.454529884 | -0.457814142 |
| 1G3P | DA160M | D160M | 288.15 | 7 | -0.502 | 0.27291707 | -0.278676069 |
| 1G3P | DA160N | D160N | 288.15 | 7 | 0.072 | -0.105710743 | 0.112102666 |
| 1G3P | DA160P | D160P | 288.15 | 7 | -0.645 | -0.284078937 | 0.278620821 |
| 1G3P | DA160Q | D160Q | 288.15 | 7 | -0.478 | 0.007358004 | -0.004591451 |
| 1G3P | DA160R | D160R | 288.15 | 7 | -0.669 | 0.174567215 | -0.163922554 |
| 1G3P | DA160S | D160S | 288.15 | 7 | -0.287 | -0.280518555 | 0.279409617 |
| 1G3P | DA160T | D160T | 288.15 | 7 | -0.741 | -0.256000729 | 0.24683646 |
| 1G3P | DA160V | D160V | 288.15 | 7 | -1.123 | 0.14511749 | -0.138859418 |
| 1G3P | DA160W | D160W | 288.15 | 7 | -0.167 | 0.394718518 | -0.416380348 |
| 1G3P | DA160Y | D160Y | 288.15 | 7 | -0.072 | 0.391520298 | -0.377839703 |
| 1G3P | FA136V | F136V | 313.15 | 7 | -3.705 | -0.200280456 | 0.196772492 |
| 1G3P | NA138G | N138G | 313.15 | 7 | 0.765 | -0.41220349 | 0.419089646 |
| 1G3P | NA139K | N139K | 313.15 | 7 | -0.43 | 0.226671174 | -0.230341947 |
| 1G3P | NA139V | N139V | 313.15 | 7 | -2.032 | 0.678929955 | -0.688169553 |
| 1G3P | QA137N | Q137N | 313.15 | 7 | -1.793 | 0.050595382 | -0.035779542 |
| 1G3P | QA145N | Q145N | 313.15 | 7 | 0.263 | -0.065441533 | 0.072267226 |
| 1G3P | RA144V | R144V | 313.15 | 7 | 0.765 | 0.118361645 | -0.130925699 |
| 1GLU | CA500A | C500A | 298.15 | 6.5 | -1.22 | -0.480470787 | 0.489818896 |
| 1GUA | AB118G | A118G | 298.15 | 7 | -1.963 | -0.462496022 | 0.457698015 |
| 1GUA | AB118L | A118L | 298.15 | 7 | -1.951 | 1.530695604 | -1.53817565 |
| 1GUA | AB85G | A85G | 298.15 | 7 | -2.149 | -0.693450517 | 0.689550637 |
| 1GUA | AB97G | A97G | 298.15 | 7 | -0.863 | -0.818021605 | 0.821245149 |
| 1GUA | CB81A | C81A | 298.15 | 7 | 0.283 | -0.710735956 | 0.718099875 |
| 1GUA | CB81I | C81I | 298.15 | 7 | -0.912 | 0.705270671 | -0.69163612 |
| 1GUA | CB95A | C95A | 298.15 | 7 | -0.08 | -0.499683008 | 0.499478231 |
| 1GUA | CB96A | C96A | 298.15 | 7 | -1.759 | -1.155542246 | 1.141244154 |
| 1GUA | CB96L | C96L | 298.15 | 7 | -0.043 | -0.20809647 | 0.188209657 |
| 1GUA | CB96M | C96M | 298.15 | 7 | -0.337 | 0.535959841 | -0.569566687 |
| 1GUA | DB117A | D117A | 298.15 | 7 | -0.706 | -0.688399102 | 0.682506661 |
| 1GUA | DB129A | D129A | 298.15 | 7 | -0.855 | -0.626712334 | 0.616896146 |
| 1GUA | DB80A | D80A | 298.15 | 7 | -0.493 | -0.624367109 | 0.62777415 |
| 1GUA | EB104A | E104A | 298.15 | 7 | 0.021 | -0.37128841 | 0.366039845 |
| 1GUA | EB124A | E124A | 298.15 | 7 | -0.101 | -0.55893748 | 0.56328558 |
| 1GUA | EB125A | E125A | 298.15 | 7 | -0.591 | -0.592721577 | 0.603765911 |
| 1GUA | IB58A | I58A | 298.15 | 7 | -3.274 | -2.284046606 | 2.270540127 |
| 1GUA | IB58F | I58F | 298.15 | 7 | -1.299 | -0.668971754 | 0.664444269 |
| 1GUA | IB58L | I58L | 298.15 | 7 | -0.771 | -0.251286871 | 0.248047958 |
| 1GUA | KB109A | K109A | 298.15 | 7 | -0.327 | -0.672410739 | 0.676477532 |
| 1GUA | LB112A | L112A | 298.15 | 7 | -2.593 | -1.848369171 | 1.850845104 |
| 1GUA | LB121A | L121A | 298.15 | 7 | -2.534 | -1.685325014 | 1.677501784 |
| 1GUA | LB126A | L126A | 298.15 | 7 | -3.405 | -2.1477499 | 2.133903647 |
| 1GUA | LB62A | L62A | 298.15 | 7 | -3.46 | -2.234749436 | 2.231399779 |
| 1GUA | LB78A | L78A | 298.15 | 7 | -3.803 | -1.551626943 | 1.545516032 |
| 1GUA | LB82A | L82A | 298.15 | 7 | -3.529 | -1.684682855 | 1.682077793 |
| 1GUA | LB82I | L82I | 281.15 | 7.5 | -1.6 | 0.204602396 | -0.204015949 |
| 1GUA | LB82V | L82V | 281.15 | 7.5 | -1.7 | -0.160454254 | 0.165790601 |
| 1GUA | LB86A | L86A | 298.15 | 7 | -3.226 | -1.81139723 | 1.806496883 |
| 1GUA | LB91A | L91A | 298.15 | 7 | -1.575 | -1.682109525 | 1.674898681 |
| 1GUA | MB76A | M76A | 298.15 | 7 | -0.961 | -1.187230378 | 1.182976431 |
| 1GUA | NB56M | N56M | 298.15 | 7 | 0.271 | 0.949603629 | -0.964320335 |
| 1GUA | NB64A | N64A | 298.15 | 7 | -1.14 | -0.523655467 | 0.54267084 |
| 1GUA | PB63A | P63A | 298.15 | 7 | -1.708 | -0.540570337 | 0.543577644 |
| 1GUA | PB93A | P93A | 298.15 | 7 | -0.082 | -0.679700481 | 0.683405209 |
| 1GUA | QB66A | Q66A | 298.15 | 7 | -0.504 | -0.63593479 | 0.640398842 |
| 1GUA | RB100A | R100A | 298.15 | 7 | -0.693 | -0.911327081 | 0.916439905 |
| 1GUA | RB59A | R59A | 298.15 | 7 | 0.178 | -0.835326749 | 0.839461734 |
| 1GUA | RB89L | R89L | 298.15 | 7 | 1.078 | 0.100607913 | -0.103217167 |
| 1GUA | SB77A | S77A | 298.15 | 7 | -1.573 | -0.403853342 | 0.40439551 |
| 1GUA | SB77T | S77T | 298.15 | 7 | 0.581 | 0.321307341 | -0.322427727 |
| 1GUA | TB68A | T68A | 298.15 | 7 | -0.109 | -0.707743918 | 0.679598091 |
| 1GUA | VB128A | V128A | 298.15 | 7 | -3.368 | -1.656522614 | 1.6740647 |
| 1GUA | VB60A | V60A | 298.15 | 7 | -2.251 | -1.482598587 | 1.504219847 |
| 1GUA | VB69A | V69A | 298.15 | 7 | -1.116 | -1.436126019 | 1.430482732 |
| 1GUA | VB70A | V70A | 298.15 | 7 | -1.77 | -1.4922487 | 1.477651166 |
| 1GUA | VB72A | V72A | 298.15 | 7 | -1.052 | -0.883970065 | 0.883396035 |
| 1GUA | VB72I | V72I | 298.15 | 7 | -0.864 | 0.292431482 | -0.290945051 |
| 1GUA | VB98A | V98A | 298.15 | 7 | -1.981 | -1.631886292 | 1.638284742 |
| 1GWY | AA8D | A8D |  | 7.4 | -1.6 | 0.622497891 | -0.623030874 |
| 1H0X | PA18A | P18A | 298.15 | 7 | -1.936 | -0.525314129 | 0.525195632 |
| 1H0X | PA62A | P62A | 298.15 | 7 | -2.581 | -0.514002756 | 0.514271547 |
| 1HCQ | CA67A | C67A | 298.15 | 6.5 | -2.09 | -0.500308258 | 0.507356365 |
| 1HCQ | CA67S | C67S | 298.15 | 6.5 | -1.99 | -0.501831568 | 0.492915376 |
| 1IOJ | GA15A | G15A | 323.15 | 7.6 | 0.4 | 0.144859367 | -0.150203 |
| 1ITM | CA24T | C24T | 298.15 | 6 | -3.3 | -0.043913614 | 0.040275479 |
| 1ITM | CA3T | C3T | 298.15 | 6 | -5 | -0.050079278 | 0.050238482 |
| 1ITM | WA91S | W91S | 298.15 | 7 | 1.4 | -0.226971611 | 0.143914491 |
| 1IV7 | AB173C | A173C | 298.15 | 8 | 0.5 | 1.201500843 | -1.188889569 |
| 1IV7 | FB189C | F189C | 298.15 | 8 | -2.5 | -0.241386493 | 0.27381029 |
| 1IV7 | LB162C | L162C | 298.15 | 8 | -1.9 | 0.296037428 | -0.301866578 |
| 1IV9 | SA76Y | S76Y | 298.15 | 5.5 | 4.299 | 0.461327398 | -0.476522253 |
| 1J8I | KA25A | K25A | 298.15 | 6 | 0.1 | -0.635655561 | 0.623229447 |
| 1J8I | KA66A | K66A | 298.15 | 6 | 0.1 | -0.770207088 | 0.789240818 |
| 1J8I | RA23A | R23A | 298.15 | 6 | 0.8 | -0.816223824 | 0.805951145 |
| 1J8I | RA35A | R35A | 298.15 | 6 | -0.1 | -1.114112617 | 1.103751648 |
| 1J8I | RA43A | R43A | 298.15 | 6 | 0.7 | -0.446900115 | 0.457898467 |
| 1J8I | RA9A | R9A | 298.15 | 6 | -0.1 | -1.611807717 | 1.542598482 |
| 1JL9 | AA25C | A25C | 295.15 | 8.4 | -1.44 | 0.560850458 | -0.553138873 |
| 1JLV | EA64D | E64D | 315.15 | 6.5 | -1.162 | 0.09609844 | -0.101665514 |
| 1JLV | EA64N | E64N | 315.15 | 6.5 | -2.304 | -0.332498006 | 0.337585667 |
| 1JLV | EA64Q | E64Q | 315.15 | 6.5 | -2.302 | 0.024847689 | -0.024226715 |
| 1L6H | FA39A | F39A | 298.15 | 7 | -1.697 | -0.798490501 | 0.790888928 |
| 1L6H | YA45A | Y45A | 298.15 | 7 | -0.55 | -1.362513836 | 1.356752272 |
| 1L6H | YA48A | Y48A | 298.15 | 7 | -1.482 | -0.710112318 | 0.728864339 |
| 1LVM | EA106G | E106G | 298.15 | 8 | 1.4 | -0.40304908 | 0.401954252 |
| 1LVM | KA45F | K45F | 298.15 | 8 | 0.43 | 0.348729853 | -0.353854121 |
| 1LVM | LA56V | L56V | 298.15 | 8 | 0 | -0.205490237 | 0.199897314 |
| 1LVM | QA58F | Q58F | 298.15 | 8 | 0.6 | 1.003254632 | -1.003215631 |
| 1LVM | SA135G | S135G | 298.15 | 8 | 0 | -0.070722048 | 0.056581402 |
| 1N18 | EA100G | E100G | 298.15 | 7.8 | -2.5 | -1.140051882 | 1.162700166 |
| 1N18 | GA85R | G85R | 298.15 | 7.8 | -1.4 | 1.732012844 | -1.744211652 |
| 1N18 | HA46R | H46R | 298.15 | 7.8 | 1.4 | 0.485887009 | -0.490109761 |
| 1N18 | VA148I | V148I | 298.15 | 7.8 | 0.1 | 0.344069266 | -0.336735158 |
| 1N88 | FA21A | F21A | 298.15 | 6.3 | -1.39 | -1.448605957 | 1.444667437 |
| 1N88 | IA39A | I39A | 298.15 | 6.3 | -1.39 | -1.812839117 | 1.826917374 |
| 1N88 | IA80A | I80A | 298.15 | 6.3 | -2.79 | -2.219635279 | 2.220577006 |
| 1N88 | IA89A | I89A | 298.15 | 6.3 | -2.35 | -1.807090829 | 1.820827022 |
| 1N88 | IA8V | I8V | 298.15 | 6.3 | -1.52 | -0.268148281 | 0.268725974 |
| 1N88 | LA13A | L13A | 298.15 | 6.3 | -1.52 | -1.495632961 | 1.493628796 |
| 1N88 | LA57A | L57A | 298.15 | 6.3 | -1.11 | -1.891945462 | 1.899051721 |
| 1N88 | VA12A | V12A | 298.15 | 6.3 | -1.94 | -1.001991435 | 1.003383273 |
| 1N88 | VA30A | V30A | 298.15 | 6.3 | -2.84 | -0.77369814 | 0.777202129 |
| 1N88 | VA43A | V43A | 298.15 | 6.3 | -2.82 | -1.198012804 | 1.175931752 |
| 1N88 | VA49A | V49A | 298.15 | 6.3 | -2.83 | -0.728218114 | 0.734173549 |
| 1N88 | VA51A | V51A | 298.15 | 6.3 | -3.24 | -0.934673545 | 0.96854231 |
| 1N88 | VA54A | V54A | 298.15 | 6.3 | -3.73 | -1.364726987 | 1.38060418 |
| 1N88 | VA81A | V81A | 298.15 | 6.3 | -2.82 | -1.438823159 | 1.437751275 |
| 1N88 | VA83A | V83A | 298.15 | 6.3 | -3.1 | -1.279083542 | 1.276450057 |
| 1N88 | YA18A | Y18A | 298.15 | 6.3 | 0.06 | -0.938020354 | 0.958269381 |
| 1N88 | YA26A | Y26A | 298.15 | 6.3 | -4.61 | -2.47297428 | 2.477672339 |
| 1NM1 | DA187N | D187N | 298.15 | 7.2 | -0.88 | -0.136269609 | 0.1362827 |
| 1NM1 | DA187Y | D187Y | 288.15 | 7.2 | -1.9 | 0.172995366 | -0.175684786 |
| 1O1U | EA110A | E110A | 298.15 | 7.2 | -3.3 | 0.024791422 | -0.024604328 |
| 1O1U | NA61A | N61A | 298.15 | 7.2 | -1.2 | -0.571311297 | 0.549094218 |
| 1O1U | QA51A | Q51A | 298.15 | 7.2 | -0.3 | -0.383792227 | 0.388685087 |
| 1O1U | QA99A | Q99A | 298.15 | 7.2 | 0.3 | -0.240035551 | 0.215054038 |
| 1O1U | TA38A | T38A | 298.15 | 7.2 | -1.2 | -0.41617745 | 0.406790196 |
| 1O1U | WA49Y | W49Y | 298.15 | 7.2 | -1.2 | 0.165580142 | -0.159324212 |
| 1O6X | AA31G | A31G | 298.15 | 7 | -1.2 | 0.510093597 | -0.525314084 |
| 1O6X | AA50G | A50G | 298.15 | 7 | -1.4 | -0.335048758 | 0.340794501 |
| 1O6X | DA38A | D38A | 298.15 | 7 | 0.36 | -0.080158975 | 0.062626156 |
| 1O6X | EA14A | E14A | 298.15 | 7 | -0.3 | -0.631072634 | 0.639849118 |
| 1O6X | EA20G | E20G | 298.15 | 7 | -1.42 | 0.237158595 | -0.209544861 |
| 1O6X | FA39L | F39L | 298.15 | 7 | -1.88 | -0.605106198 | 0.605984198 |
| 1O6X | FA65A | F65A | 298.15 | 7 | -0.98 | -0.792915541 | 0.802100649 |
| 1O6X | HA51A | H51A | 298.15 | 7 | -0.61 | -1.037145883 | 1.025806593 |
| 1O6X | IA15V | I15V | 298.15 | 7 | -0.43 | -0.035059778 | 0.06179169 |
| 1O6X | IA23V | I23V | 298.15 | 7 | 0.42 | 0.303770811 | -0.315735749 |
| 1O6X | IA71V | I71V | 298.15 | 7 | -1.31 | -0.34657577 | 0.349920379 |
| 1O6X | IA75A | I75A | 298.15 | 7 | -1.31 | -1.401004602 | 1.392081138 |
| 1O6X | KA41A | K41A | 298.15 | 7 | -1.98 | -0.263821195 | 0.26002022 |
| 1O6X | LA26V | L26V | 298.15 | 7 | -0.97 | 0.116458674 | -0.095036665 |
| 1O6X | NA58A | N58A | 298.15 | 7 | -0.08 | 0.153040493 | -0.163912579 |
| 1O6X | QA60G | Q60G | 298.15 | 7 | -0.49 | -0.467488388 | 0.479600798 |
| 1O6X | VA12A | V12A | 298.15 | 7 | -1.1 | -0.810204944 | 0.795204777 |
| 1O6X | VA52A | V52A | 298.15 | 7 | -1.08 | -0.468350898 | 0.472312708 |
| 1O6X | VA64G | V64G | 298.15 | 7 | -1.1 | -0.264543235 | 0.273537066 |
| 1O6X | YA73L | Y73L | 298.15 | 7 | -2.78 | 0.07672119 | -0.07940594 |
| 1OSI | IA11V | I11V | 313.15 | 7 | -0.05 | -0.158378982 | 0.147123363 |
| 1OSI | SA92F | S92F | 313.15 | 7 | 0.39 | 0.664246643 | -0.692932793 |
| 1OSI | VA126M | V126M | 313.15 | 7 | -2.25 | -0.007011316 | -0.00081866 |
| 1OSI | VA15I | V15I | 313.15 | 7 | -0.78 | 0.322831923 | -0.33218361 |
| 1PFL | AA20T | A20T | 298.15 | 7.4 | -1.53 | 0.388697737 | -0.389064885 |
| 1PFL | GA117V | G117V | 298.15 | 7.4 | -1.96 | 0.32641411 | -0.316942245 |
| 1PFL | MA113T | M113T | 298.15 | 7 | -3.53 | -0.321506918 | 0.299038367 |
| 1PFL | QA138L | Q138L | 298.15 | 7.4 | 1.82 | -0.212250161 | 0.176429927 |
| 1PFL | TA108M | T108M | 298.15 | 7.4 | 0.07 | 0.852740803 | -0.872894352 |
| 1PRE | CA159S | C159S | 298.15 | 7.4 | -2.4 | -0.058939621 | 0.055293988 |
| 1PRG | EA460K | E460K | 283.15 | 8 | -0.17 | 0.094577329 | -0.096771137 |
| 1PRG | FA360L | F360L | 283.15 | 8 | -0.4 | 0.182478681 | -0.179723212 |
| 1PRG | PA467L | P467L | 283.15 | 8 | 0.11 | 1.68151566 | -1.6785117 |
| 1PRG | QA286P | Q286P | 283.15 | 8 | -0.3 | -1.039020243 | 1.042471884 |
| 1PRG | RA288H | R288H | 283.15 | 8 | 0.06 | -0.053749523 | 0.053072597 |
| 1PRG | RA357A | R357A | 283.15 | 8 | 0.19 | -1.095642076 | 1.076733507 |
| 1PRG | VA290M | V290M | 283.15 | 8 | 0.03 | 0.097108777 | -0.102330421 |
| 1R2Y | RA244E | R244E | 310.15 | 6.8 | -4.2 | -0.175172723 | 0.164334857 |
| 1R6R | IA88N | I88N | 298.15 | 6 | -0.13 | -0.503424575 | 0.502243191 |
| 1R6R | LA50S | L50S | 298.15 | 6 | -2.81 | -0.261460553 | 0.318143524 |
| 1R6R | LA54S | L54S | 298.15 | 6 | -0.24 | -0.307043635 | 0.386256898 |
| 1R6R | LA81N | L81N | 298.15 | 6 | -6.84 | -0.265553002 | 0.282154764 |
| 1SPD | CA111S | C111S | 348.05 | 7.8 | 0.8 | -0.486183771 | 0.486160628 |
| 1SPD | CA6A | C6A | 348.05 | 7.8 | 0.1 | -0.933571284 | 0.938050284 |
| 1X0J | DA160N | D160N | 283.15 | 7.5 | -4.1 | -0.352689446 | 0.35758896 |
| 1X0J | DA160Y | D160Y | 283.15 | 7.5 | -4.02 | 0.397848342 | -0.412260244 |
| 1X0J | DA161N | D161N | 283.15 | 7.5 | -2.21 | -0.23333283 | 0.238807213 |
| 1X0J | DA161Y | D161Y | 283.15 | 7.5 | -3.38 | 0.286355383 | -0.281377321 |
| 1X0J | EA140K | E140K | 283.15 | 7.5 | -3.48 | 0.200320429 | -0.203263917 |
| 1X0J | RA100L | R100L | 283.15 | 7.5 | -5.22 | 0.405641943 | -0.403161784 |
| 1X0J | YA153H | Y153H | 283.15 | 7.5 | -6.49 | -0.567663858 | 0.551881102 |
| 1XWS | EA124Q | E124Q | 283.15 | 7.5 | -2.9 | -0.167148877 | 0.16916471 |
| 1XWS | EA135K | E135K | 283.15 | 7.5 | -4.59 | 0.165180994 | -0.167567335 |
| 1XWS | EA142D | E142D | 283.15 | 7.5 | -3.22 | -0.060591002 | 0.066910731 |
| 1XWS | YA53H | Y53H | 283.15 | 7.5 | -3.3 | -0.51686049 | 0.516831887 |
| 1XXN | NA181R | N181R | 328.15 | 6.5 | 0.358 | 0.550485477 | -0.561566866 |
| 1XXN | NA32D | N32D | 328.15 | 6.5 | -0.86 | 0.06705999 | -0.109363983 |
| 1XXN | NA54E | N54E | 328.15 | 6.5 | -0.645 | 0.15292551 | -0.154850491 |
| 1XXN | SA22E | S22E | 328.15 | 6.5 | 0.239 | 0.337017387 | -0.338625966 |
| 1XXN | SA27E | S27E | 328.15 | 6.5 | -0.645 | 0.352987674 | -0.357751413 |
| 1XZO | FA42W | F42W | 298.15 | 7 | 2.87 | -0.005491363 | 0.000762057 |
| 1XZO | WA101F | W101F | 298.15 | 7 | -2.89 | -0.158335501 | 0.155870499 |
| 1XZO | WA36A | W36A | 298.15 | 7 | -8.93 | -1.915376168 | 1.928624998 |
| 1XZO | WA36F | W36F | 298.15 | 7 | -4.25 | -0.504331106 | 0.509315354 |
| 2ARF | HA1069Q | H1069Q | 293.15 | 7 | -0.717 | -0.076669842 | 0.070681703 |
| 2BJD | VA84D | V84D | 298.15 | 5.5 | -4.493 | -0.602852625 | 0.621434119 |
| 2BJD | VA84P | V84P | 298.15 | 5.5 | -4.517 | -0.705550374 | 0.730930768 |
| 2BJD | YA86E | Y86E | 298.15 | 5.5 | -2.103 | -0.616796182 | 0.613536805 |
| 2C9Q | YA79F | Y79F | 308.15 | 7.4 | -0.012 | -0.210997922 | 0.206246608 |
| 2C9Q | YA79W | Y79W | 308.15 | 7.4 | -1.233 | 0.072151858 | -0.076649091 |
| 2CLR | HB13F | H13F | 298.15 | 7.4 | 0.12 | 0.346286854 | -0.339683777 |
| 2CLR | HB31F | H31F | 298.15 | 7.4 | 2.605 | 0.578139857 | -0.579519276 |
| 2CLR | HB51F | H51F | 298.15 | 7.4 | -1.912 | 0.633045759 | -0.625290576 |
| 2CLR | HB84A | H84A | 298.15 | 7.4 | -2.629 | -0.741024797 | 0.736119567 |
| 2DVV | QA443H | Q443H | 283.15 | 7.5 | 0.89 | -0.230788792 | 0.222654918 |
| 2DVV | RA419W | R419W | 283.15 | 7.5 | -0.02 | -0.213446912 | 0.208417186 |
| 2H3F | EA12A | E12A | 298.15 | 7 | 1.07 | -0.415430008 | 0.34263369 |
| 2HBB | AA22G | A22G | 298.15 | 5.5 | -0.32 | -0.589763015 | 0.590214611 |
| 2HBB | AA26G | A26G | 298.15 | 5.5 | -1.79 | -0.493190181 | 0.483133125 |
| 2HBB | AA36G | A36G | 298.15 | 5.5 | -1.55 | -0.805473098 | 0.798179343 |
| 2HBB | AA39G | A39G | 298.15 | 5.5 | -1.59 | 0.253545276 | -0.251483725 |
| 2HBB | AA42G | A42G | 298.15 | 5.5 | -0.33 | -0.56666372 | 0.54664361 |
| 2HBB | AA46G | A46G | 298.15 | 5.5 | -0.55 | -0.127468749 | 0.118651873 |
| 2HBB | AA49G | A49G | 298.15 | 5.5 | -0.22 | -0.462858074 | 0.464233868 |
| 2HBB | DA23A | D23A | 298.15 | 5.5 | -0.03 | -0.652591138 | 0.64989416 |
| 2HBB | DA23N | D23N | 298.15 | 5.5 | -0.79 | -0.162131294 | 0.16160671 |
| 2HBB | DA8A | D8A | 298.15 | 5.5 | -1.3 | -0.479051566 | 0.479896478 |
| 2HBB | DA8N | D8N | 298.15 | 5.5 | -0.38 | -0.020331823 | 0.028421589 |
| 2HBB | EA17A | E17A | 298.15 | 5.5 | -1.69 | -0.417813898 | 0.417316815 |
| 2HBB | EA17Q | E17Q | 298.15 | 5.5 | -0.33 | -0.141057968 | 0.140078913 |
| 2HBB | EA38A | E38A | 298.15 | 5.5 | -0.41 | -0.592029402 | 0.595647645 |
| 2HBB | EA38Q | E38Q | 298.15 | 5.5 | -0.68 | -0.152440084 | 0.152176406 |
| 2HBB | EA48Q | E48Q | 298.15 | 5.5 | 0.07 | -0.199055514 | 0.197863801 |
| 2HBB | FA31A | F31A | 298.15 | 5.5 | -2.84 | -1.007982643 | 1.0035015 |
| 2HBB | FA5L | F5L | 298.15 | 5.5 | -1.15 | -0.09675647 | 0.097122327 |
| 2HBB | GA24A | G24A | 298.15 | 5.5 | 0.02 | -0.022490772 | 0.012844457 |
| 2HBB | GA34A | G34A | 298.15 | 5.5 | -1.02 | 0.015875434 | -0.032016784 |
| 2HBB | IA18A | I18A | 298.15 | 5.5 | -2.05 | -1.712166168 | 1.712857916 |
| 2HBB | IA18V | I18V | 298.15 | 5.5 | -0.07 | -0.27527114 | 0.272526831 |
| 2HBB | IA37A | I37A | 298.15 | 5.5 | -1.28 | -1.982300396 | 1.979921655 |
| 2HBB | IA37V | I37V | 298.15 | 5.5 | 0.09 | -0.338339732 | 0.343185632 |
| 2HBB | IA4V | I4V | 298.15 | 5.5 | -0.59 | -0.310627451 | 0.310776787 |
| 2HBB | KA10E | K10E | 298.15 | 5.5 | 0.13 | 0.036874122 | -0.038432541 |
| 2HBB | KA10M | K10M | 298.15 | 5.5 | 0.47 | 0.424179949 | -0.428629464 |
| 2HBB | KA12A | K12A | 298.15 | 5.5 | 0.61 | -0.358277227 | 0.35955956 |
| 2HBB | KA12G | K12G | 298.15 | 5.5 | 0.03 | -0.506833833 | 0.502441012 |
| 2HBB | KA12M | K12M | 298.15 | 5.5 | 1.8 | 0.138273405 | -0.145789155 |
| 2HBB | KA14A | K14A | 298.15 | 5.5 | -0.73 | -0.560740924 | 0.557541744 |
| 2HBB | KA14G | K14G | 298.15 | 5.5 | -1.3 | -0.769679173 | 0.768807854 |
| 2HBB | KA14M | K14M | 298.15 | 5.5 | -0.34 | 0.468392033 | -0.486467826 |
| 2HBB | KA15G | K15G | 298.15 | 5.5 | -1.74 | -0.385550297 | 0.391212833 |
| 2HBB | KA15M | K15M | 298.15 | 5.5 | -0.44 | 0.208428501 | -0.202724786 |
| 2HBB | KA7M | K7M | 298.15 | 5.5 | 0.21 | 0.17375698 | -0.168358069 |
| 2HBB | LA30A | L30A | 298.15 | 5.5 | -2.76 | -1.569632422 | 1.557103465 |
| 2HBB | LA35A | L35A | 298.15 | 5.5 | -1.21 | -1.006969961 | 1.021038761 |
| 2HBB | LA44A | L44A | 298.15 | 5.5 | -1.73 | -1.49214328 | 1.487523884 |
| 2HBB | LA47A | L47A | 298.15 | 5.5 | -0.84 | -1.52067426 | 1.517255972 |
| 2HBB | LA6A | L6A | 298.15 | 5.5 | -3.1 | -1.310411192 | 1.311067291 |
| 2HBB | MA1A | M1A | 298.15 | 5.5 | -1.46 | -1.191093387 | 1.192378508 |
| 2HBB | TA40S | T40S | 298.15 | 5.5 | -0.8 | -0.250969761 | 0.251178059 |
| 2HBB | VA21A | V21A | 298.15 | 5.5 | -2.33 | -0.889689899 | 0.887625779 |
| 2HBB | VA3A | V3A | 298.15 | 5.5 | -2.55 | -1.30294164 | 1.303278652 |
| 2HBB | VA9A | V9A | 298.15 | 5.5 | -1.81 | -0.998948897 | 0.990751826 |
| 2JIE | CA170A | C170A | 298.15 | 7.5 | 2.997 | -0.814840285 | 0.818092944 |
| 2JIE | CA170Q | C170Q | 298.15 | 7.5 | 2.581 | -0.358426277 | 0.372420256 |
| 2JIE | EA157D | E157D | 298.15 | 7.5 | -2.075 | -0.167936333 | 0.166835343 |
| 2JIE | EA167A | E167A | 298.15 | 7.5 | 4.247 | -0.669199358 | 0.678516104 |
| 2JIE | EA225A | E225A | 298.15 | 7.5 | -4.36 | -0.529964994 | 0.525406721 |
| 2JIE | EA225H | E225H | 298.15 | 7.5 | -1.515 | -0.066334705 | 0.066648101 |
| 2JIE | EA409A | E409A | 298.15 | 7.5 | 0.825 | -0.583659976 | 0.577839311 |
| 2JIE | EA409D | E409D | 298.15 | 7.5 | -0.909 | -0.144085749 | 0.151150503 |
| 2JIE | EA426S | E426S | 298.15 | 7.5 | 0.87 | -0.353743988 | 0.358330174 |
| 2JIE | FA418A | F418A | 298.15 | 7.5 | -0.165 | -1.475136148 | 1.468690182 |
| 2JIE | HA104R | H104R | 298.15 | 7.5 | 1.536 | 0.16869652 | -0.192698305 |
| 2JIE | IA247E | I247E | 298.15 | 7.5 | 1.241 | -1.151745338 | 1.142031731 |
| 2JIE | IA247N | I247N | 298.15 | 7.5 | 0.974 | -1.114020097 | 1.119890412 |
| 2JIE | LA174A | L174A | 298.15 | 7.5 | -0.79 | -1.388170583 | 1.398315176 |
| 2JIE | LA174R | L174R | 298.15 | 7.5 | 0.336 | 0.127772056 | -0.134197547 |
| 2JIE | MA224A | M224A | 298.15 | 7.5 | -4.098 | -1.659630858 | 1.649602235 |
| 2JIE | MA326A | M326A | 298.15 | 7.5 | -1.298 | -1.267160424 | 1.265162465 |
| 2JIE | NA166A | N166A | 298.15 | 7.5 | -3.397 | -0.708164482 | 0.713201281 |
| 2JIE | NA166C | N166C | 298.15 | 7.5 | -2.964 | 0.282948122 | -0.258998371 |
| 2JIE | NA223A | N223A | 298.15 | 7.5 | -0.671 | -0.104576109 | 0.106632122 |
| 2JIE | NA223G | N223G | 298.15 | 7.5 | 3.462 | -0.674628116 | 0.677865864 |
| 2JIE | NA223H | N223H | 298.15 | 7.5 | 2.583 | 0.228958592 | -0.240695463 |
| 2JIE | NA223R | N223R | 298.15 | 7.5 | 3.976 | 0.522468082 | -0.541364731 |
| 2JIE | NA223Y | N223Y | 298.15 | 7.5 | 5.73 | 0.693343231 | -0.715236672 |
| 2JIE | NA296A | N296A | 298.15 | 7.5 | -2.25 | -0.775129664 | 0.789669465 |
| 2JIE | NA296C | N296C | 298.15 | 7.5 | -0.017 | 0.184390623 | -0.19994241 |
| 2JIE | NA407A | N407A | 298.15 | 7.5 | 0.464 | -0.630692862 | 0.625971198 |
| 2JIE | NA407C | N407C | 298.15 | 7.5 | 0.143 | 0.473656354 | -0.472339242 |
| 2JIE | QA22S | Q22S | 298.15 | 7.5 | -0.209 | -0.411047559 | 0.431305535 |
| 2JIE | RA243A | R243A | 298.15 | 7.5 | -0.166 | -0.613724527 | 0.62718322 |
| 2JIE | RA243D | R243D | 298.15 | 7.5 | 0.213 | -0.406961606 | 0.406346792 |
| 2JIE | RA243K | R243K | 298.15 | 7.5 | -1.463 | -0.284151057 | 0.289648562 |
| 2JIE | SA17A | S17A | 298.15 | 7.5 | -1.246 | -0.405420926 | 0.40288777 |
| 2JIE | SA19A | S19A | 298.15 | 7.5 | 0.951 | -0.304191652 | 0.300658214 |
| 2JIE | SA334A | S334A | 298.15 | 7.5 | -1.454 | -0.271993684 | 0.274348873 |
| 2JIE | SA403A | S403A | 298.15 | 7.5 | 0.92 | -0.296082329 | 0.295517474 |
| 2JIE | TA18A | T18A | 298.15 | 7.5 | -0.994 | -0.432502503 | 0.445864531 |
| 2JIE | TA221A | T221A | 298.15 | 7.5 | 3.189 | -0.42872245 | 0.41866533 |
| 2JIE | TA355A | T355A | 298.15 | 7.5 | -2.961 | -0.551665297 | 0.549524694 |
| 2JIE | WA123F | W123F | 298.15 | 7.5 | 2.19 | 0.352801213 | -0.362555776 |
| 2JIE | WA123H | W123H | 298.15 | 7.5 | 1.06 | -0.440251243 | 0.423886782 |
| 2JIE | WA328A | W328A | 298.15 | 7.5 | -1.159 | -0.910835981 | 0.912321491 |
| 2JIE | WA328C | W328C | 298.15 | 7.5 | -2.664 | -0.13938327 | 0.146168062 |
| 2JIE | WA328H | W328H | 298.15 | 7.5 | -2.573 | -0.36033377 | 0.371454275 |
| 2JIE | WA328R | W328R | 298.15 | 7.5 | -0.95 | -0.417838806 | 0.440454868 |
| 2JIE | WA402C | W402C | 298.15 | 7.5 | -0.36 | -0.323991561 | 0.311476034 |
| 2JIE | WA402R | W402R | 298.15 | 7.5 | 2.615 | -0.375214509 | 0.363038713 |
| 2JIE | WA412Y | W412Y | 298.15 | 7.5 | 0.599 | 0.076028486 | -0.078864674 |
| 2JIE | YA21A | Y21A | 298.15 | 7.5 | -3.443 | -1.309481904 | 1.314239667 |
| 2JIE | YA297A | Y297A | 298.15 | 7.5 | 1.072 | -1.806899208 | 1.799243429 |
| 2JIE | YA297F | Y297F | 298.15 | 7.5 | -1.853 | -0.168895716 | 0.18056999 |
| 2JUC | CA57D | C57D | 298.15 | 5.7 | -2.54 | -0.197860161 | 0.206145719 |
| 2JUC | CA57S | C57S | 298.15 | 5.7 | -0.71 | -0.260923532 | 0.256017423 |
| 2JUC | RA18A | R18A | 298.15 | 5.7 | -1.75 | -0.646975344 | 0.691529745 |
| 2KJ3 | DA288A | D288A | 298.15 | 7 | 1.2 | -0.014591834 | 0.021456992 |
| 2KJ3 | EA272A | E272A | 298.15 | 7 | 1.9 | -0.246993952 | 0.224497876 |
| 2KJ3 | GA278A | G278A | 298.15 | 7 | -0.8 | 0.71645152 | -0.745215716 |
| 2KJ3 | KA229A | K229A | 298.15 | 7 | 1.5 | -0.502200325 | 0.499945004 |
| 2KJ3 | LA241A | L241A | 298.15 | 7 | 1 | -0.976837734 | 1.080176799 |
| 2KJ3 | SA273A | S273A | 298.15 | 7 | 1.4 | 0.133509986 | -0.124080613 |
| 2KJ3 | VA239A | V239A | 298.15 | 7 | 1 | -0.52588813 | 0.55750731 |
| 2KJ3 | VA264A | V264A | 298.15 | 7 | 1 | -0.771551316 | 0.76581607 |
| 2KJ3 | VA267A | V267A | 298.15 | 7 | 0.2 | -0.477650358 | 0.48180718 |
| 2KS4 | DA9A | D9A |  | 7.4 | 1.26 | -0.293025172 | 0.280153756 |
| 2KS4 | EA2A | E2A |  | 7.4 | -0.23 | -0.885050526 | 0.86784044 |
| 2LTB | LA558P | L558P | 298.15 | 7.4 | -1.57 | -1.379983818 | 1.402767855 |
| 2LTB | LA569R | L569R | 298.15 | 7.4 | -3.11 | 0.121058709 | -0.104627545 |
| 2LTB | MA619K | M619K | 298.15 | 7.4 | -3.34 | 1.037960977 | -1.058591497 |
| 2LTB | RA533Q | R533Q | 298.15 | 7.4 | -1.1 | -0.045144168 | 0.041172982 |
| 2LTB | RA555Q | R555Q | 298.15 | 7.4 | -0.36 | -0.097437356 | 0.096600313 |
| 2LTB | RA555W | R555W | 298.15 | 7.4 | 1.79 | 0.341247797 | -0.341436791 |
| 2M5S | DA302A | D302A | 293.15 | 7.6 | -4 | -0.309261039 | 0.424304307 |
| 2M5S | HA305A | H305A | 293.15 | 7.6 | -2 | -0.433759863 | 0.589444862 |
| 2MPC | AA89T | A89T | 298.15 | 7.5 | -0.24 | 0.398130297 | -0.396877903 |
| 2MPC | RA42A | R42A | 298.15 | 7.5 | 0.62 | -0.567057722 | 0.574148384 |
| 2MPC | RA42W | R42W | 298.15 | 7.5 | 2.08 | -0.027956803 | 0.02415811 |
| 2N7Z | PA88A | P88A | 298.15 | 8 | -0.6 | 0.381045546 | -0.345556589 |
| 2NTE | IA738V | I738V | 283.15 | 7.5 | -2 | -0.391471988 | 0.394571741 |
| 2NTE | RA658C | R658C | 283.15 | 7.5 | -2.3 | 0.181761194 | -0.173925218 |
| 2OUO | AA420D | A420D | 283.15 | 7.5 | 0.44 | 0.575461543 | -0.580093264 |
| 2PR5 | AA33Y | A33Y | 298.15 | 7.9 | 0.5 | 0.757265315 | -0.780399959 |
| 2PR5 | AA81M | A81M | 298.15 | 7.9 | -0.4 | 1.426443133 | -1.432426702 |
| 2PR5 | DA109E | D109E | 298.15 | 7.9 | 0.3 | -0.05314809 | 0.050938713 |
| 2PR5 | HA22K | H22K | 298.15 | 7.9 | 0.3 | 0.138506521 | -0.134566711 |
| 2PR5 | HA22W | H22W | 298.15 | 7.9 | 1.2 | 0.119370236 | -0.114067035 |
| 2PR5 | MA111F | M111F | 298.15 | 7.9 | 2.9 | -0.351201134 | 0.355963746 |
| 2PR5 | NA107F | N107F | 298.15 | 7.9 | 0.9 | 0.705149166 | -0.703196542 |
| 2PR5 | NA107Y | N107Y | 298.15 | 7.9 | 0.8 | 0.98356332 | -0.973909468 |
| 2PR5 | NA124F | N124F | 298.15 | 7.9 | 1.1 | 0.794682013 | -0.792303888 |
| 2PR5 | NA124Y | N124Y | 298.15 | 7.9 | 2.6 | 0.979017 | -0.96877313 |
| 2PR5 | TA30M | T30M | 298.15 | 7.9 | 0.5 | 1.244174244 | -1.246538623 |
| 2PR5 | TA50M | T50M | 298.15 | 7.9 | -0.5 | 1.08136379 | -1.103149395 |
| 2PR5 | TA54Y | T54Y | 298.15 | 7.9 | 0.2 | 0.691683846 | -0.667261789 |
| 2PR5 | VA120I | V120I | 298.15 | 7.9 | 1.4 | 0.360737256 | -0.360242759 |
| 2PR5 | VA25I | V25I | 298.15 | 7.9 | 0.4 | 0.2626768 | -0.26686818 |
| 2PR5 | VA88L | V88L | 298.15 | 7.9 | -0.2 | 0.397275795 | -0.388754897 |
| 2PR5 | VA90I | V90I | 298.15 | 7.9 | -0.7 | 0.431686561 | -0.415188031 |
| 2PTL | AA22G | A22G | 295.15 | 7 | -2.43 | -0.185897171 | 0.228131344 |
| 2PTL | AA27P | A27P | 295.15 | 7 | 0.1 | 0.412503727 | -0.419089194 |
| 2PTL | AA27V | A27V | 295.15 | 7 | -0.83 | 0.590070257 | -0.582072904 |
| 2PTL | AA34G | A34G | 295.15 | 7 | -2.17 | 0.67965987 | -0.677554287 |
| 2PTL | AA34V | A34V | 295.15 | 7 | 1.47 | 0.861773767 | -0.879421487 |
| 2PTL | AA43G | A43G | 295.15 | 7 | -2.54 | 0.290036744 | -0.290529001 |
| 2PTL | AA47G | A47G | 295.15 | 7 | -3.1 | -0.175270363 | 0.193098916 |
| 2PTL | AA49G | A49G | 295.15 | 7 | -1.32 | -0.188657359 | 0.172823937 |
| 2PTL | AA51G | A51G | 295.15 | 7 | -3.12 | -0.246111481 | 0.255787045 |
| 2PTL | AA66G | A66G | 295.15 | 7 | -0.49 | -0.229198345 | 0.213278024 |
| 2PTL | DA52A | D52A | 295.15 | 7 | -1.21 | -0.575458494 | 0.588305068 |
| 2PTL | DA52G | D52G | 295.15 | 7 | -2.14 | -0.429717614 | 0.440149104 |
| 2PTL | DA64A | D64A | 295.15 | 7 | -0.2 | -0.565527036 | 0.569181004 |
| 2PTL | EA35A | E35A | 295.15 | 7 | -0.59 | -0.738305471 | 0.726346957 |
| 2PTL | EA46G | E46G | 295.15 | 7 | -1.19 | -0.611722352 | 0.609058363 |
| 2PTL | EA46I | E46I | 295.15 | 7 | -1.08 | 0.367033915 | -0.370426552 |
| 2PTL | EA60A | E60A | 295.15 | 7 | -0.23 | -0.95662383 | 0.919006823 |
| 2PTL | FA26A | F26A | 295.15 | 7 | -3.12 | -1.64800166 | 1.550782461 |
| 2PTL | FA26L | F26L | 295.15 | 7 | -0.68 | 1.050416646 | -1.00917734 |
| 2PTL | FA36A | F36A | 295.15 | 7 | -4.15 | -1.172604779 | 1.185413535 |
| 2PTL | FA36L | F36L | 295.15 | 7 | -3.12 | 0.385655942 | -0.384754841 |
| 2PTL | FA40G | F40G | 295.15 | 7 | -3.08 | -0.589881669 | 0.591668299 |
| 2PTL | FA40L | F40L | 295.15 | 7 | -0.38 | 0.126064474 | -0.120901676 |
| 2PTL | FA76L | F76L | 295.15 | 7 | -3.34 | 0.11405658 | -0.112296005 |
| 2PTL | FA76V | F76V | 295.15 | 7 | -3.73 | 0.060977091 | -0.058694653 |
| 2PTL | GA29A | G29A | 295.15 | 7 | -1.52 | 0.13084736 | -0.117429309 |
| 2PTL | GA29V | G29V | 295.15 | 7 | -2.53 | 0.858378343 | -0.851684171 |
| 2PTL | GA38A | G38A | 295.15 | 7 | -2.08 | 0.083905043 | -0.076671661 |
| 2PTL | GA59A | G59A | 295.15 | 7 | -2.23 | 0.329406736 | -0.33238299 |
| 2PTL | GA69A | G69A | 295.15 | 7 | -2.04 | -0.790153516 | 0.723278087 |
| 2PTL | IA20A | I20A | 295.15 | 7 | -4.9 | -1.188745021 | 1.187700383 |
| 2PTL | IA20V | I20V | 295.15 | 7 | -0.56 | -0.262261606 | 0.26784373 |
| 2PTL | IA25A | I25A | 295.15 | 7 | -1.37 | -1.288375987 | 1.30282077 |
| 2PTL | IA25V | I25V | 295.15 | 7 | -0.47 | -0.244340726 | 0.250554865 |
| 2PTL | IA74A | I74A | 295.15 | 7 | -4.72 | -1.181641556 | 1.174021285 |
| 2PTL | IA74V | I74V | 295.15 | 7 | -1.69 | -0.17597427 | 0.173276224 |
| 2PTL | KA21A | K21A | 295.15 | 7 | -0.92 | -0.673468125 | 0.681376383 |
| 2PTL | KA37A | K37A | 295.15 | 7 | -0.88 | -1.178670062 | 1.158286636 |
| 2PTL | KA42G | K42G | 295.15 | 7 | 0.16 | -1.063983047 | 1.068971509 |
| 2PTL | KA55A | K55A | 295.15 | 7 | 0.58 | -0.424873318 | 0.431974164 |
| 2PTL | KA56A | K56A | 295.15 | 7 | 0.35 | -0.475903536 | 0.469207624 |
| 2PTL | KA68A | K68A | 295.15 | 7 | -0.09 | -0.576258422 | 0.556369825 |
| 2PTL | KA75A | K75A | 295.15 | 7 | -0.45 | -0.694159231 | 0.700013386 |
| 2PTL | LA24A | L24A | 295.15 | 7 | -3.12 | -1.425308942 | 1.454383007 |
| 2PTL | LA54A | L54A | 295.15 | 7 | -2.44 | -0.99673304 | 0.984245667 |
| 2PTL | LA72A | L72A | 295.15 | 7 | -3.77 | -1.387569943 | 1.383766822 |
| 2PTL | NA23A | N23A | 295.15 | 7 | -1.87 | -1.209870743 | 1.207600844 |
| 2PTL | NA28A | N28A | 295.15 | 7 | -1.78 | -0.4549751 | 0.456129182 |
| 2PTL | NA58A | N58A | 295.15 | 7 | -0.34 | -0.35598851 | 0.328651437 |
| 2PTL | NA73A | N73A | 295.15 | 7 | -1.73 | -0.610957893 | 0.613378847 |
| 2PTL | SA30A | S30A | 295.15 | 7 | -0.3 | -0.410668167 | 0.413920841 |
| 2PTL | SA45A | S45A | 295.15 | 7 | 0.41 | 0.097978887 | -0.22701165 |
| 2PTL | SA45G | S45G | 295.15 | 7 | -0.82 | -0.168463451 | 0.168167671 |
| 2PTL | TA19A | T19A | 295.15 | 7 | -1.63 | -0.37223999 | 0.376478443 |
| 2PTL | TA31A | T31A | 295.15 | 7 | -1.17 | -0.315696263 | 0.316539509 |
| 2PTL | TA33A | T33A | 295.15 | 7 | -1.11 | -0.290351242 | 0.285965115 |
| 2PTL | TA39A | T39A | 295.15 | 7 | -1.25 | -0.313793907 | 0.320913911 |
| 2PTL | TA44A | T44A | 295.15 | 7 | -1.09 | -0.319764248 | 0.322854437 |
| 2PTL | TA53G | T53G | 295.15 | 7 | -0.17 | -0.225912466 | 0.230498263 |
| 2PTL | TA62A | T62A | 295.15 | 7 | -0.97 | -0.240681549 | 0.245344876 |
| 2PTL | TA71A | T71A | 295.15 | 7 | -1.83 | -0.344423707 | 0.350893242 |
| 2PTL | VA18A | V18A | 295.15 | 7 | -1.22 | -0.52679343 | 0.53199681 |
| 2PTL | VA63A | V63A | 295.15 | 7 | -0.92 | -0.510443661 | 0.488798848 |
| 2PTL | VA65A | V65A | 295.15 | 7 | -1.14 | -0.762429792 | 0.762470405 |
| 2PTL | YA48A | Y48A | 295.15 | 7 | -2.82 | -0.713383854 | 0.728564821 |
| 2PTL | YA50A | Y50A | 295.15 | 7 | -2.46 | -0.698914567 | 0.693619155 |
| 2PTL | YA70A | Y70A | 295.15 | 7 | -1.66 | -0.791079704 | 0.765980544 |
| 2PTL | YA70L | Y70L | 295.15 | 7 | 0.43 | -0.095321824 | 0.102631507 |
| 2RPN | EA7L | E7L | 303.15 | 8 | 1.05 | 0.316641209 | -0.321699839 |
| 2RPN | VA21K | V21K | 303.15 | 8 | 0.39 | 0.275744737 | -0.281766365 |
| 2VY0 | DA287A | D287A | 293.15 | 7 | -5.975 | -0.740833104 | 0.748932936 |
| 2VY0 | EA53A | E53A | 293.15 | 7 | -6.597 | -0.478946929 | 0.471962606 |
| 2WQG | DA15A | D15A | 322.15 | 6 | 0.3 | -0.402055687 | 0.416875969 |
| 2WQG | DA38A | D38A | 322.15 | 6 | 0.1 | -0.451739248 | 0.45345483 |
| 2WQG | DA39A | D39A | 322.15 | 6 | -2.2 | 0.034904623 | -0.022430099 |
| 2WQG | DA39N | D39N | 322.15 | 6 | -2.6 | -0.036675162 | 0.022749053 |
| 2WQG | EA40Q | E40Q | 322.15 | 6 | -0.1 | 0.162748353 | -0.166420172 |
| 2WQG | EA41G | E41G | 322.15 | 6 | -0.4 | -0.845431091 | 0.814078563 |
| 2WQG | IA36A | I36A | 322.15 | 6 | -0.9 | -0.556956992 | 0.503558511 |
| 2WQG | IA36V | I36V | 322.15 | 6 | -0.3 | 0.091933181 | -0.085749374 |
| 2WQG | KA14A | K14A | 322.15 | 6 | -0.4 | -0.666962191 | 0.778872401 |
| 2WQG | KA14G | K14G | 322.15 | 6 | -1.9 | -0.165217931 | 0.154066935 |
| 2WQG | KA19A | K19A | 322.15 | 6 | -0.3 | -1.27421533 | 1.291085756 |
| 2WQG | KA28A | K28A | 322.15 | 6 | -0.6 | -0.847754535 | 0.855944538 |
| 2WQG | LA13A | L13A | 322.15 | 6 | -0.5 | -1.257786969 | 1.262088757 |
| 2WQG | LA16A | L16A | 322.15 | 6 | -1.3 | -0.428707257 | 0.423978086 |
| 2WQG | LA8A | L8A | 322.15 | 6 | -2 | -1.03490005 | 1.057423266 |
| 2WQG | QA12A | Q12A | 322.15 | 6 | -0.6 | -0.709610865 | 0.688587129 |
| 2WQG | QA12G | Q12G | 322.15 | 6 | -2 | -0.686140235 | 0.700585766 |
| 2WQG | QA33A | Q33A | 322.15 | 6 | 0.1 | -0.485162971 | 0.395379684 |
| 2WQG | RA34A | R34A | 322.15 | 6 | -0.8 | -0.733727392 | 0.741930171 |
| 2WQG | SA23A | S23A | 322.15 | 6 | -0.6 | 0.572518998 | -0.572486231 |
| 2WQG | SA6A | S6A | 322.15 | 6 | 0.1 | -0.062125906 | 0.04552988 |
| 2WQG | SA6G | S6G | 322.15 | 6 | -0.2 | 0.015153618 | -0.054786931 |
| 2WQG | TA9S | T9S | 322.15 | 6 | 0.1 | 0.050085772 | -0.051392838 |
| 2WQG | VA10A | V10A | 322.15 | 6 | -0.1 | -0.599090264 | 0.60055028 |
| 2WQG | VA10G | V10G | 322.15 | 6 | -0.7 | -0.7207334 | 0.70413347 |
| 2WQG | VA11A | V11A | 322.15 | 6 | 0.4 | -0.54262708 | 0.543424722 |
| 2WQG | VA11G | V11G | 322.15 | 6 | 0.1 | -0.59685024 | 0.584659122 |
| 2WQG | VA24A | V24A | 322.15 | 6 | -0.5 | 0.018155588 | -0.003662554 |
| 2WQG | YA5F | Y5F | 322.15 | 6 | -2.4 | 0.01139221 | -0.033359392 |
| 3BCI | EA96Q | E96Q | 298.15 | 7 | 1.369 | -0.153697118 | 0.158028015 |
| 3BCI | TA153V | T153V | 298.15 | 7 | -1.137 | 0.645306668 | -0.621538682 |
| 3BN0 | IA20V | I20V | 295.65 | 5.5 | -1.506 | -0.361824278 | 0.364928418 |
| 3C2I | DA156E | D156E | 298.15 | 7.5 | 0.88 | 0.15260363 | -0.154994857 |
| 3C2I | FA155S | F155S | 298.15 | 7.5 | 0.43 | -0.887733348 | 0.908404526 |
| 3C2I | LA100V | L100V | 298.15 | 7.5 | 1.11 | -0.065754709 | 0.068871535 |
| 3C2I | PA152R | P152R | 298.15 | 7.5 | 1.46 | 0.61633895 | -0.603081323 |
| 3C2I | RA106Q | R106Q | 298.15 | 7.5 | 0.12 | -0.692240193 | 0.636268341 |
| 3C2I | RA106W | R106W | 298.15 | 7.5 | 0.04 | -0.560385894 | 0.526275181 |
| 3C2I | RA111G | R111G | 298.15 | 7.5 | -0.43 | -0.869573988 | 0.866316399 |
| 3C2I | RA133C | R133C | 298.15 | 7.5 | 0.37 | 0.028877341 | -0.028886528 |
| 3C2I | RA133H | R133H | 298.15 | 7.5 | 0.24 | -0.149747357 | 0.154110176 |
| 3C2I | SA134C | S134C | 298.15 | 7.5 | 1.05 | 0.389948413 | -0.383804411 |
| 3C2I | TA158A | T158A | 298.15 | 7.5 | 0.47 | -0.403686255 | 0.40273654 |
| 3C2I | TA158M | T158M | 298.15 | 7.5 | 0.25 | 0.691325346 | -0.695882173 |
| 3D3B | DA118A | D118A | 328.15 | 7.5 | -0.65 | -0.507646027 | 0.512361697 |
| 3D3B | DA118E | D118E | 328.15 | 7.5 | -0.6 | 0.191355811 | -0.189391972 |
| 3D3B | DA118K | D118K | 328.15 | 7.5 | -1.6 | 0.190536683 | -0.189063038 |
| 3D3B | DA118N | D118N | 328.15 | 7.5 | -1.34 | -0.013905696 | 0.015440644 |
| 3D3B | DA118R | D118R | 328.15 | 7.5 | -1.41 | 0.281007759 | -0.276495486 |
| 3DV0 | AI131G | A131G | 325 | 5.5 | -1.7 | -0.395478705 | 0.329219956 |
| 3DV0 | AI139G | A139G | 325 | 5.5 | -2.3 | -0.697635413 | 0.689351563 |
| 3DV0 | AI165G | A165G | 325 | 5.5 | -0.9 | -0.594928719 | 0.590799404 |
| 3DV0 | AI168G | A168G | 325 | 5.5 | -0.3 | -0.079920298 | 0.080409671 |
| 3DV0 | DI145A | D145A | 325 | 5.5 | -2.1 | -0.528947674 | 0.524371782 |
| 3DV0 | DI145N | D145N | 325 | 5.5 | -0.9 | 0.070284232 | -0.073063089 |
| 3DV0 | DI162N | D162N | 325 | 5.5 | -2 | 0.086663351 | -0.077784627 |
| 3DV0 | DI164A | D164A | 325 | 5.5 | -2.3 | -0.566199651 | 0.569462146 |
| 3DV0 | DI164G | D164G | 325 | 5.5 | -3.7 | -0.783431429 | 0.776143736 |
| 3DV0 | DI164N | D164N | 325 | 5.5 | -1.9 | 0.063726955 | -0.063859054 |
| 3DV0 | EI141A | E141A | 325 | 5.5 | -0.7 | -0.471867695 | 0.474290974 |
| 3DV0 | EI141Q | E141Q | 325 | 5.5 | -0.4 | -0.320396841 | 0.32280489 |
| 3DV0 | II130A | I130A | 325 | 5.5 | -0.7 | -1.248520049 | 1.240778969 |
| 3DV0 | II130G | I130G | 325 | 5.5 | -1.4 | -1.345851988 | 1.3466496 |
| 3DV0 | II130V | I130V | 325 | 5.5 | -0.2 | -0.224793548 | 0.225074504 |
| 3DV0 | II146A | I146A | 325 | 5.5 | -2.7 | -1.527257582 | 1.522767853 |
| 3DV0 | II146V | I146V | 325 | 5.5 | -1.2 | -0.312820568 | 0.316681295 |
| 3DV0 | II163V | I163V | 325 | 5.5 | -0.5 | -0.292276547 | 0.293154754 |
| 3DV0 | LI159A | L159A | 325 | 5.5 | -2 | -1.449503322 | 1.433179708 |
| 3DV0 | LI159G | L159G | 325 | 5.5 | -1.9 | -2.022777762 | 2.005598661 |
| 3DV0 | LI159V | L159V | 325 | 5.5 | -0.6 | -0.144371915 | 0.138475038 |
| 3DV0 | LI167A | L167A | 325 | 5.5 | -1.2 | -1.295324893 | 1.284465065 |
| 3DV0 | LI167V | L167V | 325 | 5.5 | -0.2 | -0.074859121 | 0.071511398 |
| 3DV0 | VI129A | V129A | 325 | 5.5 | -0.7 | -0.574773213 | 0.588152021 |
| 3DV0 | VI129G | V129G | 325 | 5.5 | -1 | -0.69958637 | 0.705803038 |
| 3DV0 | VI135A | V135A | 325 | 5.5 | -1.5 | -0.830647802 | 0.83217924 |
| 3DV0 | VI144A | V144A | 325 | 5.5 | -2 | -0.69237903 | 0.683683787 |
| 3DV0 | VI149A | V149A | 325 | 5.5 | -2.8 | -0.854368805 | 0.862176238 |
| 3DV0 | VI149G | V149G | 325 | 5.5 | -4.1 | -1.030431517 | 1.04500732 |
| 3DV0 | VI158A | V158A | 325 | 5.5 | -3.2 | -0.827691845 | 0.81950128 |
| 3DV0 | YI138F | Y138F | 325 | 5.5 | -2 | 0.117272472 | -0.117857843 |
| 3ECU | HA46R | H46R | 298.15 | 7.8 | 0.8 | 0.536483907 | -0.550906792 |
| 3FIS | PA61A | P61A | 331.25 | 7.4 | -0.5 | -0.453120521 | 0.457397461 |
| 3G1G | DA155Y | D155Y | 298.15 | 7 | 0.34 | 0.277174864 | -0.27708313 |
| 3G1G | FA167Y | F167Y | 298.15 | 7 | -0.74 | 0.176736425 | -0.182224067 |
| 3G1G | IA190V | I190V | 298.15 | 7 | -0.17 | -0.343186096 | 0.346898695 |
| 3G1G | RA185W | R185W | 298.15 | 7 | 1.32 | 0.19464249 | -0.193452507 |
| 3K82 | EA334L | E334L |  | 7.5 | -3.35 | 0.749567442 | -0.763375671 |
| 3K82 | EA334Q | E334Q |  | 7.5 | -1.68 | -0.212368918 | 0.209960007 |
| 3K82 | EA401R | E401R |  | 7.5 | 5.49 | 0.163945464 | -0.169841501 |
| 3L15 | YB442H | Y442H | 298 | 7.4 | -3.23 | -0.885324801 | 0.885282111 |
| 3MON | RB39G | R39G | 298.15 | 5.5 | 1.194 | -0.480721321 | 0.481189516 |
| 3MON | WB3C | W3C | 298.15 | 5.5 | -2.866 | 0.074506233 | -0.08457146 |
| 3O39 | FA115I | F115I | 298.15 | 7.4 | -1.81 | 0.235666034 | -0.234605912 |
| 3O39 | FA115L | F115L | 298.15 | 7.4 | -1.64 | 0.298494809 | -0.306041674 |
| 3O39 | HA96L | H96L | 298.15 | 7.4 | -0.58 | 0.268891772 | -0.26420987 |
| 3O39 | LA32P | L32P | 298.15 | 7.4 | -3.25 | -1.463424277 | 1.52718408 |
| 3O39 | QA100L | Q100L | 298.15 | 7.4 | -2 | 0.602361956 | -0.600879559 |
| 3O39 | QA49L | Q49L | 298.15 | 7.4 | -0.05 | 0.413785934 | -0.423452858 |
| 3S4M | DA122Y | D122Y | 298.15 | 7 | -1.3 | 0.454334203 | -0.460757501 |
| 3S4M | GA130V | G130V | 298.15 | 7 | -2.5 | 1.195896085 | -1.199799682 |
| 3S4M | IA154F | I154F | 298.15 | 7 | 0.096 | -0.288782763 | 0.29481242 |
| 3S4M | WA155R | W155R | 298.15 | 7 | -0.693 | 0.202109354 | -0.231947982 |
| 3S92 | HA395R | H395R | 283.15 | 7.5 | 2.03 | 0.253308513 | -0.262203987 |
| 4BJX | AA89V | A89V | 283.15 | 7.5 | 0.99 | 0.474430113 | -0.4782 |
| 4BUQ | YA137A | Y137A | 298.15 | 7.4 | 0.89 | -0.944050033 | 0.936209512 |
| 4BUQ | YA48A | Y48A | 298.15 | 7.4 | -1.435 | -0.932358548 | 0.941298034 |
| 4HE7 | AA19D | A19D | 298.15 | 7.6 | -0.24 | 0.555199644 | -0.551445162 |
| 4HE7 | AA19G | A19G | 298.15 | 7.6 | 0.06 | -0.254705025 | 0.25224926 |
| 4HE7 | AA19K | A19K | 298.15 | 7.6 | -0.46 | 0.464072861 | -0.460723161 |
| 4N6V | HA75W | H75W | 298.15 | 6 | 0.574 | 0.261516337 | -0.257324027 |
| 4N6V | PA36G | P36G | 298.15 | 6 | -1.362 | -0.345084318 | 0.344671892 |
| 4N6V | PA79S | P79S | 298.15 | 6 | 1.745 | -0.067915715 | 0.065280002 |
| 4WAA | KA65A | K65A | 298.15 | 7 | 0.1 | -0.478917269 | 0.475028619 |
| 4WAA | KA67A | K67A | 298.15 | 7 | -2.07 | -0.73582079 | 0.741220229 |
| 4WAA | RA27A | R27A | 298.15 | 7 | -0.44 | -0.486498985 | 0.487297529 |
| 4YEE | VA134T | V134T | 298.15 | 7.5 | -0.71 | -0.862237701 | 0.865822387 |
| 4YEF | TA134V | T134V | 298.15 | 7.5 | -0.93 | 0.808039374 | -0.797285233 |
| 5JXB | DA329G | D329G |  | 7.5 | -1.44 | -0.421336825 | 0.422112423 |
| 5JXB | DA329P | D329P |  | 7.5 | -1.44 | -0.032990572 | 0.039408678 |
| 5OAQ | YA429H | Y429H | 298 | 7.4 | -2.99 | -0.867287567 | 0.864577604 |
| 5VP3 | RA39K | R39K | 298.15 | 8 | 0.413 | 0.210735649 | -0.212911675 |
| 5VP3 | SA128G | S128G | 298.15 | 8 | -0.378 | -0.68591244 | 0.688037606 |
| 5VP3 | VA183T | V183T | 298.15 | 8 | 0.354 | -0.672133122 | 0.681911724 |

Supplementary Table 6. The myoglobin dataset with detailed prediction of mutDDG-SSM.

| pdb_code | position | wild_type | mutant | ΔΔG(kcal/mol) | direct_prediction | inverse_prediction |
| --- | --- | --- | --- | --- | --- | --- |
| 1bz6A | 28 | I | A | 2.06 | -1.6044018 | 1.5211675 |
| 1bz6A | 29 | L | A | 0.39 | -2.4699855 | 2.4678357 |
| 1bz6A | 30 | I | A | 1.9 | -1.6488411 | 1.5483963 |
| 1bz6A | 32 | L | A | 2.04 | -1.9802034 | 1.9450096 |
| 1bz6A | 24 | H | V | 0.52 | 0.33493686 | -0.076779746 |
| 1bz6A | 36 | H | Q | 1.3 | -0.25506464 | 0.16743153 |
| 1bz6A | 48 | H | Q | 0.62 | 0.43765646 | -0.17077951 |
| 1bz6A | 64 | H | Q | 0.45 | -0.14210624 | 0.28212136 |
| 1bz6A | 82 | H | Q | 0.05 | -0.45166868 | 0.42598024 |
| 1bz6A | 93 | H | G | -0.04 | -0.89262855 | 0.7004056 |
| 1bz6A | 97 | H | Q | 0.11 | -0.007558876 | -0.030634 |
| 1bz6A | 113 | H | Q | 0.26 | -0.15818402 | 0.0753739 |
| 1bz6A | 119 | H | F | 0.68 | -0.27242988 | 0.09501653 |
| 1bz6A | 129 | G | A | -1.05 | 0.78661853 | -0.80288446 |
| 1bz6A | 66 | V | A | -0.75 | -0.17395122 | 0.23543692 |
| 1bz6A | 88 | P | A | -0.59 | -0.05760507 | 0.001467818 |
| 1bz6A | 116 | H | A | -0.16 | -0.25979444 | 0.26038584 |
| 1bz6A | 13 | V | A | 0.67 | -0.9949089 | 0.8182751 |
| 1bz6A | 67 | T | A | 0.26 | -0.41719738 | 0.35699785 |
| 1bz6A | 117 | S | A | 0.26 | -0.04017338 | 0.14145514 |
| 1bz6A | 11 | L | A | 0.44 | -0.7445539 | 0.847551 |
| 1bz6A | 9 | L | A | 0.41 | -1.3981625 | 1.4732604 |
| 1bz6A | 23 | G | A | 1.12 | 0.56470954 | -0.608112 |
| 1bz6A | 137 | L | A | 1.78 | -1.7971709 | 1.8155453 |
| 1bz6A | 114 | V | A | 1.45 | -1.112006 | 1.1934531 |
| 1bz6A | 51 | T | A | 1.41 | -0.9327318 | 0.86159813 |
| 1bz6A | 149 | L | A | 1.6 | -1.5276346 | 1.265215 |
| 1bz6A | 142 | I | A | 1.92 | -1.5623802 | 1.6813705 |
| 1bz6A | 8 | Q | A | 0.89 | -0.015894517 | 0.009448586 |
| 1bz6A | 8 | Q | G | -0.5 | -0.7671804 | 0.91617393 |
| 1bz6A | 109 | E | A | 0.17 | 0.04684227 | -0.11725088 |
| 1bz6A | 109 | E | G | -0.89 | -1.0769851 | 1.1520454 |
| 1bz6A | 140 | K | A | 0.35 | -0.1923373 | 0.19850215 |
| 1bz6A | 29 | L | F | 0.13 | -0.8486146 | 0.7825893 |
| 1bz6A | 29 | L | N | 5.59 | -2.123197 | 2.533408 |
| 1bz6A | 43 | F | V | 2.66 | -0.27855724 | 0.24378936 |
| 1bz6A | 43 | F | I | 0.89 | 0.37161413 | -0.3942276 |
| 1bz6A | 64 | H | Q | 0.54 | -0.14160195 | 0.28556755 |
| 1bz6A | 64 | H | A | -0.58 | -0.16684972 | 0.013076564 |
| 1bz6A | 64 | H | L | -1.05 | 0.75047505 | -0.7326784 |
| 1bz6A | 64 | H | F | -1.51 | 1.3364502 | -1.3244913 |
| 1bz6A | 68 | V | F | -0.82 | -0.04019738 | -0.021120604 |
| 1bz6A | 68 | V | A | 0.93 | -1.0240643 | 0.9011773 |
| 1bz6A | 68 | V | Q | 2.42 | -0.96419203 | 1.2393379 |
| 1bz6A | 68 | V | N | 3.81 | -1.3879683 | 1.4011166 |
| 1bz6A | 68 | V | S | 2.99 | -1.6088603 | 1.4771366 |
| 1bz6A | 68 | V | T | 1.67 | -1.0959213 | 1.3739574 |
| 1bz6A | 107 | I | F | -0.58 | -0.6273531 | 0.9737145 |
| 1bz6A | 107 | I | V | 1.09 | -0.43848962 | 0.46586627 |
| 1bz6A | 107 | I | T | 2.23 | -1.0850418 | 1.0651547 |
| 1bz6A | 28 | I | L | 0.55 | -0.22526732 | 0.50590926 |
| 1bz6A | 28 | I | M | 0.56 | -0.56218684 | 0.6396299 |
| 1bz6A | 28 | I | V | -0.04 | -0.956439 | 0.94374245 |
| 1bz6A | 28 | I | A | 1.33 | -1.6068113 | 1.5268319 |
| 1bz6A | 111 | I | L | 0.64 | 0.118912436 | -0.20241837 |
| 1bz6A | 111 | I | M | 1.14 | 0.07405975 | -0.23654246 |
| 1bz6A | 111 | I | A | 1.84 | -1.1495944 | 0.89380753 |
| 1bz6A | 142 | I | L | -0.63 | 0.17366444 | -0.4513751 |
| 1bz6A | 142 | I | M | -0.93 | 0.42072806 | -0.35780835 |
| 1bz6A | 142 | I | V | 0.12 | -0.34259754 | 0.3000832 |
| 1bz6A | 142 | I | A | 1.1 | -1.5651668 | 1.678441 |
| 1bz6A | 29 | L | I | 1.12 | -0.3000417 | 0.35127115 |
| 1bz6A | 29 | L | M | -0.12 | -0.26469868 | 0.43611604 |
| 1bz6A | 29 | L | V | 1.72 | -1.0990647 | 1.0490332 |
| 1bz6A | 29 | L | A | 2.37 | -2.466063 | 2.476667 |
| 1bz6A | 69 | L | I | 0.02 | 0.020511184 | 0.22012183 |
| 1bz6A | 69 | L | M | 0 | -0.08729547 | -0.013881478 |
| 1bz6A | 69 | L | V | 0.1 | -0.44122475 | 0.5548607 |
| 1bz6A | 69 | L | A | 1.18 | -1.9029264 | 1.9427078 |
| 1bz6A | 135 | L | I | 1.54 | -0.49176675 | 0.31411275 |
| 1bz6A | 135 | L | M | 0.79 | -0.45163465 | 0.2810858 |
| 1bz6A | 135 | L | V | 2.25 | -0.36107415 | 0.4665113 |
| 1bz6A | 49 | L | I | 0.8 | -0.26206127 | 0.29039684 |
| 1bz6A | 130 | A | L | 2.3 | 0.9896372 | -0.9918426 |
| 1bz6A | 130 | A | K | 3.7 | -0.50737035 | 0.6205965 |
| 1bz6A | 123 | F | T | 3.5 | -1.1087816 | 1.0423868 |
| 1bz6A | 4 | E | A | 0.35 | -0.56367576 | 0.45975608 |
| 1bz6A | 133 | K | A | 0.05 | 0.12122877 | -0.073852375 |
| 1bz6A | 122 | D | A | 0.1 | -0.23595831 | 0.23501715 |
| 1bz6A | 18 | E | A | 0.95 | -0.54013324 | 0.37739715 |
| 1bz6A | 77 | K | A | -0.2 | -0.07508933 | -0.1073672 |
| 1bz6A | 20 | D | A | 0.5 | -0.3088965 | 0.3191895 |
| 1bz6A | 118 | R | A | 0.65 | -0.75519705 | 0.6645486 |
| 1bz6A | 44 | D | A | -0.25 | -0.39663988 | 0.3916949 |
| 1bz6A | 60 | D | A | 0.15 | 0.05361586 | -0.05817639 |
| 1bz6A | 56 | K | A | 0.35 | -0.2715283 | 0.3939804 |
| 1bz6A | 139 | R | A | 0.45 | -1.0451305 | 1.3596141 |
| 1bz6A | 7 | W | F | 0.9 | -0.6852184 | 0.7695305 |
| 1bz6A | 14 | W | F | 1.1 | -0.21151534 | 0.043841194 |
| 1bz6A | 131 | M | A | 2.2 | -1.8048084 | 1.6825116 |
| 1bz6A | 123 | F | K | 2.1 | -1.0415505 | 1.0623573 |
| 1bz6A | 130 | A | K | 2.1 | -0.54349124 | 0.5686638 |
| 1bz6A | 130 | A | L | 0.9 | 0.9941777 | -0.9927441 |
| 1bz6A | 36 | H | Q | 0.8 | -0.259047 | 0.1604409 |
| 1bz6A | 68 | V | T | 0.6 | -1.1158736 | 1.3964831 |
| 1bz6A | 13 | V | A | 0 | -0.9974594 | 0.8232708 |
| 1bz6A | 115 | L | A | 1.4 | -1.9415467 | 2.2180324 |
| 1bz6A | 123 | F | A | 1.1 | -1.5614173 | 1.4679686 |
| 1bz6A | 135 | L | A | 1.7 | -1.2618177 | 1.2644777 |
| 1bz6A | 11 | L | A | 0.6 | -0.7419956 | 0.8567427 |
| 1bz6A | 106 | F | A | 0.7 | -1.6634357 | 1.6368942 |
| 1bz6A | 137 | L | A | 0.3 | -1.7903903 | 1.8119657 |
| 1bz6A | 15 | A | L | -0.1 | 0.8813745 | -0.9809349 |
| 1bz6A | 125 | A | L | -0.6 | 0.9057975 | -0.8255159 |
| 1bz6A | 144 | A | L | -0.4 | 0.890228 | -0.76186645 |
| 1bz6A | 28 | I | A | 0.53 | -1.6033516 | 1.5238498 |
| 1bz6A | 28 | I | F | 0.28 | -0.7087327 | 0.8175985 |
| 1bz6A | 32 | L | W | 0.53 | -0.002455267 | -0.30539426 |
| 1bz6A | 32 | L | A | 0.47 | -1.9933599 | 1.9500176 |
| 1bz6A | 32 | L | F | 0.07 | 0.01686495 | -0.28486183 |
| 1bz6A | 64 | H | F | -0.81 | 1.3479247 | -1.3204701 |
| 1bz6A | 64 | H | A | -0.49 | -0.19196704 | 0.016577289 |
| 1bz6A | 64 | H | W | -0.47 | 1.4338048 | -1.6628015 |
| 1bz6A | 64 | H | Q | -0.02 | -0.1419259 | 0.28223616 |
| 1bz6A | 64 | H | Y | -0.52 | 1.2121347 | -1.4180236 |
| 1bz6A | 64 | H | L | -0.61 | 0.75865096 | -0.74471074 |
| 1bz6A | 64 | H | I | -0.38 | 1.2837684 | -1.2201566 |
| 1bz6A | 64 | H | V | -0.36 | 0.7180618 | -0.7837005 |
| 1bz6A | 64 | H | T | -0.23 | 0.3111349 | -0.3081509 |
| 1bz6A | 64 | H | N | 0.33 | 0.3709947 | -0.31742662 |
| 1bz6A | 64 | H | R | 0.08 | 0.9811532 | -0.9148224 |
| 1bz6A | 64 | H | G | 0.04 | -0.88509387 | 0.96156263 |
| 1bz6A | 67 | T | F | -0.19 | 0.24583724 | -0.15872727 |
| 1bz6A | 67 | T | Q | -0.23 | -0.2438437 | 0.303415 |
| 1bz6A | 67 | T | P | 0.85 | -1.1424096 | 1.2854421 |
| 1bz6A | 68 | V | F | -0.38 | -0.023927374 | -0.020431003 |
| 1bz6A | 68 | V | L | -0.42 | 0.26989532 | -0.23529303 |
| 1bz6A | 68 | V | W | 0.07 | 0.11122086 | 0.18949565 |
| 1bz6A | 68 | V | I | -0.35 | 0.4102025 | -0.1478657 |
| 1bz6A | 68 | V | A | 0.6 | -1.0339099 | 0.8961231 |
| 1bz6A | 68 | V | Y | 0.17 | 0.15033665 | 0.14186078 |
| 1bz6A | 68 | V | T | 0.57 | -1.131699 | 1.3809857 |
| 1bz6A | 107 | I | F | -0.12 | -0.62732077 | 0.9737145 |
| 1bz6A | 107 | I | A | 0.43 | -1.6706238 | 1.7843072 |

Supplementary Table 7. The p53 dataset with detailed prediction of mutDDG-SSM.

| pdb_code | position | wild_type | mutant | ΔΔG(kcal/mol) | direct_prediction | inverse_prediction |
| --- | --- | --- | --- | --- | --- | --- |
| 2ocjA | 104 | Q | H | -0.24 | 0.5778896 | -0.37147102 |
| 2ocjA | 104 | Q | P | -0.11 | -0.1456513 | 0.005863526 |
| 2ocjA | 123 | T | A | 0.13 | -0.57709056 | 0.56258863 |
| 2ocjA | 129 | A | D | 0.7 | 0.2588594 | -0.34277397 |
| 2ocjA | 129 | A | E | 0.38 | 0.32904223 | -0.5526665 |
| 2ocjA | 129 | A | S | 0.19 | 0.41428742 | -0.46761757 |
| 2ocjA | 133 | M | L | -0.3 | 0.4410163 | -0.64505064 |
| 2ocjA | 134 | F | L | 4.78 | -0.36545366 | 0.4923338 |
| 2ocjA | 143 | V | A | 3.5 | -1.8851795 | 1.8664751 |
| 2ocjA | 145 | L | Q | 2.98 | -2.1533036 | 2.1734424 |
| 2ocjA | 148 | D | E | 0.43 | 0.4327283 | -0.27119935 |
| 2ocjA | 148 | D | S | -0.22 | -0.28206265 | 0.09696122 |
| 2ocjA | 150 | T | P | 0.08 | 0.10783 | 0.09397178 |
| 2ocjA | 151 | P | S | 4.49 | -0.6101694 | 0.6286404 |
| 2ocjA | 157 | V | F | 3.88 | 0.079864584 | 0.04059767 |
| 2ocjA | 165 | Q | K | 1.27 | 0.15262954 | -0.0503268 |
| 2ocjA | 167 | Q | E | 0.43 | 0.09748691 | -0.118444964 |
| 2ocjA | 168 | H | R | 2.75 | 0.05628019 | 0.104339324 |
| 2ocjA | 174 | R | K | 0.22 | -0.6311221 | 0.596437 |
| 2ocjA | 175 | R | A | 0.73 | -0.99827087 | 1.2006638 |
| 2ocjA | 175 | R | H | 3.52 | -0.84883416 | 0.9223634 |
| 2ocjA | 182 | C | S | -0.16 | 0.10059009 | -0.0753686 |
| 2ocjA | 195 | I | T | 4.12 | -2.3150203 | 2.3873212 |
| 2ocjA | 201 | L | P | -0.35 | -0.9653114 | 0.9586028 |
| 2ocjA | 203 | V | A | -0.49 | -0.8109191 | 0.8716791 |
| 2ocjA | 206 | L | S | 0.1 | -1.0611336 | 1.0190421 |
| 2ocjA | 220 | Y | C | 3.98 | -0.07104169 | -0.015446979 |
| 2ocjA | 228 | D | E | -0.05 | 0.28251523 | -0.26362422 |
| 2ocjA | 232 | I | T | 3.19 | -1.7424889 | 1.6342785 |
| 2ocjA | 236 | Y | F | -0.27 | -0.10504869 | 0.26575893 |
| 2ocjA | 237 | M | I | 3.18 | 0.32085285 | -0.27077612 |
| 2ocjA | 239 | N | Y | -1.49 | 0.9243867 | -0.6229569 |
| 2ocjA | 242 | C | S | 3.07 | -1.3834069 | 1.1899278 |
| 2ocjA | 245 | G | S | 1.21 | 0.29494864 | -0.49766707 |
| 2ocjA | 248 | R | Q | 1.87 | -0.58827055 | 0.62197745 |
| 2ocjA | 249 | R | S | 1.92 | -1.0497777 | 0.8006288 |
| 2ocjA | 255 | I | F | 3.29 | -0.28598353 | 0.44411093 |
| 2ocjA | 260 | S | P | 0.32 | 0.27683058 | -0.5798358 |
| 2ocjA | 268 | N | D | -1.21 | -0.48782715 | 0.15131068 |
| 2ocjA | 270 | F | C | 4.54 | -0.49458677 | 0.7265004 |
| 2ocjA | 273 | R | H | 0.45 | -0.5616375 | 0.83857334 |
| 2ocjA | 282 | R | W | 3.3 | 0.3471729 | -0.27521417 |

Supplementary Table 8. PCC of the predicted and experimental ΔΔG values for the mutations involving alanine and glycine, which are compared with the results of all the mutations in the datasets.

| Dataset | All mutations | | Mutations involving Ala | | Mutations involving Gly | |
| --- | --- | --- | --- | --- | --- | --- |
|  | Number of mutations | PCC | Number of mutations | PCC | Number of mutations | PCC |
| S^sym^ | 642 | 0.73 | 194 | 0.72 | 60 | 0.90 |
| S347(including S203) | 347 | 0.70 | 182 | 0.71 | 100 | 0.74 |

PCC stands for Pearson correlation coefficient.

Supplementary Table 9. PCC values obtained by different machine learning methods on the five test datasets used in our study, which were compared with those obtained by XGBoost.

|  | XGBoost | Decision Tree | SVM | Random Forest | ANN |
| --- | --- | --- | --- | --- | --- |
| S^sym^ | 0.73 | 0.48 | 0.60 | 0.52 | 0.70 |
| S203 | 0.74 | 0.53 | 0.64 | 0.58 | 0.67 |
| S347 | 0.70 | 0.46 | 0.57 | 0.49 | 0.64 |
| myoglobin | 0.66 | 0.47 | 0.53 | 0.47 | 0.64 |
| p53 | 0.56 | 0.47 | 0.52 | 0.41 | 0.54 |


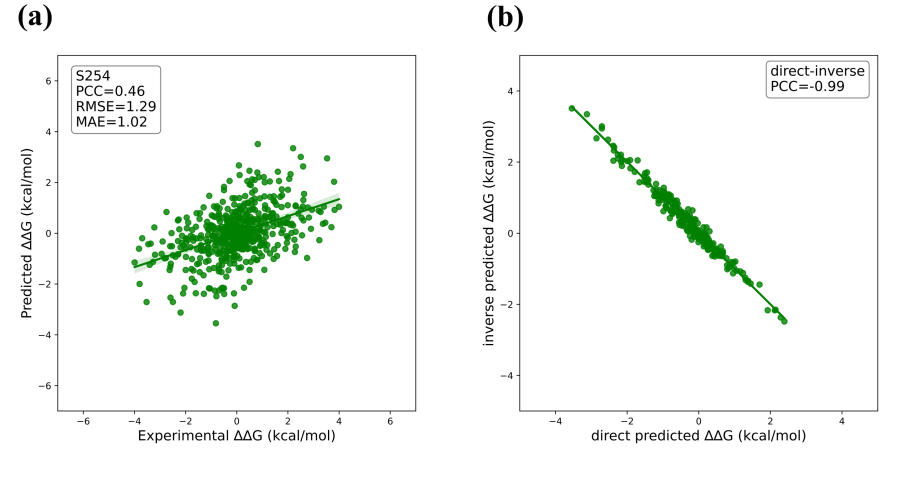


Supplementary Figure 1. The performance of mutDDG-SSM in predicting ∆∆G on S254 dataset. (a) The performance of mutDDG-SSM on S254 dataset. (b) Prediction bias of mutDDG-SSM on S254 dataset.


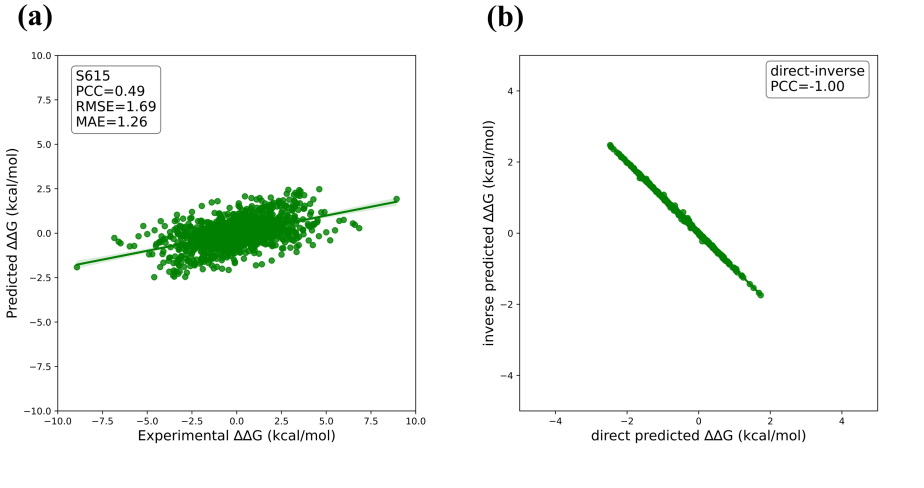


Supplementary Figure 2. The performance of mutDDG-SSM in predicting ∆∆G on S615 dataset. (a) The performance of mutDDG-SSM on S615 dataset. (b) Prediction bias of mutDDG-SSM on S615 dataset.
